# A Versatile Method for Site-Specific Chemical Installation of Aromatic Posttranslational Modification Analogs into Proteins

**DOI:** 10.1021/jacs.4c08416

**Published:** 2024-09-03

**Authors:** Xiaoxi Lin, Shaswati Mandal, Raj V. Nithun, Rajasekhar Kolla, Bouchra Bouri, Hilal A. Lashuel, Muhammad Jbara

**Affiliations:** † School of Chemistry, Raymond and Beverly Sackler Faculty of Exact Sciences, Tel Aviv University, Tel Aviv 69978, Israel; ‡ Laboratory of Molecular and Chemical Biology of Neurodegeneration, Institute of Bioengineering, School of Life Sciences, École Polytechnique Fédérale de Lausanne, Lausanne CH-1015, Switzerland; § Protein Production and Structure core facility, School of Life Sciences, École Polytechnique Fédérale de Lausanne, Lausanne CH-1015, Switzerland

## Abstract

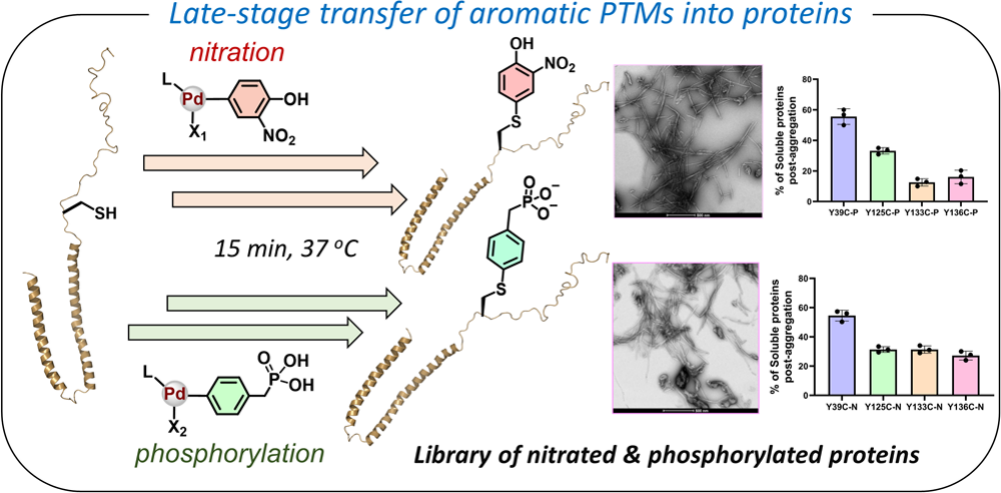

Posttranslational modifications (PTMs) of proteins play central roles in regulating the protein structure, interactome, and functions. A notable modification site is the aromatic side chain of Tyr, which undergoes modifications such as phosphorylation and nitration. Despite the biological and physiological importance of Tyr-PTMs, our current understanding of the mechanisms by which these modifications contribute to human health and disease remains incomplete. This knowledge gap arises from the absence of natural amino acids that can mimic these PTMs and the lack of synthetic tools for the site-specific introduction of aromatic PTMs into proteins. Herein, we describe a facile method for the site-specific chemical installation of aromatic PTMs into proteins through palladium-mediated S–C(sp^2^) bond formation under ambient conditions. We demonstrate the incorporation of novel PTMs such as Tyr-nitration and phosphorylation analogs to synthetic and recombinantly expressed Cys-containing peptides and proteins within minutes and in good yields. To demonstrate the versatility of our approach, we employed it to prepare 10 site-specifically modified proteins, including nitrated and phosphorylated analogs of Myc and Max proteins. Furthermore, we prepared a focused library of site-specifically nitrated and phosphorylated α–synuclein (α-Syn) protein, which enabled, for the first time, deciphering the role of these competing modifications in regulating α-Syn conformation aggregation in vitro. Our strategy offers advantages over synthetic or semisynthetic approaches, as it enables rapid and selective transfer of rarely explored aromatic PTMs into recombinant proteins, thus facilitating the generation of novel libraries of homogeneous posttranslationally modified proteins for biomarker discovery, mechanistic studies, and drug discovery.

## Introduction

Posttranslational modifications (PTMs) of proteins play central roles in controlling numerous biological processes essential for the proper functionality of living cells.^1^ The incorporation of PTMs into proteins and their removal from proteins are a dynamic process that is tightly regulated by enzymes known as “writers” and “erasers”. The diverse nature of these modifications greatly contributes to expanding the structural and functional diversity of the proteome.^2^ Several amino acids in proteins are subjected to a wide range of PTMs, and different types of PTMs often compete for the same amino acid. Of particular interest are the tyrosine (Tyr) residues, which are known to undergo posttranslational modifications such as phosphorylation,^3^ sulfation,^4^ or nitration.^5^ Tyr-PTMs play important roles in regulating signal transduction, cell growth, differentiation, metabolism, regulation of enzymatic activity, and protein–protein interactions.^6^ Dysregulation of Tyr-PTMs is implicated in the development and progression of various human diseases including cancer and neurological disorders.^3,6^ However, dissecting the role of Tyr-PTMs in regulating the protein function in health and disease remains challenging and has been hampered by the lack of methodologies that allow for the site-specific introduction of Tyr modifications in vitro or investigating the dynamics of competing PTMs on Tyr residues in cells. Despite the significant advances in the chemical and semisynthetic strategies for producing homogeneously modified proteins, the incorporation of aromatic PTMs into proteins using common chemical biology tools remains largely limited and challenging,^7^ which has hampered efforts to decipher the PTM codes of proteins.^8^

**Figure 1:**
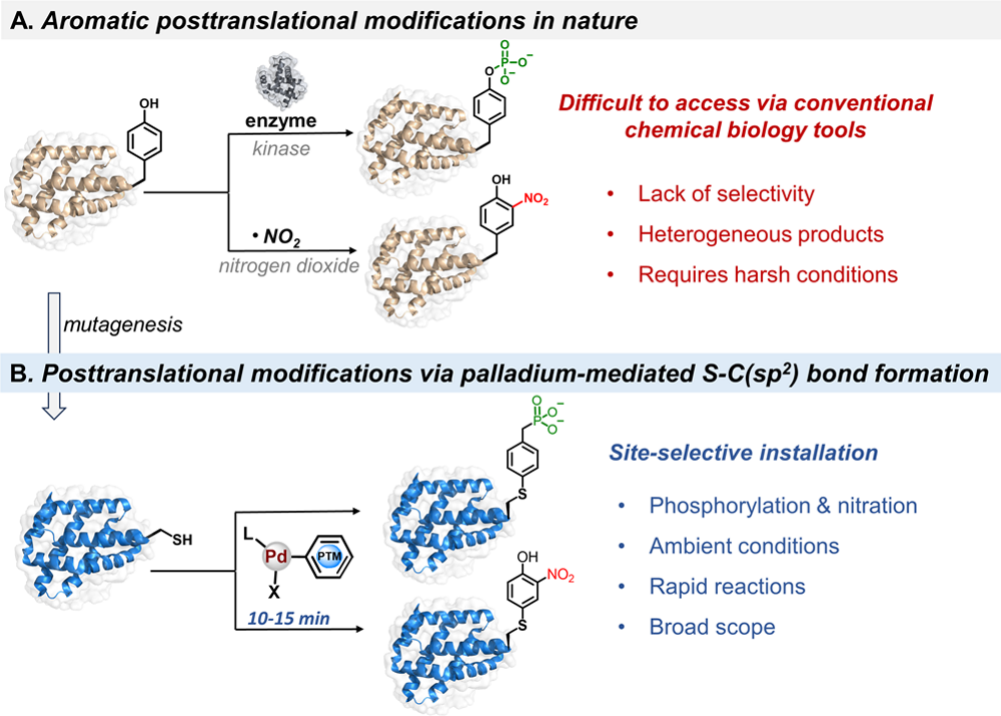
Palladium-mediated site-specific installation of aromatic PTM analogs into proteins. (A) Aromatic posttranslational modifications of proteins in nature, e.g., Tyr phosphorylation and nitration. (B) Site-specific late-stage installation of Tyr phosphorylation and nitration mimics into proteins.

Although it is widely accepted that phosphorylation at serine and threonine residues could be studied using amino acids that partially mimic the charge state of the phosphorylated forms of these residues, serine to aspartate or threonine to glutamate, there are no natural amino acids that can mimic aromatic PTMs. Furthermore, methods that allow ease and flexibility in the site-specific incorporation of these PTMs into proteins, especially natively structured proteins, in high homogeneity and workable quantities remain lacking. In vitro enzymatic modification methods often lack specificity, resulting in heterogeneous mixtures of modified proteins, which makes it difficult to decipher the relative contribution of Tyr modifications at specific residues or the cross-talk between specific residues. Furthermore, limited knowledge about the natural enzymes responsible for regulating Tyr modification presents additional challenges in deciphering their role in health and disease. Other biological approaches such as the genetic-code expansion method provide a powerful means to generate site-specifically modified proteins^9^; however, this method is technically challenging and is difficult to apply to produce proteins bearing multiple PTMs in sufficient quantities.^10^ Chemical protein synthesis and chemoselective protein ligation approaches represent powerful methods for addressing these limitations^11^ and have been successfully used to decipher the molecular role of PTMs of a large number of proteins of various sizes, along with functional and structural complexity.^12^ However, one of the limitations of these approaches is that introducing different modifications at the same residue often requires repeating the entire protein synthesis/semisynthesis, which is often time-consuming and challenging, especially when ligation reactions are carried out under denaturing conditions and proper refolding of the proteins is required.^13^ Therefore, there is a need to develop new and versatile methods that enable the rapid site-specific introduction of different types of PTMs, starting with properly folded proteins. We envisioned the development of late-stage and the orthogonal insertion of aromatic PTMs into native proteins to enable an unprecedented opportunity to generate libraries of homogeneously modified analogs. In principle, this process can enable rapid access to proteins bearing defined aromatic PTMs at the desired sites through direct protein diversification of recombinantly expressed proteins (Figure 1).

Late-stage protein modification approaches are ideal to dissect the role of PTMs that compete for the same residue, e.g., Tyr phosphorylation and nitration.^14^ The low abundance and nucleophilicity of Cys residue have rendered it the site of choice to install site-specific modifications, labeling or aliphatic PTM mimics into proteins, mainly through S-alkylation and thiol-ene chemistry to provide the desired modified protein with a single atom (−S−) variation.^15^ Cys elimination to dehydroalanine (Dha) has also been explored to install aliphatic PTM mimics via Michael addition or carbon–carbon bond-forming reactions.^16^ The current late-stage protein modification approaches using Cys and Dha chemistry have provided unprecedented opportunities to modify proteins with specific PTMs. However, these strategies were implemented to incorporate primarily aliphatic PTM mimics such as Lys/Arg methylation, Lys acetylation, Ser phosphorylation, and glycosylation into proteins.^15,16^ Recently, Cys arylation has emerged as a powerful method for functionalizing biomolecules with high precision.^17^ In this context, S-arylation using transition metals is particularly noteworthy for its high reaction rate and chemoselectivity under ambient conditions.^18^ Herein, we report a facile and site-selective strategy to transfer aromatic PTM analogs to proteins through the Cys residue via palladium(II)-mediated S–C(sp^2^) bond formation reactions. We demonstrate the power of this approach to incorporate Tyr-nitration and phosphorylation at diverse sites and show that this approach could be used to produce homogeneously modified proteins in good yield and multimilligram quantities. Furthermore, this method enabled the preparation of a focused library of site-specifically nitrated or phosphorylated α-synuclein (α-Syn) analogs, enabling for the first time the investigation and comparison of the effect of these competing modifications with all Tyr residues in this protein, which plays a central role in the pathogenesis of Parkinson’s disease and other neurodegenerative disorders.^19^

## Results and Discussion

### Design of Palladium(II) Oxidative Addition Complexes to Install Aromatic PTMs to Unprotected Peptides

**Figure 2:**
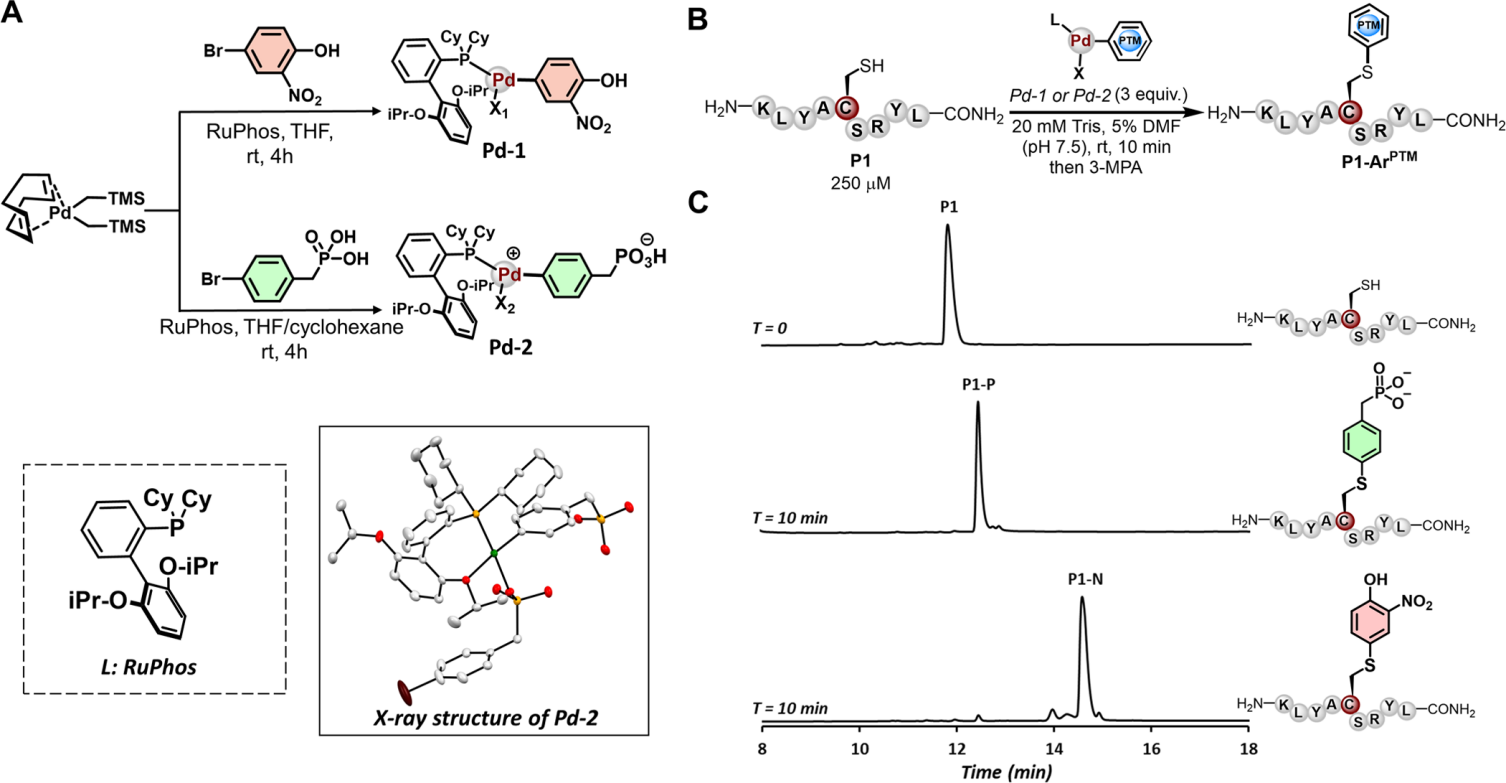
Late-stage insertion of phosphorylation and nitration mimics to unprotected peptides using the palladium(II) strategy. (A) Schematic representation of the synthesis of palladium(II) oxidative addition complexes bearing aromatic PTMs and the X-ray structure of **Pd-2** (CCDC: 2362585). (B) Schematic representation of the site-specific installation of phosphorylation and nitration mimics to unprotected peptides. (C) Analytical HPLC trace of the crude reactions after 10 min. The reactions were quenched with 3-mercaptopropionic acid (3-MPA) before HPLC analysis. Analytical RP-HPLC was performed by using 0.05% trifluoroacetic acid in water and acetonitrile as the mobile phases. Modified peptides were obtained in a protonated form. X_1_: bromide; X_2_: 4-bromobenzylphosphonic acid.

Organometallic palladium chemistry has emerged as a powerful means to functionalize a wide range of biopolymers with novel molecules.^20^ Of particular interest are palladium(II) oxidative addition complexes (Pd(II)OACs), which enable rapid and selective Cys functionalization in proteins through a C-S cross-coupling process.^18a,21^ We envisaged the design of Pd(II)OACs embedded with aromatic PTM scaffolds to allow the installation of target PTM mimics through Cys residues (Figure 2).^22^ Remarkably, the high reaction rate, selectivity, and aqueous compatibility of Pd(II)OACs,^23^ along with the orthogonal reactivity and low abundance of Cys, should in principle provide a powerful means to enable precise and rapid transfer of aromatic PTMs to unprotected peptides and proteins under aqueous conditions.

To test our hypothesis, we first set out to synthesize Pd(II)OAC bearing the 2-nitrophenol moiety to transfer the Tyr-nitration mimic (Figure 2A). By combining the bis[(trimethylsilyl)methyl](1,5-cyclooctadiene)palladium(II) [(COD)Pd(CH_2_TMS)_2_] precursor and 4-bromo-2-nitrophenol with RuPhos ligand, we were able to synthesize the desired **Pd-1** complex, obtained in 88% isolated yield (see the Supporting Information, Section 2). To prepare the phosphorylation analog, we opted to develop a stable phospho-Tyr mimic with minimal alteration at the modification site (e.g., O-to-CH_2_ substitution). This approach should provide a hydrolysis-stable mimic that is compatible with both in vitro and in vivo studies. To this end, we reacted commercially available 4-bromobenzylphosphonic acid with [(COD)Pd(CH_2_TMS)_2_] in the presence of RuPhos, which provided the desired **Pd-2** complex in 61% isolated yield (Figure 2A).

Both palladium complexes **Pd-1** and **Pd-2** exhibited superior reactivity, facilitating the effective transfer of nitration and phosphorylation analogs to Cys-containing peptides within minutes. Initially, we tested the reactivity of the **Pd-1** and **Pd-2** analogs with a 9-mer polypeptide (KLYACSRYL, **P1**), prepared using a standard Fmoc-SPPS (see the Supporting Information, Section 4.1). We were pleased to observe a rapid and effective incorporation of the aromatic PTM analogs to **P1** using a low loading of **Pd-1** and **Pd-2** (3 equiv) in 20 mM Tris buffer (pH 7.5) using 5% DMF cosolvent. This led to a quantitative conversion to the desired site-specifically modified products within less than 10 min at room temperature, as determined by analytical high-performance liquid chromatography–mass spectrometry (HPLC–MS) analysis (Figure 2B,C). These findings indicate that designed Pd(II)OACs could effectively insert aromatic PTM mimics into unprotected peptides under mild conditions, highlighting the power of this strategy to transfer aromatic PTMs to complex biomacromolecules.

### Palladium(II)-Mediated Site-Selective Installation of Phosphorylation and Nitration Analogs into Proteins

**Figure 3:**
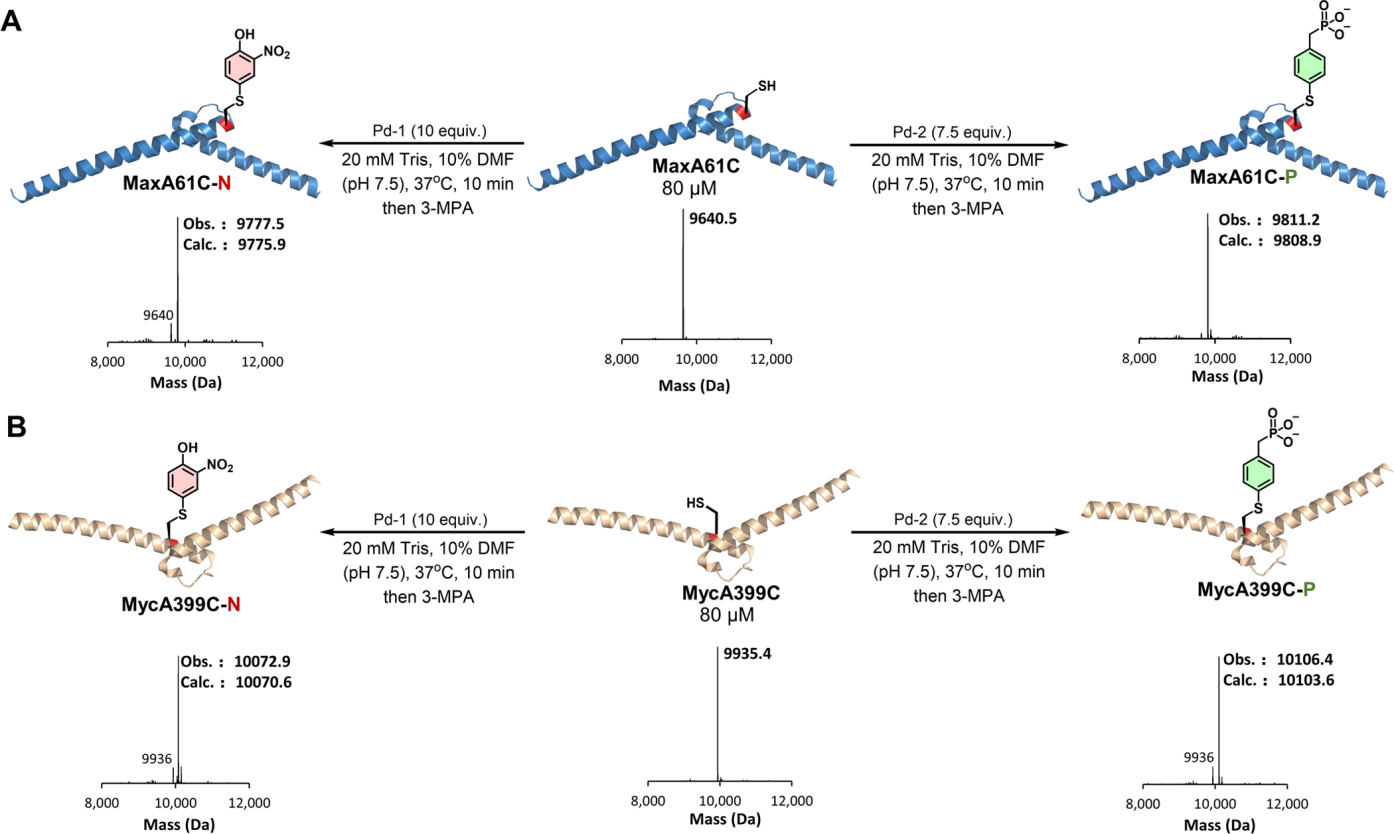
Site-specific installation of phosphorylation and nitration PTM analogs into proteins via palladium-mediated S–C(sp^2^) bond formation. (A) Monophosphorylated and nitrated **MaxA61C** protein. The deconvoluted mass spectrum of the starting material and the crude reaction mixtures after 10 min is depicted. (B) Monophosphorylated and nitrated of **MycA399C** protein. The deconvoluted mass spectrum of the starting material and the crude reaction mixtures after 10 min is depicted. LCMS was performed using 0.1% formic acid in water and acetonitrile as the mobile phases. Modified proteins were obtained in the protonated form.

Encouraged by our results, we turned our attention to site-selectively incorporating the phosphorylation and nitration marks into protein at predetermined sites. As a model system, we employed our approach to modify the DNA binding domains of the Myc and Max transcription factors (TFs) engineered with a single Cys residue (**MaxA61C** and **MycA399C**). We initially reacted **MaxA61C** with **Pd-1** or **Pd-2** separately using the optimized reaction conditions in 20 mM Tris buffer and 10% DMF (pH 7.5) at 37 °C (see the Supporting Information, Section 7). These conditions provided the mononitrated and phosphorylated products **MaxA61C-N** and **MaxA61C-P** within 10 min in 86 and 96% conversion, respectively, as determined by LC-MS analysis (Figure 3A). We observed the same outcome once we reacted the **MycA399C** protein with **Pd-1** or **Pd-2** under the optimized conditions, which provided the desired modified products **MycA399C-N** and **MycA399C-P** in 85 and 89% conversion, respectively (Figure 3B). These transformations establish that Tyr-nitration and phosphorylation could be successfully transferred to target proteins through palladium-mediated C-S arylation to provide site-selectively modified proteins. In principle, this late-stage process could enable rapid and effective protein diversification with aromatic PTMs to generate novel libraries of homogeneous posttranslationally modified proteins to dissect their code.

Next, we probed the chemical stability of the nitration and phosphorylation mimics. The PTM analogs in our approach are coupled to proteins through S–C(sp^2^) linkage, which should provide high chemical stability.^18a^ To this end, using **MaxA61C** protein, we produced both modified analogs and analyzed their stability in aqueous buffer (see the Supporting Information, Sections 8.3 and 8.4). We reacted 6.0 mg of **MaxA61C** with **Pd-2** in 20 mM Tris buffer and 5% DMF (pH 7.5) at 37 °C, which provided the desired site-specifically phosphorylated product **MaxA61C-P**. Subsequently, we purified the modified proteins with preparative RP-HPLC to provide **MaxA61C-P** in 65% isolated yield (see the Supporting Information, Section 8.1). Similarly, we successfully isolated the nitrated product **MaxA61C-N** in 64% isolated yield after RP-HPLC purification (see the Supporting Information, Section 8.2). Having both isolated analogs, we incubated **MaxA61C-N** and **MaxA61C-P** separately in phosphate-buffered saline (PBS) at 37 °C. LCMS analysis revealed that no degradation had occurred after 5 days (see the Supporting Information, Sections 8.3 and 8.4). Taken together, these experiments revealed that the installation of phosphorylation and nitration Tyr modifications could be performed on a milligram scale and could provide homogeneously modified proteins in good yield through a stable S-aryl linkage, thus enabling more detailed structural, biochemical, and biological studies to decipher the role of these modifications.

### Site-Specific Incorporation of Nitration and Phosphorylation Analogs into α-Syn Protein

To further assess the effectiveness of our nitro and phosphomimetics in reproducing the effects of bona fide PTMs, we used the synaptic protein α-Syn as a model system, since it offers several advantages: (1) phosphorylation and nitration in several α-Syn Tyr residues have been implicated in pathology formation and neurodegeneration in Parkinson’s disease (PD) and other neurological diseases characterized by α-Syn aggregation and pathology formation,^24^ (2) α-Syn sequences consist of a limited number of Tyr residues, thus allowing the assessment of differential effects of mimicking phosphorylation/nitration at different Tyr residues, and (3) previous studies have already investigated the effect of site-specific phosphorylation/nitration at multiple α-Syn Tyr residues using protein semisynthetic approaches, thus allowing for a direct comparison of our results to data from bona fide modified forms of the protein.

**Figure 4:**
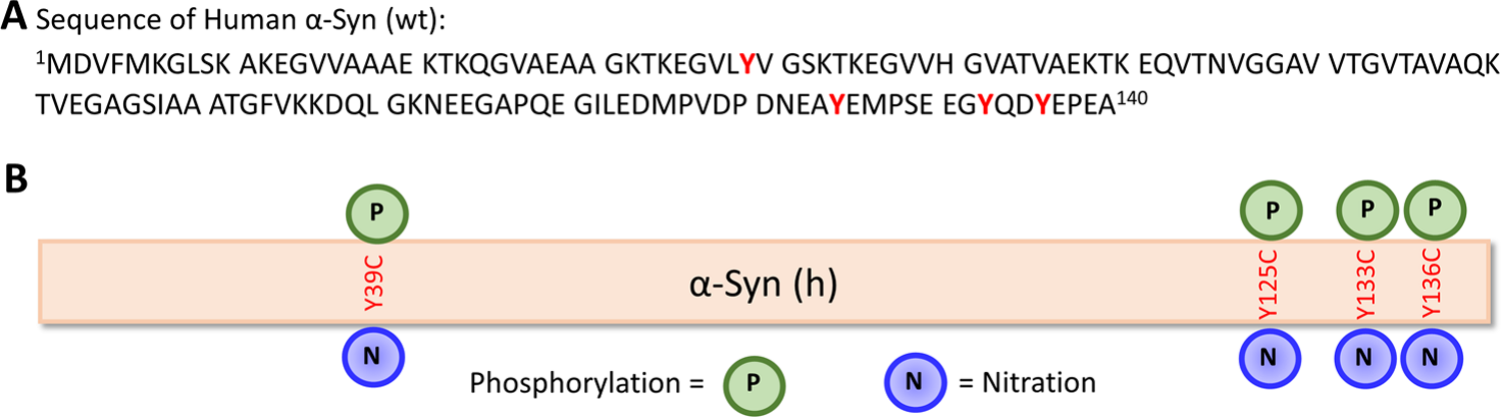
(A) Amino acid sequence of the wild-type (wt) α-Syn (h): the four Tyr residues are highlighted in red. (B) Schematic representation of the α-Syn phosphorylation and nitration at Tyr → Cys mutation points.

α-Syn is a highly soluble, acidic protein expressed in the neurons of the central and peripheral nervous system^25^; it belongs to the family of intrinsically disordered proteins.^26^ The aggregation and fibrillogenesis of α-Syn in the brain are the defining features of PD and other synucleinopathies.^27^ Currently, α-Syn is one of the most sought targets for developing diagnostic and therapeutic strategies; moreover, increasing evidence indicates that PTMs are key regulators of its pathogenic properties.^28^ Human α-Syn has four Tyr residues: one is in the N-terminal region at position 39 and the other three are in the C-terminal region at positions 125, 133, and 136 (Figure 4A). Each of these residues is susceptible to competing PTMs such as phosphorylation and nitration (Figure 4B). In particular, phosphorylation at Tyr-39, Tyr-125, Tyr-133, and Tyr-136 has been detected in postmortem brain tissues from patients with PD and other neurodegenerative diseases.^29^ Similarly, nitration of Tyr residues is one of the key features of pathological aggregates found in PD brains.^30^ Moreover, the accumulation of 3-nitrotyrosine and cross-linked dityrosine α-Syn aggregates has been detected in the brains of patients with PD, dementia with LBs (DLBs), and multiple system atrophy (MSA).^31^ Finally, the link between oxidative and nitrative stress, as well as α-Syn aggregation and pathology formation in synucleinopathies, is also supported by a 9-fold increase in α-Syn nitration at Tyr-39 upon the overexpression of monoamine oxidase B in animal models of PD.^32^ Altogether, these findings underscore the critical importance of deciphering the role of these Tyr modifications in regulating α-Syn aggregation and pathology formation.

Initially, we sought to determine whether mutating Tyr to Cys (Y → C) at 39, 125, 133, or 136 influences the ability of α-Syn to aggregate and form fibrils. All four α-Syn variants (**α-SynY39C**, **α-SynY125C**, **α-SynY133C**, and **α-SynY136C**) and **WT α-Syn** were expressed and purified as described previously.^33^ The purity of the proteins was verified by UPLC-MS (Supporting Information, Figures S23–S26) and SDS-PAGE analyses (Supporting Information, Figure S27). To investigate how a single Y → C mutation at different positions (Tyr-39/125/133/136) affects the conformation of α-Syn monomers, we compared the circular dichroism (CD) spectra of purified **WT α-Syn** and Cys-mutated α-Syn from *Escherichia coli*. **WT-α-Syn** and the four Cys mutants displayed indistinguishable CD spectra, with a minimum at ∼198 nm, which is consistent with a predominantly disordered conformation (Supporting Information, Figure S28A). Next, we investigated the effect of these mutations on the aggregation properties of α-Syn using the well-established thioflavin T (ThT)-based fluorescence assay under the standard conditions used to induce α-Syn aggregates into β-sheet-rich fibrils (incubation of monomeric α-Syn in sterile PBS buffer, pH ∼ 7 at 37 °C with vigorous shaking for 5 days), which are ThT-positive.^34^ The aggregation of all Cys mutants was monitored using the ThT fibrillization assay along with **WT-α-Syn** as a positive control at a 20 μM concentration. All four C → Y variants formed predominantly fibrillar structures that were very similar in morphology, length, and width to **WT-α-Syn** (Supporting Information, Figure S29C–G). Analysis of the kinetic curves from three independent experiments revealed that the **α-SynY125C**, **α-SynY133C,** and **α-SynY136C** mutants exhibited slightly faster aggregation compared with **WT-α-Syn** and **α-SynY39C** (Supporting Information, Figure S29A). After 5 days of incubation, nearly all four proteins (>80–90%) converted to insoluble fibrils, as determined using the sedimentation assay,^33^ i.e., by quantifying the remaining soluble α-Syn species at the end of the aggregation on day 5 (Supporting Information, Figure S29B). The Cys mutants exhibited similar (**α-SynY39C**) or slightly lower (**α-SynY125C**, **α-SynY133C**, and **α-SynY136C**) percentages of soluble proteins post aggregation (Supporting Information, Figure S29B), despite the differences in ThT intensity at the steady state between **α-SynY39C** and the other Y → C mutants. These observations indicate that the Y → C mutations do not markedly change the extent of α-Syn fibrillization or alter the morphology of the final fibrillar structures.

**Figure 5:**
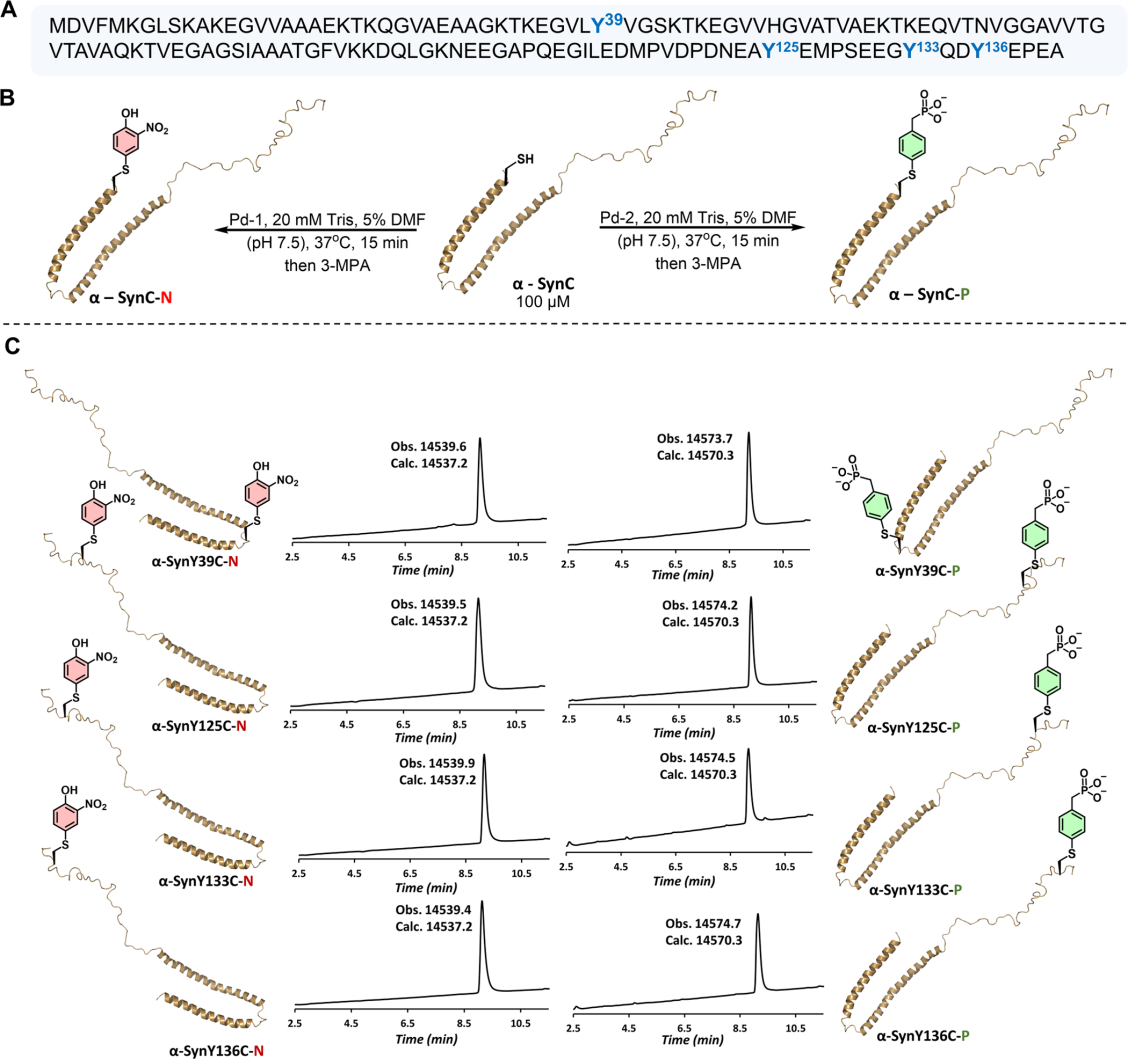
Site-specific incorporation of phosphorylation and nitration analogs to α-Syn protein. (A) Sequence of α-Syn; highlighted Tyr phosphorylation and nitration sites. (B) Schematic representation of the production of nitrated and phosphorylated α-Syn analogs. (C) LCMS chromatograms and the deconvoluted mass spectra of the isolated modified α-Syn analogs. LCMS was performed using 0.1% formic acid in water and acetonitrile as the mobile phases. Modified proteins were obtained in the protonated form.

Next, we used the same approach described above to produce homogeneously phosphorylated or nitrated α-Syn with high fidelity. We initially treated **α-SynY39C** with **Pd-2** using our optimized reaction conditions in 20 mM Tris buffer and 5% DMF (pH 7.5) at 37 °C for 15 min. This provided the desired monophosphorylated product **α-SynY39C-P** in 73% isolated yield after RP-HPLC purification (see the Supporting Information, Section 9.1). Reacting the other analogs **α-SynY125C**, **α-SynY133C**, and **α-SynY136C** separately with **Pd-2** under the same reaction conditions afforded the desired monophosphorylated products **α-SynY125C-P, α-SynY133C-P,** and **α-SynY136C-P** in 83, 79, and 77% isolated yields, respectively. The identity and purity of all phosphorylated analogs were confirmed by LCMS (Figure 5), SDS-PAGE analysis (Supporting Information, Figure S27), and ^31^P NMR (Supporting Information, Figures S43–S46). Subsequently, we reacted the **α-SynY39C**, **α-SynY125C**, **α-SynY133C**, and **α-SynY136C** analogs separately with **Pd-1**, which provided the desired mononitrated products **α-SynY39C-N**, **α-SynY125C-N**, **α-SynY133C-N**, and **α-SynY136C-N** in 43, 45, 55, and 30% isolated yields, respectively (see the Supporting Information, Section 9.2). The identity and purity of the nitrated analogs were confirmed by LCMS (Figure 5) and SDS-PAGE analysis (Supporting Information, Figure S27). Remarkably, this approach enabled the facile insertion of the nitration and phosphorylation analogs at all possible nitration and phosphorylation sites on a milligram scale (Figure 5). Notably, previous reports have demonstrated the potential of semisynthetic strategies to prepare nitrated and phosphorylated α-Syn at Tyr-39 and Tyr-125 residues.^35^ These proteins were isolated in moderate to good yields due to the multistep operation, which required multiple synthetic and isolation steps. On the other hand, the preparation of nitrated and phosphorylated α-Syn at Tyr-133 and Tyr-136 has never been reported. Importantly, our late-stage approach provided the nitrated and phosphorylated α-Syn analogs at all Tyr sites in a single-step operation in good yields. Such success in building a focused library of site-specifically nitrated and phosphorylated α-Syn analogs further reinforces the power of the palladium-mediated late-stage arylation for the rapid production of homogeneous PTM-modified recombinant proteins in high quality and good quantities.

### Phosphorylation at Residue 39 or 125 Significantly Inhibits α-Syn Aggregation In Vitro

**Figure 6:**
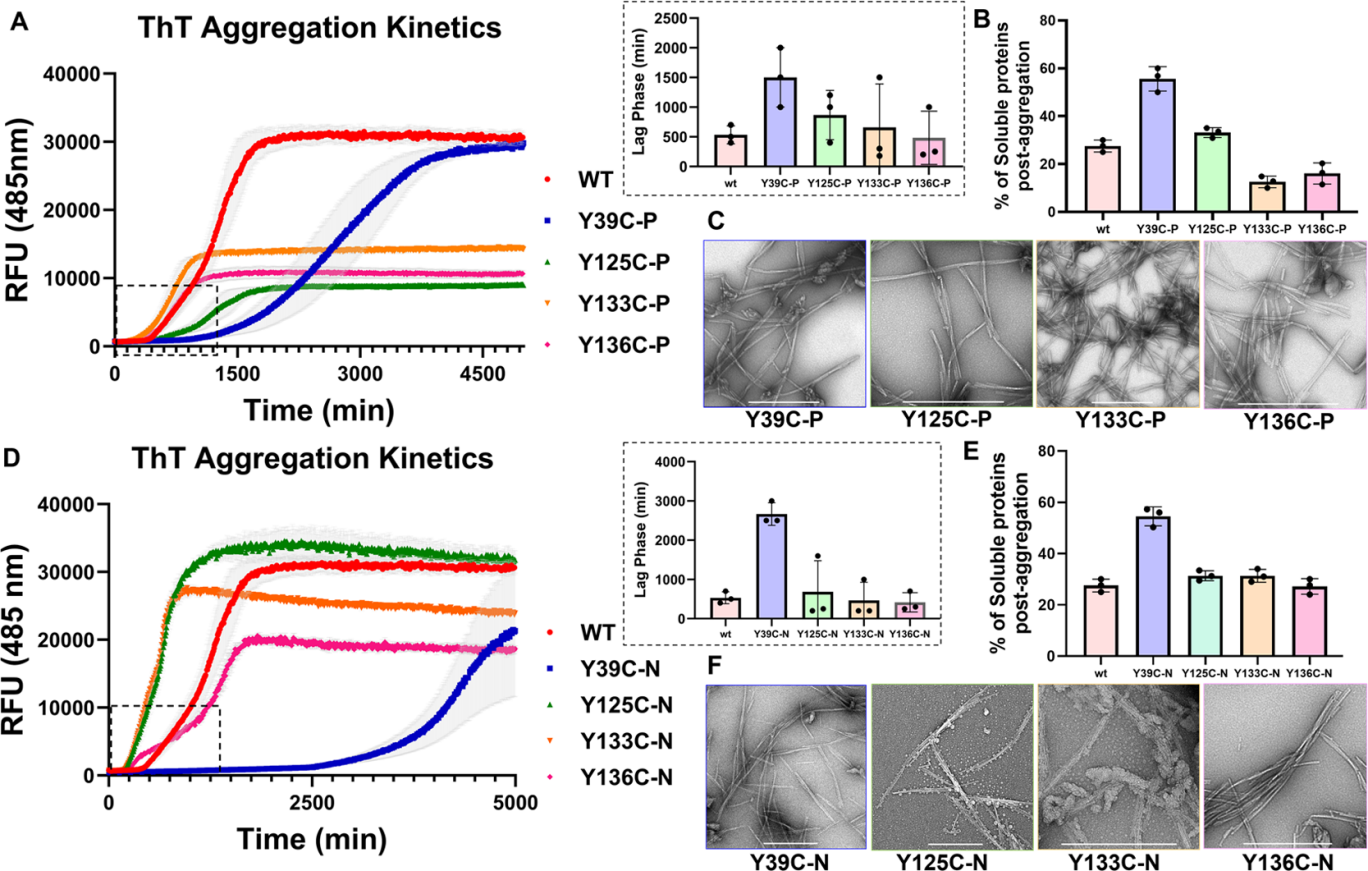
(A) Aggregation kinetics of phosphorylated α-Syn based on thioflavin T fluorescence (ThT ± SEM; *n* = 3). The difference in the lag phase is shown in the bottom box. (B) Corresponding quantification of the remaining soluble protein ± SEM of **WT α-Syn**, **α-SynY39C-P**, **α-SynY125C-P**, **α-SynY133C-P**, and **α-SynY136C-P**. (C) Electron micrographs of phosphorylated proteins (the scale bars are 500 nm). (D) Aggregation kinetics of nitrated α-Syn based on thioflavin T fluorescence (ThT ± SEM, *n* = 3). (E) Corresponding quantification of the remaining soluble protein ± SEM of **WT α-Syn**, **α-SynY39C-N**, **α-SynY125C-N**, **α-SynY133C-N**, and **α-SynY136C-N**. (F) Electron micrographs of nitrated proteins (the scale bars are 500 nm). For expanded EM micrographs, please see the Supporting Information, Section 17.

All phosphorylated α-Syn proteins showed CD spectra similar to that of **WT α-Syn** (Supporting Information, Figure S28B). A comparison of the aggregation profile of all the phosphorylated analogs shows that **α-SynY133C-P** and **α-SynY136C-P** exhibited a lag time similar to the **WT α-Syn** protein but showed a noticeable reduction in ThT intensity at the steady state (Figure 6A). Interestingly, **α-SynY125C-P** and **α-SynY39C-P** proteins aggregated slower than did the **WT α-Syn** protein, along with a longer lag phase and a reduction in aggregation observed for **α-SynY39C-P** (Figure 6B). The trend of the lag phase for these phosphorylated analogs is **α-SynY39C-P** > **α-SynY125C-P** > **WT α-Syn** ∼ **α-SynY136C-P** ∼ **α-SynY133C-P**. Interestingly, the ThT intensity of the **α-SynY125C-P**, **α-SynY136C-P**, and **α-SynY133C-P** proteins at the steady state was significantly lower than that of **WT α-Syn** or **α-SynY39C-P**. We hypothesized that this could be due to the presence of reduced levels of fibrils or the formation of fibrils that exhibit reduced affinity for ThT.^36^ To more accurately assess the extent of fibrillization, we quantified the amounts of the remaining monomers after 5 days using the sedimentation assay (Supporting Information, Figure S31F–I). Interestingly, we observed complete fibrillization for all **α-SynY125C-P**, **α-SynY133C-P**, and **α-SynY136C-P** proteins. However, the percentage of soluble protein for **α-SynY39C-P** was higher than those for the remaining proteins. These results suggest that lower plateau values for **α-SynY125C-P**, **α-SynY133C-P**, and **α-SynY136C-P** analogs could be attributed to a reduction in the ThT binding site due to differences in the fibril structure or the lateral association of the phosphorylated fibrils. Consistent with this hypothesis, the fibrils formed by the phosphorylated analogs appeared morphologically wider (Figure 6C), 2–3-fold wider than the **WT α-Syn** protein (Supporting Information, Figure S33).

Our results on the marked reduction in the aggregation propensity of **α-SynY39C-P** are consistent with previous observations using semisynthetic pY39-α-Syn.^35a^ Interestingly, the delayed aggregation kinetics of **α-SynY125C-P** differ from the previously reported semisynthetic α-SynY125, where phosphorylation at this site was shown not to significantly influence the conformation or the aggregation kinetics of α-Syn.^35d^ Importantly, our work represents the first study on the effect of the site-specific phosphorylation of α-Syn at Tyr-133 and Tyr-136 (**α-SynY133C-P** and **α-SynY136C-P**) on the aggregation kinetics and properties of α-Syn. We showed that phosphorylation at these residues does not influence the aggregation kinetics of the protein but results in the formation of morphologically wider fibrils compared with those of **WT α-Syn**.

### N-Terminal but Not C-Terminal Nitration Inhibits the Aggregation of α-Syn In Vitro

Next, we investigated the effect of nitration on the conformation and in vitro fibrillization of α-Syn. Nitration at all Tyr residues did not induce any changes in the CD spectra of α-Syn (Supporting Information, Figure S28C). Similar to what we observed with phosphorylation, only nitration at Tyr-39 resulted in a significant delay in the aggregation of α-Syn, whereas nitration at the C-terminal Tyr residues resulted in either similar (Tyr-125) or slightly faster aggregation (Tyr-133 and Tyr-136) (Figure 6D). Even after 5 days of aggregation, the percentage of the remaining soluble protein in the case of **α-SynY39C-N** is higher than those of the remaining nitrated proteins (**α-SynY125C-N**, **α-SynY133C-N**, and **α-SynY136C-N**) (Figure 6E). Next, we evaluated the structural properties of the aggregates formed after 5 days by TEM. Interestingly, the nitrated analogs of α-Syn form fibrils with distinct morphologies compared with their native Tyr-nitrated analogs (Figure 6F). **α-SynY39C-N** and **α-SynY125C-N** both formed shorter fibrils after longer aggregation times.^35e^ As previously reported, both native nY39-α-Syn and nY125-α-Syn formed predominantly short and thick fibrils, particularly with nY39-α-Syn. The nY125-α-Syn aggregates exhibited a high propensity to stack in parallel.^35e^ In contrast, both **α-SynY39C-N** and **α-SynY125C-N** showed elongated fibrils similar to **WT α-Syn** or their modified analogs (Figure 6F). Although our results on the **α-SynY39C-N** analog are consistent with the previous finding from our group, demonstrating a strong aggregation inhibitory effect of nitration at this position,^35e^ the results on **α-SynY125C-N** show a different trend than the previously reported results, where we observed increased aggregation kinetics for nY125-α-Syn (Figure 6D).^35e^

**Figure 7:**
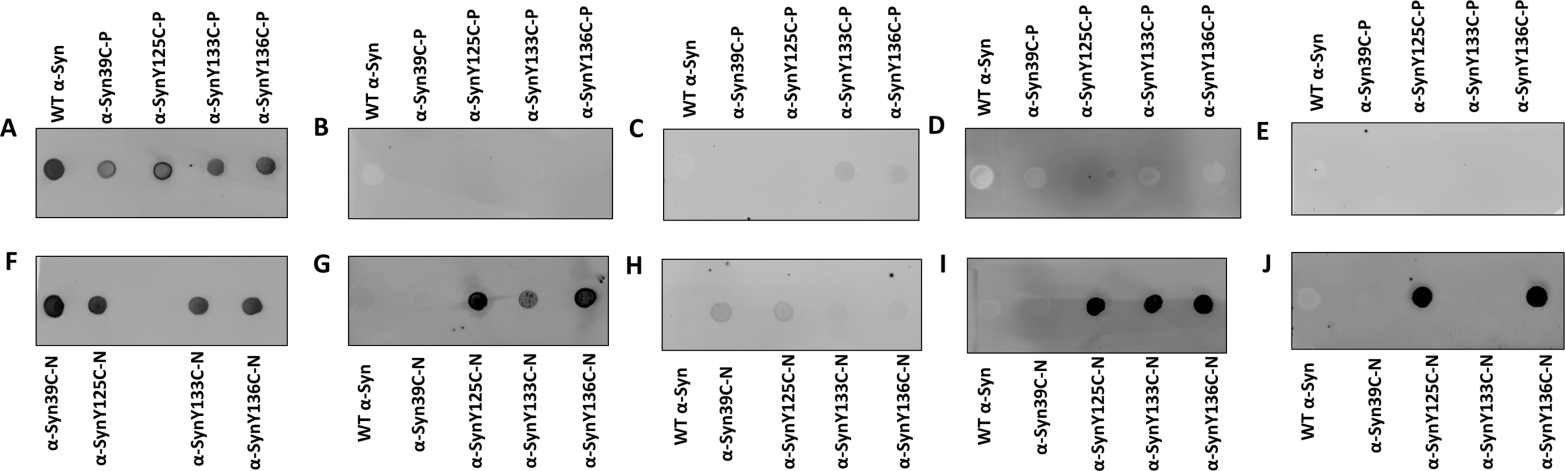
Dot-blot analysis of the nitrated and phosphorylated α-Syn(h) analogs against (A, F) anti-α-Syn(H) antibody (epitope: Syn 91-99), (B) anti-Syn-pY39, (C) anti-Syn-pY125, (D) anti-Syn-pY133, (E) anti-Syn-pY133, Syn-pY136, (G) anti-Nitrated-Syn, (H) anti-Syn-nY39, (I) anti-Syn-nY125, nY133, and (J) anti-Syn-nY125, nY136.

α-Syn fibrils produced in vitro are commonly used to induce the aggregation and inclusion formation of endogenous α-Syn proteins in cellular and animal models of α-Syn pathology formation. Recent studies from the Lashuel Laboratory and others have also employed posttranslationally modified α-Syn fibrils to dissect the role of PTMs in regulating α-Syn aggregation and pathogenicity. The internalization and fate of these modified fibrils are monitored primarily by using PTM-specific antibodies. Therefore, to determine whether our phospho- and nitromimetics could be detected by antibodies that recognize the bona fide phosphorylated and nitrated α-Syn, we performed dot-blot analysis using commercially available antibodies specific to 39/125/133/136 native phosphorylated or nitrated α-Syn(h).^29a^ As shown in Figure 7, the nitrated proteins were recognized by the corresponding antibodies (Figure 7G–J and Figure S12). In contrast, the phosphorylated analogs were not recognized by the phospho-antibodies (Figure 7B–E and Figure S11). We believe that this behavior can be attributed to the substitution of the oxygen atom in the phosphorylation by methylene. Although these findings preclude the use of pS129 antibodies to track the modified fibrils prepared using the phospho- and nitromimetics, they present an opportunity to address one of the major challenges faced by the community today, which is how to distinguish between exogenous modified fibrils and newly formed fibrils in cells with the same modifications. Therefore, the phosphorylated proteins that we produced here represent important tools that enable selective monitoring, evolution, and clearance of only newly seeded fibrils in the cells. Importantly, the fact that all the antibodies against nitrated α-Syn recognized the nitrated α-Syn analogs supports the conclusion that the nitration method described here could serve as a general method to study Tyr-nitration and could provide a rapid and efficient method for generating modified α-Syn protein standards and tools for biomarker discovery and validation.

One of the limitations of our approach is the introduction of single-atom alteration and insertion of the (−S−) atom at the desired PTM site. Although previous Cys alkylation methods used to install aliphatic modifications (e.g., methylation) through thioether linkage have shown that such an alteration is a reasonable mimic of native PTMs,^15^ systematic studies on the impact of such alterations have not been performed. Our work on α-Syn presents the first opportunity for such a comparison since the corresponding site-specifically modified bona fide phosphorylated and nitrated forms of this protein at Tyr-39 and Tyr-125 have been produced and characterized.^35d,37^ Our data show that both bona fide phosphorylated and nitrated proteins at Tyr-39 exhibited similar aggregation properties, whereas different results were observed on α-Syn aggregation kinetics when these modifications were introduced at the C-terminus of the protein. These observations could be explained by the fact that Tyr-39 occurs within a more hydrophobic domain of the α-Syn sequence and is part of the structured amyloid core of the fibrils,^38^ whereas Tyr-125 occurs in a highly negatively charged and disordered domain that lies outside the amyloid core and is sensitive to pH and interactions with different types of ions and metals.

## Conclusions

The site-specific installation of aromatic PTMs into recombinant protein provides a versatile approach to produce homogeneous analogs with novel aromatic PTMs, e.g., in Tyr residue. We developed a rapid and effective strategy that enables the site-selective installation of essential aromatic PTMs into native peptides and proteins containing an engineered Cys site that directs site-specific installation. The reaction proceeds within minutes through a palladium(II)-mediated S–C(sp^2^) bond formation process under ambient conditions. We demonstrated that this strategy could be used to insert novel PTMs such as nitration and phosphorylation mimics into several protein targets in high quality and good yields, including the Myc and Max TFs and α-Syn on a multimilligram scale. The ability to produce a spectrum of site-specifically modified α-Syn analogs (at Tyr-39, Tyr-125, Tyr-133, and Tyr-136) enabled for the first time the investigation and comparison of the effect of nitration and phosphorylation on all Tyr residues in modulating α-Syn aggregation properties in vitro. Furthermore, this is the first study to investigate the effect of competing site-specific nitration or phosphorylation at Tyr-133 and Tyr-136. Importantly, this strategy leverages the power of organometallic palladium chemistry to functionalize proteins with aromatic PTMs. Recently, elegant strategies involving novel organometallic gold reagents and activated pyridinium salts have been reported for rapid Cys arylation, with enhanced reaction rates (10^4^ to 10^5^ M^–1^ s^–1^).^18d,18e^ These approaches are envisioned to further expand the scope of accessible aromatic transformations to functionalize complex biomolecules for various applications.

The ability to introduce late-stage, postfolding, or postaggregation PTMs, along with the efficiency of the reactions and good yields, is envisioned to pave the way for future studies to explore the rarely explored role of aromatic PTM sites and their cross-talk with other PTMs in regulating the function, dysfunction, or pathogenicity of proteins, for example, investigating the effect of postfibrillization site-specific nitration or phosphorylation of α-Syn and its impact on α-Syn fibril uptake, processing, seeding activity, and pathological spreading in the brain and peripheral tissues. Finally, this method could be further extended to transfer other native and non-native aromatic PTMs, e.g., Tyr-sulfonation, for various biochemical and biomedical studies. This and other research programs are currently under investigation in our laboratories.

## Supporting Information Available

Synthesis and methods, as well as biochemical and biophysical characterization of all compounds (PDF)
