# Supplementary material for: A Versatile Method for Site-Specific Chemical Installation of Aromatic Posttranslational Modification Analogs into Proteins
Source: J Am Chem Soc. 2024 Sep 3;146(37):25788–98. doi: 10.1021/jacs.4c08416 (PMC11421021; doi:10.1021/jacs.4c08416)
Supplement: Supplementary file 1 — ja4c08416_si_001.pdf [file ja4c08416_si_001.pdf]

Supporting Information

A Versatile Method for Site-Specific Chemical Installation of Aromatic Posttranslational Modification Analogs into Proteins

Xiaoxi Lin,[1] Shaswati Mandal,[2] Raj V. Nithun,[1] Rajasekhar Kolla,[2] Bouchra Bouri,[3] Hilal A. Lashuel,[2]* and Muhammad Jbara[1]*

[1]School of Chemistry, Raymond and Beverly Sackler Faculty of Exact Sciences, Tel Aviv University, Tel Aviv, 69978 Israel

[2]Laboratory of Molecular and Chemical Biology of Neurodegeneration, Institute of Bioengineering, School of Life Sciences, École Polytechnique Fédérale de Lausanne, Lausanne, Switzerland

[3]Protein Production and Structure core facility, School of Life Sciences, École Polytechnique Fédérale de Lausanne, CH-1015, Lausanne, Switzerland

*Correspondence to: jbaram@tauex.tau.ac.il, hilal.lashuel@epfl.ch

Table of Contents

1. EXPERIMENTAL 4

1.1 MATERIALS 4
1.2 ANALYTICAL HPLC ANALYSIS 4
1.3 LIQUID-CHROMATOGRAPHY MASS SPECTROMETRY (LC-MS) ANALYSIS 5
1.4 UHPLCMS ANALYSIS 5
1.5 PREPARATIVE RP-HPLC PURIFICATION 5

2. SYNTHESIS OF PALLADIUM(II) OXIDATIVE ADDITION COMPLEXES (PD-1 AND PD-2) 6

3. PROTEIN SEQUENCES 8

4. CHEMICAL SYNTHESIS OF PEPTIDE FRAGMENTS 9

4.1 SYNTHESIS OF MODEL PEPTIDE (P1) 9
4.2 SYNTHESIS OF CYS-MAX(62-102) FRAGMENT (1) 10
4.3 SYNTHESIS OF MAX(22-60)-NBZ FRAGMENT (2) 11
4.4 SYNTHESIS OF CYS-MYC(400-434) FRAGMENT (3) 13
4.5 SYNTHESIS OF MYC(353-398)-NBZ FRAGMENT (4) 14
5.1 CHEMICAL SYNTHESIS OF MAXA61C ANALOG 16
5.2 CHEMICAL SYNTHESIS OF MYCA399C ANALOG 17

6. SITE-SPECIFIC INSTALLATION OF NITRATION AND PHOSPHORYLATION ANALOGS 18

6.1 NITRATION OF MODEL PEPTIDE P1 WITH PD-1 18
6.2 PHOSPHORYLATION OF MODEL PEPTIDE P1 WITH PD-2 19

7. SITE-SPECIFIC PHOSPHORYLATION AND NITRATION OF MODEL PROTEINS 20

7.1 SITE-SPECIFIC PHOSPHORYLATION OF MAXA61C WITH PD-2 20

7.2 SITE-SPECIFIC NITRATION OF MAXA61C WITH PD-1 20

7.3 SITE-SPECIFIC PHOSPHORYLATION OF MYCA399C WITH PD-2 22

7.4 SITE-SPECIFIC NITRATION OF MYCA399C WITH PD-1 22

8. ISOLATION OF MODIFIED MAXA61C ANALOGS AND STABILITY ANALYSIS 24

8.1 ISOLATION OF PHOSPHORYLATED MAXA61C 24

8.2 ISOLATION OF NITRATED MAXA61C 25

8.3 STABILITY OF ISOLATED MAXA61C-P 26

8.4 STABILITY OF ISOLATED MAXA61C-N 26

9. SITE-SPECIFIC A-SYN PHOSPHORYLATION AND NITRATION 27

9.1 LATE-STAGE A-SYN PHOSPHORYLATION 27

9.1.1 A-SYNY39C PHOSPHORYLATION WITH PD-2 27

9.1.2 A-SYNY125C PHOSPHORYLATION WITH PD-2 29

9.1.3 A-SYNY133C PHOSPHORYLATION WITH PD-2 30

9.1.4 A-SYNY136C PHOSPHORYLATION WITH PD-2 31
9.2 LATE-STAGE A-SYN NITRATION 32
9.2.1 A-SYNY39C NITRATION WITH PD-1 32
9.2.2 A-SYNY125C NITRATION WITH PD-1 34
9.2.3 A-SYNY133C NITRATION WITH PD-1 35
9.2.4 A-SYNY136C NITRATION WITH PD-1 36
10. PREPARATION & CHARACTERIZATION OF RECOMBINANT MONOMERIC A-SYN SPECIES . 37
11. SDS-PAGE ANALYSIS 40
12. CIRCULAR DICHROISM SPECTROSCOPY 41
13. TRANSMISSION ELECTRON MICROSCOPY (TEM) 41
14. THT AGGREGATION KINETICS ASSAY 42
15. SEDIMENTATION ASSAY 44
16. NMR SPECTRA OF PD-1 AND PD-2 50
17. TEM MICROGRAPHS 58
18. CRYSTAL DATA AND STRUCTURE REFINEMENT FOR PD-2 71
19. REFERENCES 72

1. Experimental

1.1 Materials

Fmoc-L-Phe-OH, Fmoc-L-Asn(Trt)-OH, Fmoc-L-Gln(Trt)-OH, Fmoc-L-Arg(Pbf)-OH, Fmoc-L-Tyr(*t*Bu)-OH, Fmoc-L-Glu(O*t*Bu)-OH, Fmoc-L-Ala-OH, Fmoc-L-Leu-OH, Fmoc-L-His(Trt)-OH, Fmoc-L-Asp(O*t*Bu)-OH, Fmoc-L-Pro-OH, Fmoc-L-Cys(Trt)-OH, Fmoc-L-Lys(Boc)-OH, Fmoc-L-Lys(Alloc)-OH, Fmoc-L-Ile-OH, Fmoc-L-Ser(*t*Bu)-OH, Fmoc-Gly-OH, Fmoc-L-Nle-OH, Fmoc-L-Thr(*t*Bu)-OH, Boc-L-Ala-OH, Boc-L-asparagine, 1,3-Diisopropylcarbodiimide (DIC), 4-Bromobenzylphosphonic acid were purchased from Sigma-Aldrich. Fmoc-L-His(Boc)-OH was purchased from CEM. 1-[Bis(dimethylamino)methylene]-1H-1,2,3-triazolo[4,5-b] pyridinium 3-oxide hexafluorophosphate (HATU), (2-(1H-benzotriazol-1-yl)-1,1,3,3-tetramethyluronium hexafluorophosphate (HBTU), and HOBt HYDRATE were purchased from Luxembourg Bio Technologies Ltd. Rink Amide ProTide resin was obtained from CEM. 2-Dicyclohexylphosphino-2′,6′-diisopropoxybiphenyl (RuPhos) was purchased from Angene chemical private limited. 4-Bromo-2-nitrophenol (98%) was purchased from thermos scientific. Diethyl ether (Et_2O, 99.8% stabilized, ACS grade) was obtained from MACRON. Dichloromethane (CH_2Cl_2, ≥99.5% stabilized with 50 ppm Amylene) was obtained from CHEM-LAB. *N, N*-dimethylformamide (DMF, Peptide Synthesis-grade) and acetonitrile (ACN, LC/MS Grade) were purchased from J.T.Baker. Trifluoroacetic acid (TFA, ≥99% ReagentPlus®), diisopropylethylamine (DIEA, ≥99% ReagentPlus®), piperidine (≥99% ReagentPlus®), triisopropylsilane (TIS, 98%), formic acid (FA, 98-100% for LC/MS), n-Pentane (AR) were purchased from Bio-Lab ltd. Water for all reactions carried out on proteins and for reverse-phase purification was obtained via filtration of deionized water through a MilliporeSigmaTM Milli-Q™ Ultrapure Water System. All chemicals obtained from supplier were used as received without further purification.

1.2 Analytical HPLC analysis

Analytical RP-HPLC chromatograms were acquired using Thermo Scientific Vanquish HPLC. Mobile phases used are solvent A (0.05% TFA in water) and solvent B (0.05% TFA in acetonitrile).

XBridge® BEH 3.5 µm-C4 300 Å Column (150 x 4.6 mm); LC conditions: 5% B from 0–1.0 min, then a linear gradient from 5% to 60% B from 1.0–28.0 min, 1.0 mL/min flow rate. Mass spectra were acquired using Thermo Scientific ISQ EM Mass spectrometer.

1.3 Liquid-chromatography mass spectrometry (LC-MS) analysis

LC chromatograms were acquired using Thermo Scientific Vanquish HPLC. Mobile phases used are solvent A (0.1% FA in water) and solvent B (0.1% FA in acetonitrile).

BioZen™ 2.6 µm-C4 Widepore LC column (150 x 2.1 mm); LC conditions: 5% B from 0–1.0 min, then a linear gradient from 5% to 50% B from 1.0–11.0 min, 0.3 mL/min flow rate. Mass spectra were acquired using Thermo Scientific ISQ EM Mass spectrometer.

1.4 UHPLCMS analysis

Before analysis, proteins were desalted online by reversed-phase chromatography on a Poroshell 300SB C3 column (1.0 x 75 mm, 5 um, Agilent Technologies, Santa Clara, CA, on the LTQ system). Flow rate of 0.3 ml/min and were eluted from 5% to 95% of solvent B against solvent A, linear gradient. Mass spectrometry analysis was performed on the LTQ system (Thermo Scientific, San Jose, CA). The solvent composition was, solvent A: 0.1% formic acid in ultra-pure water; solvent B: 0.1% formic acid in acetonitrile. MagTran software (Amgen Inc., Thousand Oaks, CA) was used for charge state deconvolution and MS analysis.

1.5 Preparative RP-HPLC purification

Preparative RP-HPLC was performed using Thermo Scientific DIONEX UltiMate 3000 Variable Wavelength Detector, equipped with a column of choice. Mobile phases used for LC analysis were solvent A (0.05% TFA in water), and solvent B (0.05% TFA in acetonitrile). The following LC methods were used:

Method A: Jupiter® 5 µm C4 300 Å LC column (250 x 10 mm), LC conditions: 5% B from 0–5.0 min, then linear gradient from 5% to 60% B from 5-65 min, 4 mL/min flow rate at 30 ^{o}C.

Method B: Jupiter® 5 µm C18 300 Å LC column (250 x 10 mm), LC conditions: 5% B from 0–5.0 min, then linear gradient from 5% to 60% B from 5-65 min, 4 mL/min flow rate at 30 ^{o}C.

Method C: XBridge® Protein BEH C4 OBD™ Prep Column, 300 Å, 5 µm, 250 mm x 10 mm, 1/pkg, LC conditions: 5% B from 0–5.0 min, then linear gradient from 5% to 60% B from 5-65 min, 4 mL/min flow rate at 30 ^{o}C.

2. Synthesis of palladium(II) oxidative addition complexes (Pd-1 and Pd-2)

Synthesis of Pd-1:

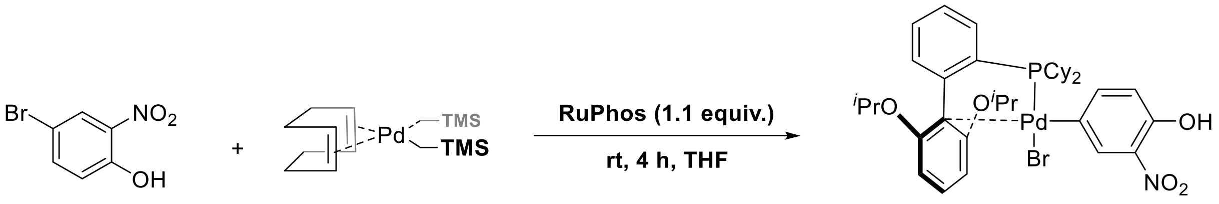

1.1 equiv. 1.0 equiv. **Pd-1**

In a 50 mL nitrogen-filled Schlenk flask equipped with a magnetic stir bar, RuPhos (51.4 mg, 0.11 mmol, 1.1 equiv.) and 4-Bromo-2-nitrophenol (24.1 mg, 0.11 mmol, 1.1 equiv.) were dissolved in 1 mL THF.[1,2] Solid (COD)Pd(CH_2SiMe_3)$_2$ (38.9 mg, 0.1 mmol, 1.0 equiv.) was dissolved in 1 mL THF was added rapidly in one portion. The resulting solution was stirred for 4 h at room temperature. After that, pentane (5 mL) was added, and the resulting mixture was placed into a –20 °C freezer for 3 h. The resulting precipitate was filtered, washed with pentane (3 × 3 mL), and dried under vacuum to afford **Pd-1** as a yellow powder (88% yield).

^{1}H NMR (400 MHz, CD_2Cl_2) δ 10.30 (s, 1H), 7.66 (dd, *J* = 10.6, 7.1 Hz, 3H), 7.50 (t, *J* = 7.5 Hz, 2H), 7.43 (t, *J* = 7.4 Hz, 1H), 6.93 (d, *J* = 8.9 Hz, 1H), 6.86 (dd, *J* = 8.6, 5.7 Hz, 1H), 6.71 (t, *J* = 6.5 Hz, 2H), 4.67 (m, 2H), 2.13 (dd, *J* = 20.9, 10.9 Hz, 2H), 1.82 (s, 5H), 1.71 (d, *J* = 11.2 Hz, 4H), 1.61 (s, 5H), 1.41 (d, *J* = 5.9 Hz, 6H), 1.30 – 1.10 (m, 8H), 1.05 (d, *J* = 5.7 Hz, 6H). ^{31}P NMR (162 MHz, C_6D_6) δ 28.0 (s). ^{13}C NMR (101 MHz, CD_2Cl_2) δ 159.1 (s), 152.3 (d, *J* = 8.2 Hz), 148.0 (s), 147.4 (s), 144.4 (t, *J* = 16.3 Hz), 134.8 (d, *J* = 17.3 Hz), 132.5 (t, *J* = 11.5 Hz), 130.7 (d, *J* = 15.5 Hz), 129. 8 (d, *J* = 5.0 Hz), 129.4 (d, *J* = 5.0 Hz), 127.0 (s), 126.4 (d, *J* = 5.5 Hz), 124.3 (s), 33.56 (s), 28.3 (s), 26.7 (dd, *J* = 25.7, 12.2 Hz), 25.9 (s), 21.8 (s), 21.1 (s). HRMS (APPI) m/z: $[M-H]^-$ calcd. for $C_{36}H_{46}NO_5PBr^{[79]}Pd^{[104]}$: 786.1337, found : 786.1340.

Synthesis of Pd-2:

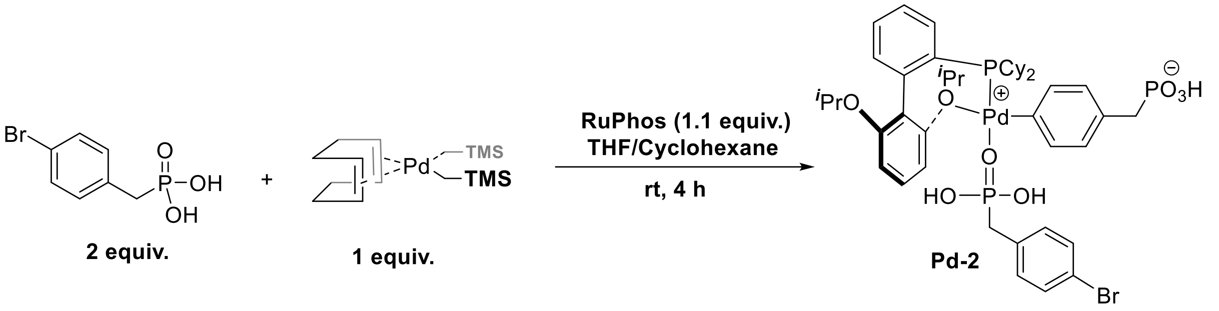

In a 50 mL nitrogen-filled Schlenk flask equipped with a magnetic stir bar, RuPhos (56 mg, 0.12 mmol, 1.1 equiv.) and 4-Bromobenzylphosphonic acid (50 mg, 0.2 mmol, 2.0 equiv.) were dissolved in 1 mL dry THF. Solid (COD)Pd(CH_2SiMe_3)$_2$ (46 mg, 0.12 mmol, 1.0 equiv.) was dissolved in Cyclohexane (1 mL) and added rapidly in one portion. The resulting solution was stirred for 4 h at room temperature. After that, pentane (5 mL) was added, and the resulting mixture was transferred to a vial (20 mL) and placed into a –20 °C freezer for 3 h. The resulting precipitate was collected, washed with pentane (3 × 3 mL), and dried under vacuum to afford **Pd-2** as a light-yellow powder (61% yield).

Note: Pd-2 was obtained with a phosphonate, as indicated by ^{31}P NMR and X-ray structure analysis. The ^{31}P NMR spectrum of Pd-2 showed three different signals at 37.3 ppm, 25.1 ppm, and 21.2 ppm, corresponding to the RuPhos ligand coordinated with palladium, the phosphonate 4-BrC$_6$H$_4$CH$_2$P(O)(OH)$_2$, and the Tyr-phosphorylation mimic -C$_6$H$_4$CH$_2$PO$_3$H, respectively. Of note, the phosphonate bound to Pd-2 might dissociate under the bioconjugation reaction conditions.

^{1}H NMR (400 MHz, CD_2Cl_2) δ 7.63 (t, *J* = 7.3 Hz, 1H), 7.55 – 7.44 (m, 2H), 7.42 (d, *J* = 7.5 Hz, 3H), 7.21 (d, *J* = 6.6 Hz, 2H), 7.07 (d, *J* = 7.1 Hz, 2H), 6.86 (d, *J* = 7.2 Hz, 1H), 6.79 (d, *J* = 6.9 Hz, 2H), 6.60 (d, *J* = 8.4 Hz, 2H), 4.59 (dt, *J* = 11.6, 5.9 Hz, 2H), 2.88 (d, *J* = 22.3 Hz, 2H), 2.54 (d, *J* = 21.0 Hz, 2H), 2.14 (dd, *J* = 22.6, 11.7 Hz, 2H), 1.91 – 1.56 (m, 12H), 1.49 (d, *J* = 5.8 Hz, 6H), 1.33 – 1.10 (m, 7H), 1.06 (d, *J* = 5.9 Hz, 6H), 0.77 (dd, *J* = 26.1, 13.2 Hz, 3H). ^{31}P NMR (162 MHz, CD_2Cl_2) δ 37.32 (s), 25.09 (s), 21.21 (s). ^{13}C NMR (101 MHz, CD_2Cl_2) δ 159.7 (s), 144.9 (d, *J* = 16.7 Hz), 136.8 (s), 135.7 (s), 134.2 (dd, *J* = 64.6, 32.2 Hz), 131.8 (s), 130. 9 (s), 130.8 (s), 128.3 (s), 126.3 (d, *J* = 5.5 Hz), 105.9 (s), 70.9 (s), 34.0 (d, *J* = 30.2 Hz), 27.9 (d, *J* = 24.9 Hz), 27.1 (d, *J* = 13.4 Hz), 26.8 (d, *J* = 11.2 Hz), 26.0 (s), 21.6 (d, *J* = 12.3 Hz). HRMS (ESI) m/z: [M-H]$^-$ calcd. for $C_{44}H_{58}O_8P_3Br^{[79]}Pd^{[104]}$: 989.1490, found : 989.1473.

Note: Pd-1 and Pd-2 were stored at room temperature under a nitrogen atmosphere which gave no diminished reactivity even after one month.

3. Protein sequences

Model peptide (P1):

KLYA**C**SRYL

Max(22-102)A61C:

ADKRAHHNALERKRRDHIKDSFHSLRDSVPSLQGEKASR**C**^{61}QILDKATEYIQYMRRKNHTHQQD IDDLKRQNALLEQQVRAL

The Met residue at position 74 was mutated with the homologous norleucine (Nle) residue to avoid Met oxidation.

Myc(353-434)A399C:

NVKRRTHNVLERQRRNELKRSFFALRDQIPELENNEKAPKVVILKK**C**^{399}TAYILSVQAEEQKLISE EDLLRKRREQLKHKLEQL

α-Synuclein:

MDVFMKGLSKAKEGVVAAAEKTKQGVAEAAGKTKEGVL**Y**$^{\mathbf{39}}$VGSKTKEGVVHGVATVAEKTK EQVTNVGGAVVTGVTAVAQKTVEGAGSIAAATGFVKKDQLGKNEEGAPQEGILEDMPVDPDNE A**Y**$^{\mathbf{125}}$EMPSEEG**Y**$^{\mathbf{133}}$QD**Y**$^{\mathbf{136}}$EPEA

Highlighted nitration and phosphorylation sites (Tyr 39, 125, 133,136)

4. Chemical synthesis of peptide fragments

4.1 Synthesis of model peptide (P1)

The synthesis was carried out according to the following scheme:

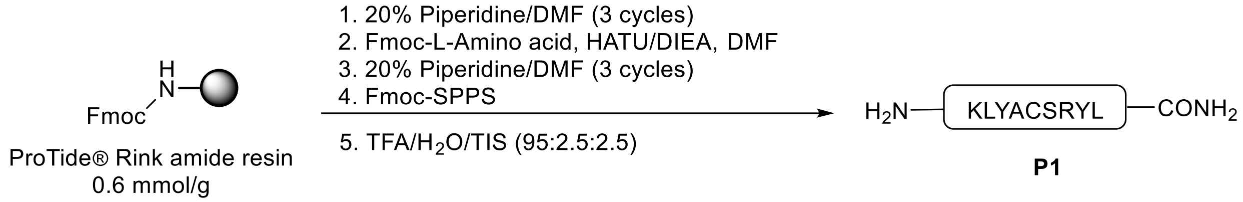

The synthesis of the model peptide **P1** was carried out using Fmoc-SPPS on ProTide® Rink amide resin (166 mg, loading 0.6 mmol/g, 0.1 mmol scale). The resin was pre-swollen with DMF for 30 min in a fritted syringe and then treated with 5 mL 20% piperidine (5 min x 3), followed by washing with DMF (10 mL x 3). The sequence was coupled manually with Fmoc-L-AA-OH (5 equiv.), using HATU (5 equiv.) and 0.25 mL DIEA in 2.5 mL DMF for 20 min at room temperature. After each coupling step, the resin was washed 3 times with DMF and submitted to Fmoc-deprotection using 4 mL 20% piperidine/DMF (containing 0.05 % F.A.) for 1 min, then 4 min, and another 1 min. After coupling all the amino acids, the peptide-bound resin was washed with DMF (5 mL x 3) and DCM (5 mL x 3) and was dried under vacuum. Subsequently, a mixture of TFA/H_2O/TIS (95:2.5:2.5, 8 mL for 0.05 mmol scale) was added to the resin and was shaken for 1.5 h at room temperature. The resin was removed by filtration and washed with TFA (1 mL x 2). To precipitate the peptide, the combined filtrate was added dropwise to cold diethyl ether followed by centrifugation at 4000 rpm for 10 min. Then, the diethyl ether was decanted, followed by dissolution of the peptide with 3 mL 25% acetonitrile/0.1% TFA water and lyophilized to get white powder. The crude dry peptide powder was purified by RP-HPLC (Method A as described in Section 1.5) to provide **P1** as a white powder (59%).

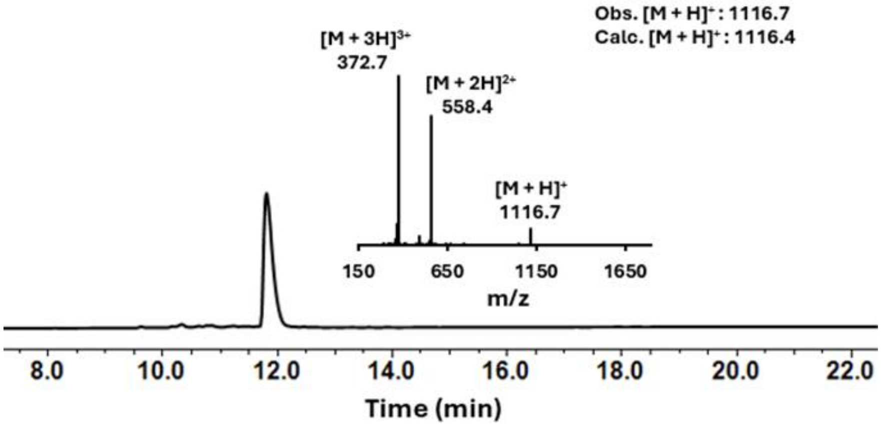

Figure S1. HPLC and MS analysis of model peptide P1. HPLC chromatogram of the UV absorbance at 214 nm and mass-to-charge (m/z) spectrum. HPLC analysis was carried out as described in section 1.2.

4.2 Synthesis of Cys-Max(62-102) fragment (1)

The synthesis was carried out according to the following scheme:

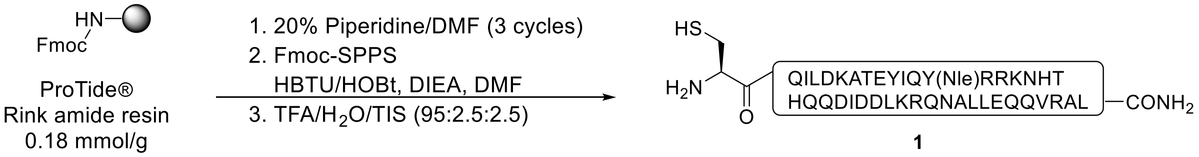

The synthesis of Cys-Max(62-102) fragment (**1**) was carried out using standard Fmoc-SPPS protocol on ProTide Rink amide resin (loading 0.18 mmol/g, 556 mg, 0.1 mmol scale). The resin was pre-swollen in DMF for 30 min in a fritted syringe and then treated with 5 mL 20% piperidine (5 min x 3), followed by washing with DMF (10 mL x 3). The sequence was coupled manually with Fmoc-L-amino acid-OH (5 equiv.) using HBTU/HOBt (5 equiv./5 equiv.) and DIEA (174 µL.) for 45 min at room temperature. For Fmoc-deprotection, the resin was treated with 4 mL 20% piperidine/DMF (contains 0.05% F.A.) for 1 min, then 4 min, and another 1 min. The peptidyl bound resin was washed with DMF (5 mL x 3), DCM (5 mL x 3) and dried under vacuum. Subsequently, a mixture of TFA/H_2O/TIS (95:2.5:2.5, 5 mL for 0.025 mmol scale) was added and shaken for 3 hours at room temperature. The resin was removed by filtration and washed with TFA (1 mL x 2). To precipitate the peptide, the filtrate was added dropwise to cold diethyl ether (35 mL for 0.025 mmol scale) followed by centrifugation (10 min, 4000 rpm). Then, the ether was decanted, followed by the dissolution of the peptide in 3 mL 25% acetonitrile/0.1% TFA water, and lyophilized to get white powder. The crude dry peptide powder was purified by RP-HPLC using Method C (see section 1.5) to provide Cys-Max(62-102) fragment (**1**) as a white powder (14%).

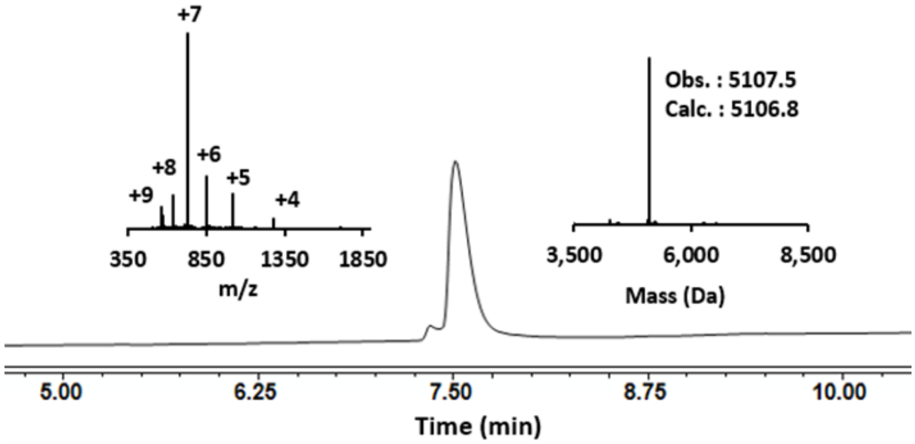

Figure S2. LC-MS analysis of Cys-Max(62-102) fragment (1). LC chromatogram of the UV absorbance at 214 nm, mass-to-charge (m/z) spectrum and deconvoluted mass spectrum. LC-MS analysis was carried out as described in section 1.3.

4.3 Synthesis of Max(22-60)-Nbz fragment (2)

The synthesis was carried out according to the following scheme:

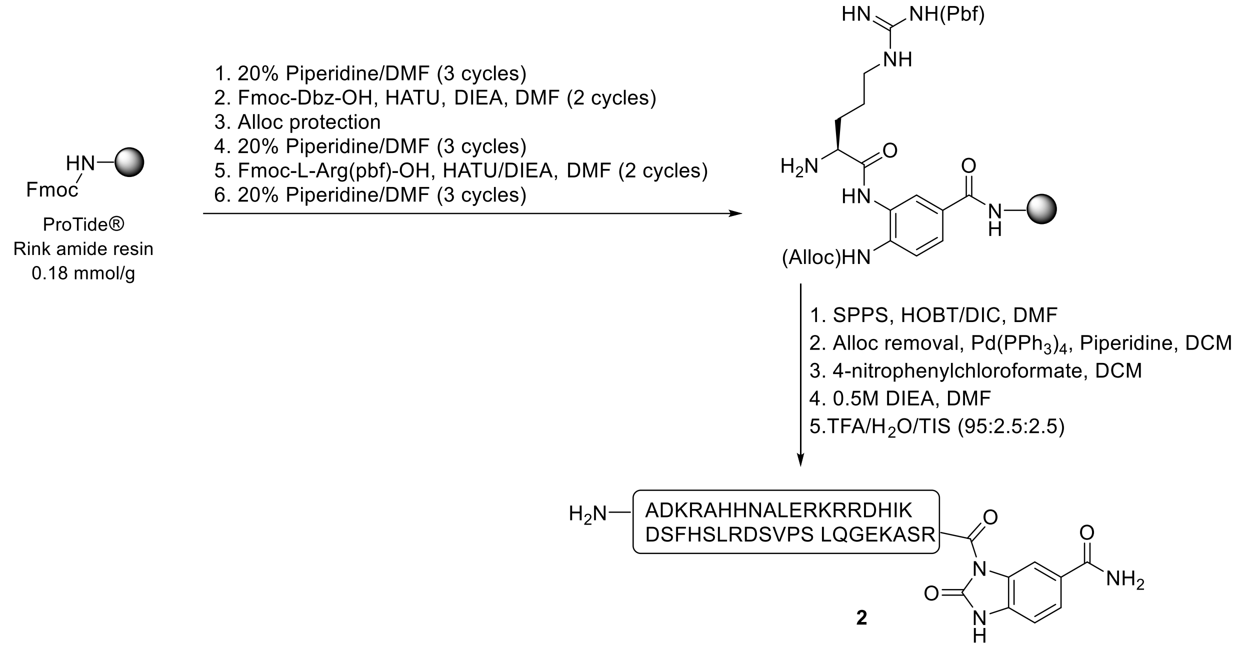

The synthesis of the Max(22-60)-Nbz (**2**) was carried out using standard Fmoc-SPPS protocol on ProTide® Rink amide resin (loading 0.18 mmol/g, 556 mg, 0.1 mmol scale).[3] The resin was pre-swollen in DMF for 30 min in a fritted syringe and then treated with 5 mL 20 % piperidine (contains 0.05 % F.A.) (5 min x 3), followed by washing with DMF (10 mL x 3), and then coupled with Fmoc-Dbz-OH (5 equiv.), using HATU (5 equiv.) and 0.25 mL DIEA for 45 min (2 cycles). Subsequently, the resin was washed with DMF (5 mL x 3) and DCM (5 mL x 3) and was treated with a solution of Allyl chloroformate (50 equiv.) and DIEA (2 equiv.) in 2.5 mL DCM for 12 h at room temperature. For Fmoc removal, the resin was treated with 5 mL 20 % piperidine,0.05 % F.A. (5 min x 3). The first amino acid was coupled manually using Fmoc-L-Arg(pbf)-OH (5 equiv.), using HATU (5 equiv.) and DIEA (250 µL) for 45 min at room temperature (2 cycles). The rest of the sequence was coupled manually with Fmoc-L-AA-OH (5 equiv.), using HOBt (5 equiv.) and DIC (160 µL) for 45 min at room temperature. For Fmoc deprotection, the resin was treated with 20% piperidine in DMF (contains 0.05% F.A.) for 1 min, then 4 min, and another 1 min. The last amino acid was coupled with Boc-L-Ala-OH (5 equiv.) using HOBt (5 equiv.) and DIC (160 µL) for 45 min at room temperature. For Alloc removal, the resin was treated with a solution of piperidine (1.0 mL) and $Pd(PPh_3)_4$ (57.8 mg, 1 equiv.) in 4 mL DCM and was shaken for 50 min at room temperature. Then, the peptidyl bound resin was washed with DMF and DCM before being treated with a solution of 4-nitrophenylchloroformate (5 equiv.) in 1.5 mL DCM for 30 min at 25 °C (3 cycles). The resin was washed with DCM and DMF and treated with a solution of 0.5 M DIEA in DMF and was shaken for 10 min (3

cycles) at room temperature. Then, the resin was washed with DMF (5 mL x 3) and DCM (5 mL x 3) and kept drying under vacuum. Subsequently, a mixture of TFA/H_2O/TIS (95/2.5/2.5, 5 mL for 0.025 mmol) was added to the resin and was shaken for 3 h at room temperature. The resin was removed by filtration and washed with additional TFA (1 mL x 2). To precipitate the peptide, the combined filtrate was added dropwise to cold diethyl ether (35 mL for 0.025 mmol resin) followed by centrifugation at 4000 rpm for 10 min. Then the diethyl ether was decanted, followed by dissolution of the peptide in 3 mL 25% acetonitrile/ 0.1 % TFA water, and lyophilized to get white powder. The crude dry peptide powder was purified by RP-HPLC (Method B as described in Section 1.5) to provide Max(22-60)-Nbz (**2**) as a white powder (13%).

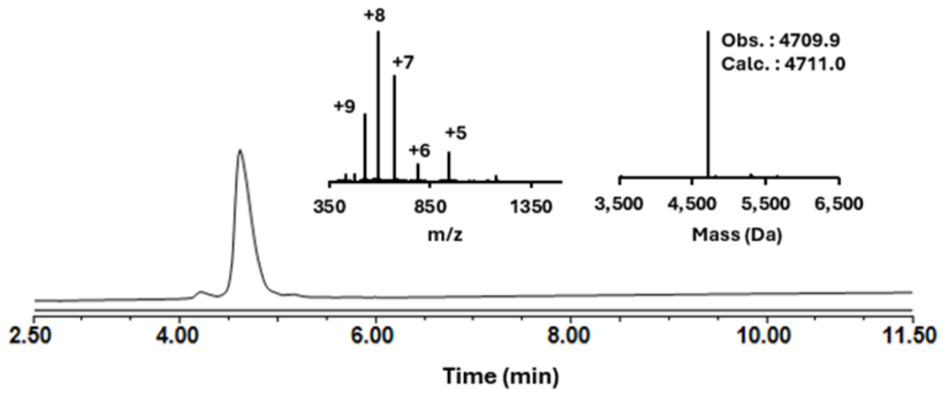

Figure S3. LC-MS analysis of Max(22-60)-Nbz fragment (2). LC chromatogram of the UV absorbance at 214 nm, mass-to-charge (m/z) spectrum and deconvoluted mass spectrum. LC-MS analysis was carried out as described in section 1.3.

4.4 Synthesis of Cys-Myc(400-434) fragment (3)

The synthesis was carried out according to the following scheme:

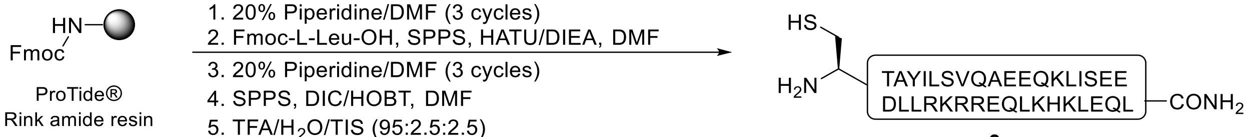

3

The synthesis of Cys-Myc(400-434) fragment (**3**) was carried out on Rink amide ProTide® (LL) resin (556 mg, loading 0.18 mmol/g, 0.1 mmol scale). The resin was pre-swollen in DMF (5 mL) for 30 min and then treated with 5 mL 20 % piperidine (5 min x 3), followed by washing with DMF (10 mL x 3). The first amino acid was coupled manually with Fmoc-L-Leu-OH (5 equiv.), using HATU (5 equiv.) and 250 µL DIEA in 2.5 mL DMF for 40 min at room temperature. Then, all amino acids were coupled manually with Fmoc-L-AA-OH (5 equiv.) using HOBt (5 equiv.) and DIC (160 µL) at room temperature for 45 min. After coupling all amino acids, the peptidyl bound resin was washed with DMF (10 mL x 3) and DCM (10 mL x 3) and kept drying under a vacuum. Subsequently, a mixture of TFA/H_2O/TIS (95/2.5/2.5, 5 mL for 0.025 mmol scale) was added to the resin which was shaken for 3 h at room temperature. The resin was removed by filtration and was washed with additional TFA (1 mL x 2). To precipitate the peptide, the combined filtrate was added dropwise to cold diethyl ether (35 mL for 0.025 mmol resin) followed by centrifugation at 4000 rpm for 10 min. Then the diethyl ether was decanted, followed by dissolution of the peptide in 3 mL 25% acetonitrile/0.1% TFA water and lyophilized to get white powder. The crude dry peptide powder was purified by RP-HPLC (Method B as described in Section 1.5) to provide Cys-Myc(400-434) fragment (**3**) as a white powder (35%).

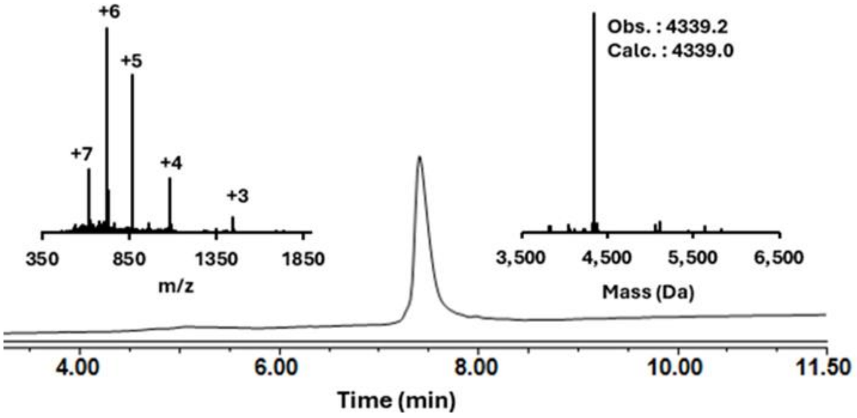

Figure S4. LC-MS analysis of peptide Cys-Myc(400-434) fragment (3). LC chromatogram of the UV absorbance at 214, mass-to-charge (m/z) spectrum and deconvoluted mass spectrum. LC-MS analysis was carried out as described in section 1.3.

4.5 Synthesis of Myc(353-398)-Nbz fragment (4)

The synthesis was carried out according to the following scheme:

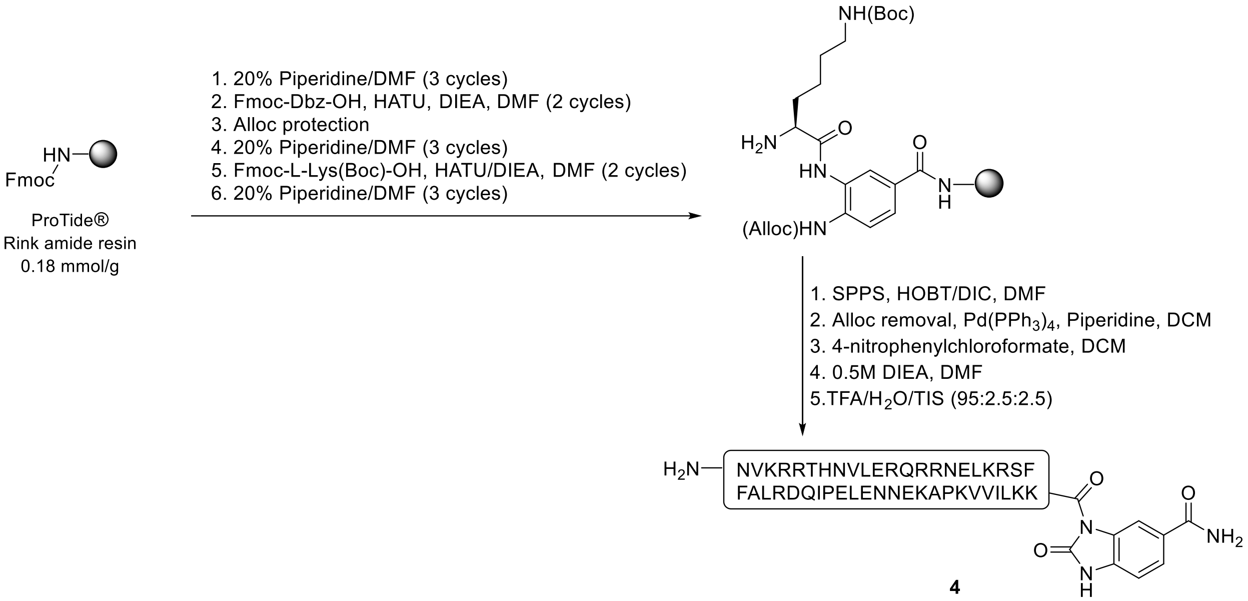

The synthesis of Myc(353-398)-Nbz (**4**) was carried out using Rink amide ProTide® (LL) (0.18 mmol/g, 0.05 mmol scale). The resin was pre-swollen in DMF (5 mL) for 30 min, treated with 5 mL 20% piperidine/DMF (5 min x 3), washed with DMF (10 mL x 3) and then coupled with Fmoc-Dbz-OH (5 equiv.), using HATU (5 equiv.) and 0.25 mL DIEA for 45 min (2 cycle). Subsequently, the resin was washed with DMF (5 mL x 3) and DCM (5 mL x 3) and was treated with a solution of Allyl chloroformate (50 equiv.) and DIEA (2 equiv.) in 2.5 mL DCM for 12 h at room temperature. For Fmoc removal, the resin was treated with 5 mL 20 % piperidine (contains 0.05 % F.A.) (5 min x 3). Then, the first amino acid was manually coupled with Fmoc-L-Lys(Boc)-OH (5equiv.) using HATU (5 equiv.) and DIEA (0.25 mL) for 45 min (2 cycle). The remaining sequence was coupled manually with Fmoc-L-AA-OH (5 equiv.), using HOBt (5 equiv.) and DIC (160 µL) for 45 mins. The peptidyl bound resin was washed with DMF (10 mL x 3) and DCM (10 mL x 3). For Alloc removal, the resin was treated with a solution of piperidine (1.0 mL) and $Pd(PPh_3)_4$ (57.8 mg, 1 equiv.) in 4 mL DCM and was shaken for 50 min at room temperature. The resin was washed with DCM (5 mL x 3), and a solution of 4-nitrophenylchloroformate (5 equiv.) in 1.5 mL of DCM was added to resin. The resin was shaken for 30 min (3 cycles) at room temperature and washed with DCM (5 mL x 3) and DMF (5 mL x 3). To the washed resin, a solution of 0.5 M DIEA in DMF was added and shaken for additional 10 min (3 cycles) to complete the cyclization. The resin was washed with DMF (5 mL x 3), DCM (5 mL x 3) and kept drying under vacuum. Subsequently, a mixture of TFA/H_2O/TIS (95/2.5/2.5, 8 mL for 0.05 mmol scale) was added to the resin which was shaken for 3 h at room temperature. The resin was removed by filtration and was washed with additional TFA (1 mL x 2). To precipitate the

peptide, the combined filtrate was added dropwise to cold diethyl ether (35 mL for 0.05 mmol resin) followed by centrifugation at 4000 rpm for 10 min. Then, the ether was decanted, followed by dissolution of the peptide in 3 mL 25% acetonitrile/0.1% TFA water and lyophilized to get white powder. The crude dry peptide powder was purified by RP-HPLC (Method B as described in Section 1.5) to provide Myc(353-398)-Nbz (**4**) as a white powder (8.2%).

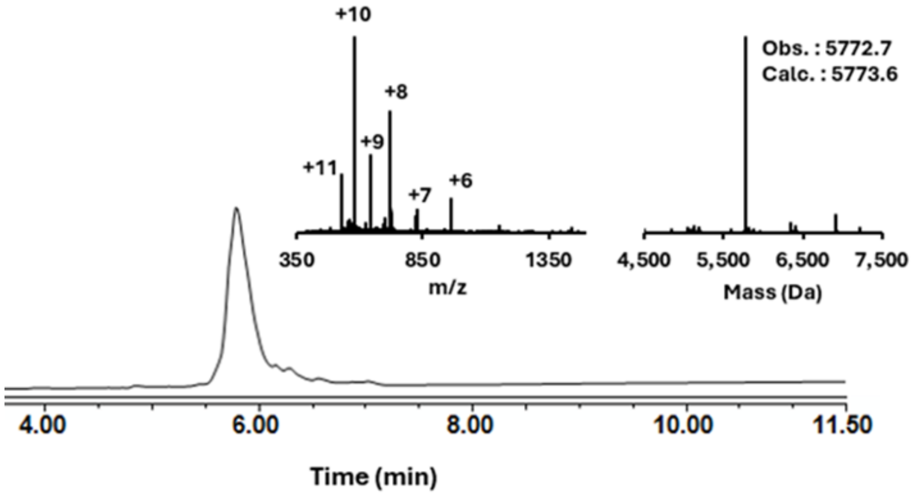

Figure S5. LC-MS analysis of peptide Myc(353-398)-Nbz fragment (4). LC chromatogram of the UV absorbance at 214 nm, mass-to-charge (m/z) spectrum and deconvoluted mass spectrum. LC-MS analysis was carried out as described in section 1.3.

5. Model protein synthesis

5.1 Chemical synthesis of MaxA61C analog

The synthesis was carried out according to the following scheme:

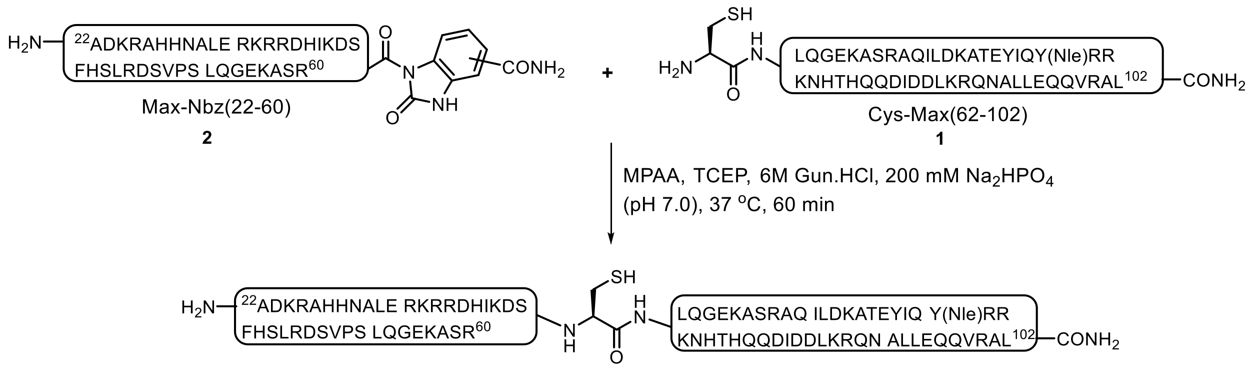

MaxA61C

To a 1.5 mL Eppendorf, Cys-Max(62-102) fragment **(1)** (10.8 mg, 1.0 equiv.) was dissolved in 6 M Gun·HCl, 200 mM Na_2HPO_4 buffer (1.15 mL, 2 mM) containing MPAA (90 mM.) and TCEP (60 mM) at pH 7.0, and Max(22-60)-Nbz fragment **(2)** (15 mg, 1.5 equiv.) was added in one portion. The Eppendorf was closed, and the reaction mixture was incubated at 37 ºC for 60 min. The reaction was monitored by LC-MS as described in Section 1.3. LC-MS analysis showed the formation of **MaxA61C** and the consumption of the Cys-Max(62-102) fragment (**1**). Finally, the ligation solution was diluted to 1 mM using 0.1% TFA water, and was purified by RP-HPLC (Method C as described in section 1.5) to afford pure MaxA61C (10.8 mg) as a white powder (53%).

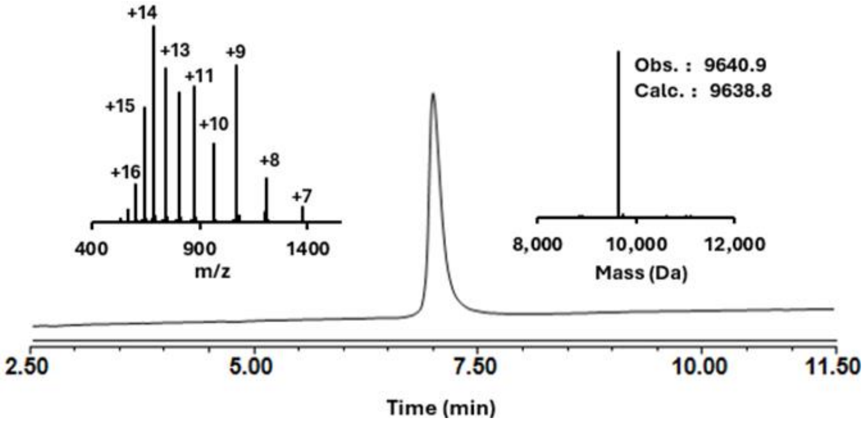

Figure S6. LC-MS analysis of isolated MaxA61C. LC chromatogram of the UV absorbance at 214 nm, mass-to-charge (m/z) spectrum and deconvoluted mass spectrum. LC-MS analysis was carried out as described in section 1.3.

5.2 Chemical synthesis of MycA399C analog

The synthesis was carried out according to the following scheme:

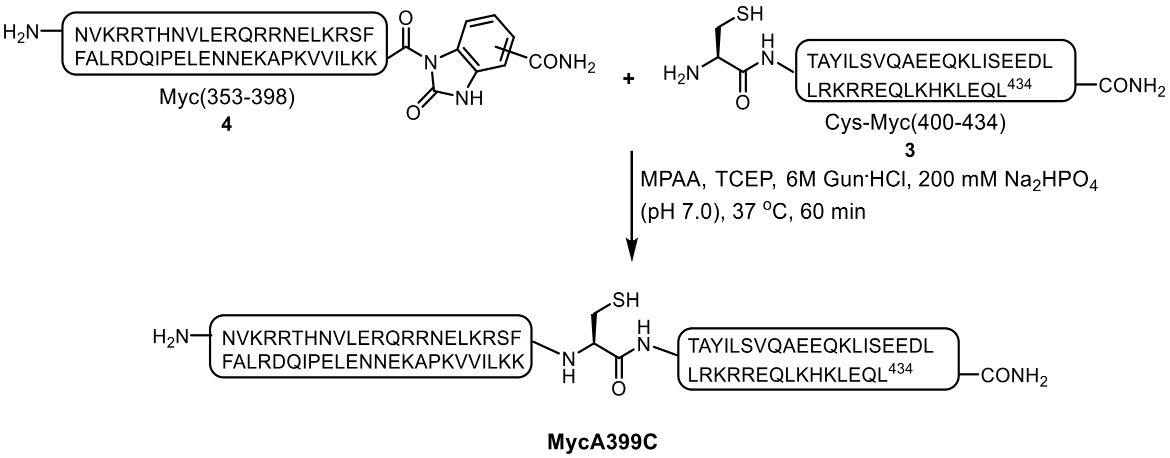

To a 1.5 mL Eppendorf, Cys-Myc (400-434) fragment (**3**) (10.0 mg, 1 equiv.) was dissolved in 6 M Gun·HCl, 200 mM Na_2HPO_4 buffer (1.15 mL, 2 mM) at pH = 7, and then Myc(353-398)-Nbz fragment (**4**) (20.0 mg, 1.5 equiv.) was added in one portion. The Eppendorf was closed, and the reaction mixture was incubated at 37 ºC for 60 min. The reaction was monitored by LC-MS as described in Section 1.3. LC-MS analysis showed the formation of ligated **MycA399C** and the consumption of the Cys-Myc(400-434) fragment (**3**). Finally, the ligation solution was diluted to 1 mM using 0.1% TFA water and was purified by RP-HPLC (Method C as described in section 1.5) to afford pure **MycA399C** (10.1 mg) as a white powder (44%).

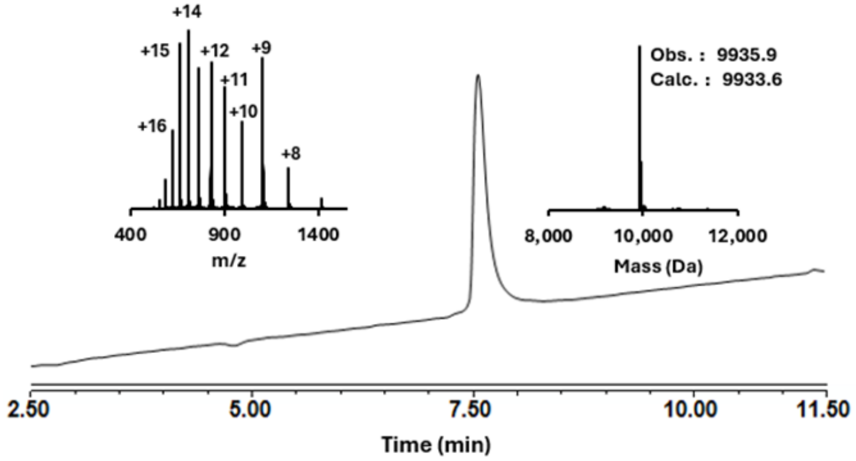

Figure S7. LC-MS analysis of isolated MycA399C. LC chromatogram of the UV absorbance at 214 nm, mass-to-charge (m/z) spectrum and deconvoluted mass spectrum. LC-MS analysis was carried out as described in section 1.3.

6. Site-specific installation of nitration and phosphorylation analogs

6.1 Nitration of model peptide P1 with Pd-1

The reaction was carried out according to the following scheme:

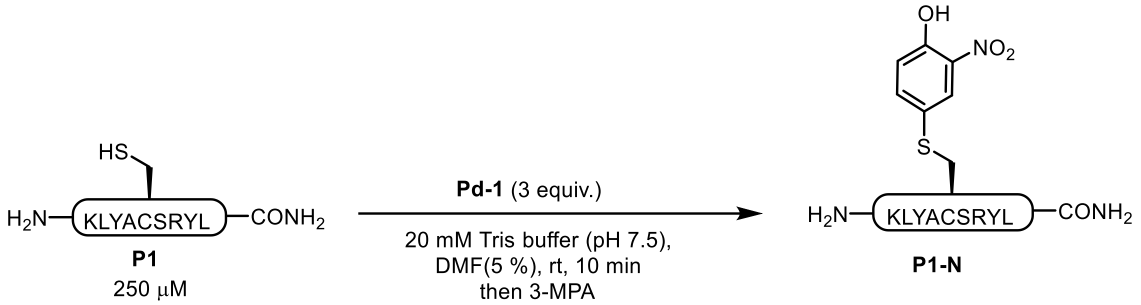

To a 15 mL falcon tube was added **P1** (1.1 mg, 3.75 mL, 0.263 mM, 1.0 equiv.) in 20 mM Tris (pH 7.5) buffer, and **Pd-1** (2.3 mg, 197 µL, 15.0 mM, 3.0 equiv.) as a solution in DMF (v = 5 %) was added in one portion. The final reaction concentrations of the major reaction components were as follows: **P1** (0.25 mM); **Pd-1** (0.75 mM). The falcon tube was closed, and the reaction mixture was vortexed for a few seconds and then kept at room temperature for 15 min. Next, the falcon tube was opened, and the reaction was treated with 3-MPA (10 equiv. compared to **P1**) and was kept at room temperature for 10 min. 20 µL of the reaction mixture was injected into Analytical HPLC and analyzed as described in Section 1.2. The mass spectrum was acquired by using Thermo Scientific ISQ EM Mass spectrometer.

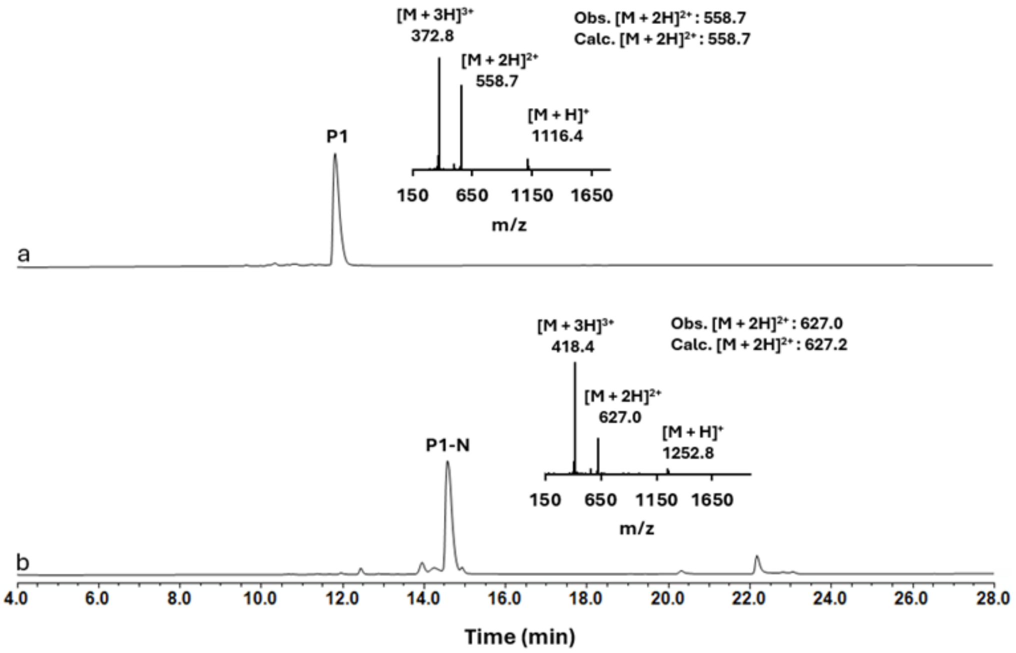

Figure S8. HPLC and MS analysis of nitration of model peptide P1 with Pd-1. HPLC chromatogram of the UV absorbance at 214 nm and mass-to-charge (m/z) spectrum. HPLC analysis was carried out as described in section 1.2. (a) model peptide **P1** at t = 0. (b) crude nitration reaction with **Pd-1** at t = 10 min.

6.2 Phosphorylation of model peptide P1 with Pd-2

The reaction was carried out according to the following scheme:

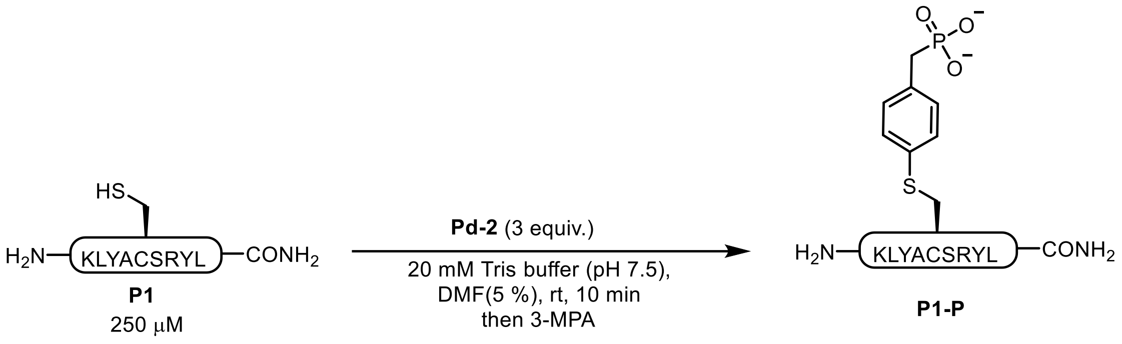

To a 1.5 mL Eppendorf was added **P1** (85 µL, 263.8 µM, 1.0 equiv.) in 20 mM Tris (pH 7.5) buffer, and then **Pd-2** (4.5 µL, 14.5 mM, 3.0 equiv.) in DMF (v = 5 %) as a solution was added in portion. The final reaction concentrations of the major reaction components were as follows: **P1** (0.25 mM) and **Pd-2** (0.75 mM). The reaction mixture was vigorously stirred for a few seconds and then kept at room temperature for 10 min. Next, the Eppendorf was opened, and the reaction was treated with 3-MPA solution (10 equiv. compared to **P1**) and was kept at room temperature for 10 min. 20 µL of the reaction mixture was injected to Analytical HPLC and analyzed as described in Section 1.2. The mass spectrum was acquired by using Thermo Scientific ISQ EM Mass spectrometer.

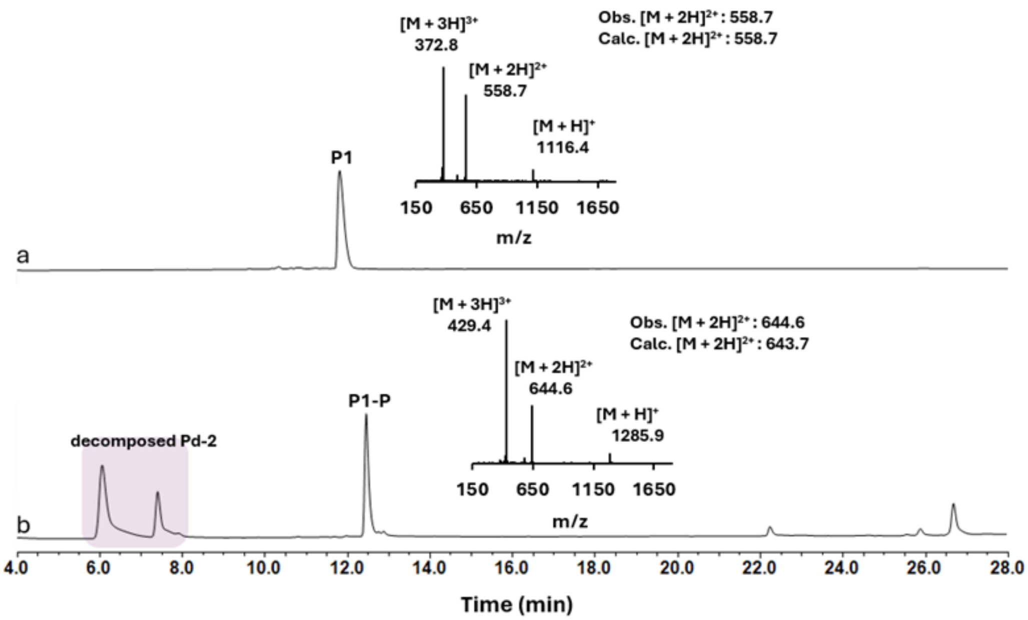

Figure S9. HPLC and MS analysis of the phosphorylation of model peptide P1 with Pd-2. HPLC chromatogram of the UV absorbance at 214 nm and mass-to-charge (m/z) spectrum. HPLC analysis was carried out as described in section 1.2. (a) peptide **P1** at t = 0. (b) crude phosphorylation reaction with **Pd-2** at t = 10 min. * **Pd-2** decomposition by-product.

7. Site-specific phosphorylation and nitration of model proteins

7.1 Site-specific phosphorylation of MaxA61C with Pd-2

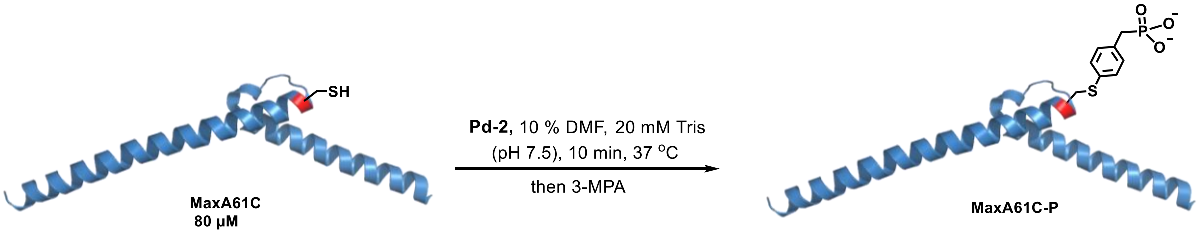

To a 1.5 mL Eppendorf was added **MaxA61C** in 20 mM Tris buffer (pH 7.5) (58.3 µL, 88.9 µM, 1.0 equiv.), and the mixture was preheated at 37 °C for 1 min. **Pd-2** (6.5 µL, 6.1 mM, 7.5 equiv.) as a solution in DMF (v = 10 %) was added. The final reaction concentrations of the major reaction components were as follows: **MaxA61C** (80 µM) and **Pd-2** (605.7 µM). The reaction mixture was mixed by pipetting up and down 10× followed by a 10 min incubation at 37 °C. Next, the Eppendorf was opened and the reaction was treated with 3-MPA (30 equiv. compared to **MaxA61C**) and was kept at room temperature for 10 min. 10 µL of reaction mixture was injected into LCMS and analyzed as described in Section 1.3. Results are depicted in Figure S10 a-b.

7.2 Site-specific Nitration of MaxA61C with Pd-1

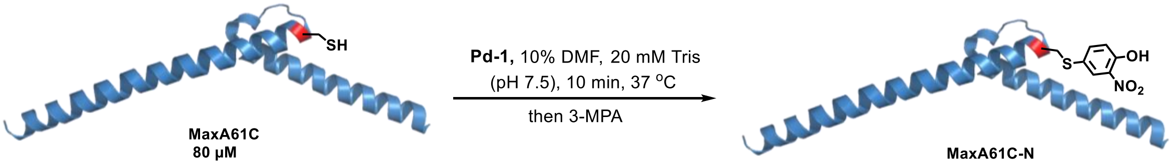

To a 1.5 mL Eppendorf was added **MaxA61C** in 20mM Tris buffer (pH 7.5) (58.3 µL, 88.9 µM, 1.0 equiv.), and preheated at 37 °C for 1 min. Then **Pd-1** (6.5 µL, 8.0 mM, 10 equiv.) as a solution in DMF (v = 10 %) was added in one portion. The final reaction concentrations of the major reaction components were as follows: **MaxA61C** (80 µM) and **Pd-1** (801 µM). The reaction mixture was mixed by pipetting up and down 10× followed by a 10 min incubation at 37 °C. Next, the Eppendorf was opened and the reaction was treated with 3-MPA (30 equiv. compared to **MaxA61C**) and was kept at room temperature for 10 min. 10 µL of the reaction mixture was injected into LCMS and analyzed as described in Section 1.3. Results are depicted in Figure S10 c.

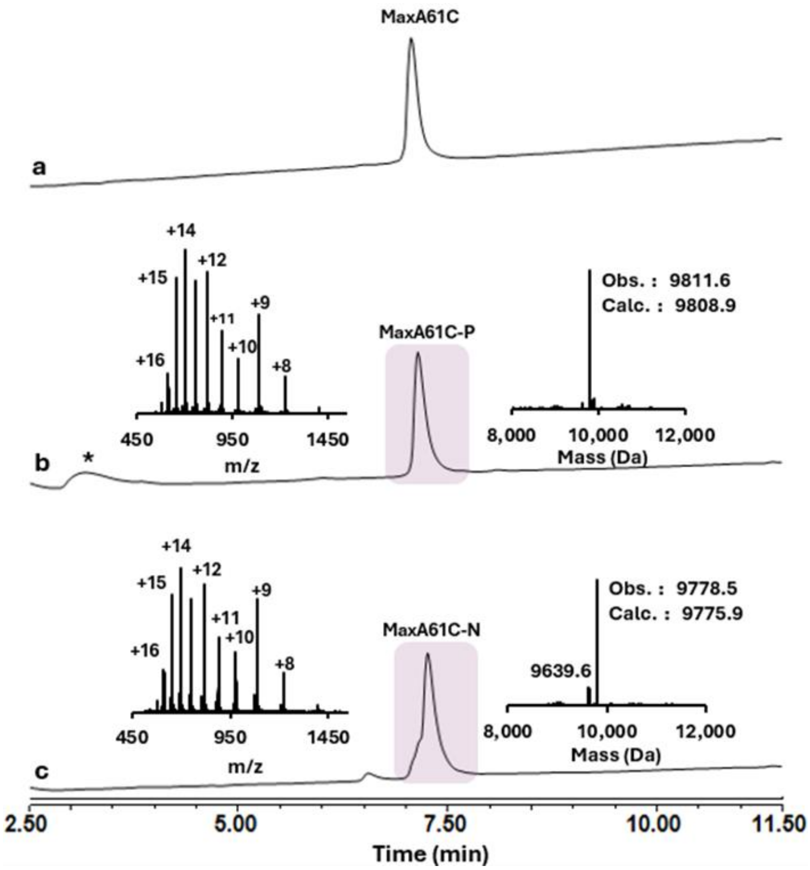

Figure S10. LC-MS analysis of the phosphorylation and nitration reactions of MaxA61C. LC chromatogram of the UV absorbance at 214 nm, mass-to-charge (m/z) spectrum and deconvoluted mass spectrum. LC-MS analysis was carried out as described in section 1.3. (a) **MaxA61C** at t = 0. (b) crude phosphorylation reaction of **MaxA61C** with **Pd-2** at t = 10 min. (c) crude nitration reaction of **MaxA61C** with **Pd-1** at t = 10 min. * Pd-2 decomposition by-product.

7.3 Site-specific phosphorylation of MycA399C with Pd-2

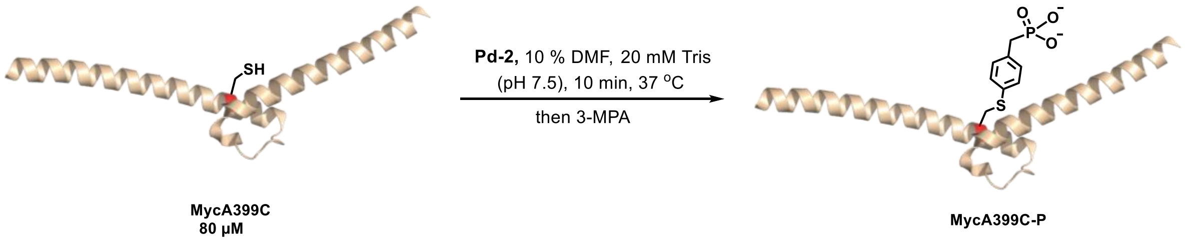

To a 1.5 mL Eppendorf was added **MycA399C** in 20 mM Tris buffer (pH 7.5) (56.6 μL, 88.9 μM, 1.0 equiv.), and **MycA399C** solution was preheated at 37 °C for 1 min and followed by adding **Pd-2** (6.3 μL, 6.01 mM, 7.5 equiv.) as a solution in DMF (v = 10 %) in one portion. The final reaction concentrations of the major reaction components were as follows: **MycA399C** (80 μM); **Pd-2** (607.8 mM). The reaction mixture was mixed by pipetting up and down 10× followed by a 10 min incubation at 37 °C. Next, the Eppendorf was opened, and the reaction was treated with 3-MPA (30 equiv. compared to **MycA399C**) and was kept at room temperature for 10 min. 10 μL of reaction mixture was injected to LCMS and analyzed as described in section 1.3. Results are depicted in Figure S11 a-b.

7.4 Site-specific Nitration of MycA399C with Pd-1

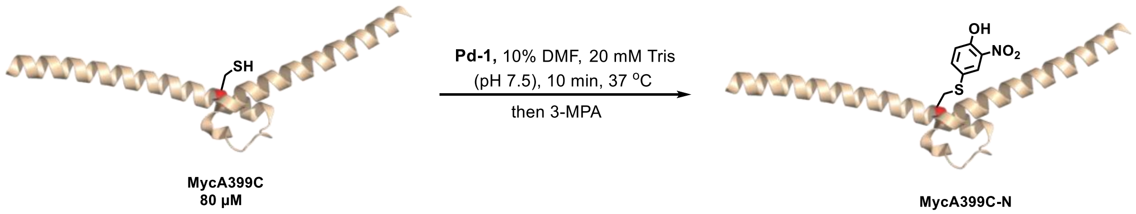

To a 1.5 mL Eppendorf was added **MycA399C** in 20 mM Tris buffer (pH 7.5) (56.6 μL, 88.9 μM, 1.0 equiv.), solution and preheated at 37 °C for 1 min and followed by adding **Pd-1** (6.3 μL, 8.1 mM, 10 equiv.) as a solution in DMF (v = 10 %) in one portion. The final reaction concentrations of the major reaction components were as follows: **MycA399C** (80 μM); **Pd-1** (805 μM). The reaction mixture was mixed by pipetting up and down 10× followed by a 10 min incubation at 37 °C. Next, the Eppendorf was opened, and the reaction was treated with 3-MPA (30 equiv. compared to **MycA399C**) and was kept at room temperature for 10 min. 10 μL of reaction mixture was injected to LCMS and analyzed as described in section 1.3. Results are depicted in Figure S11 c.

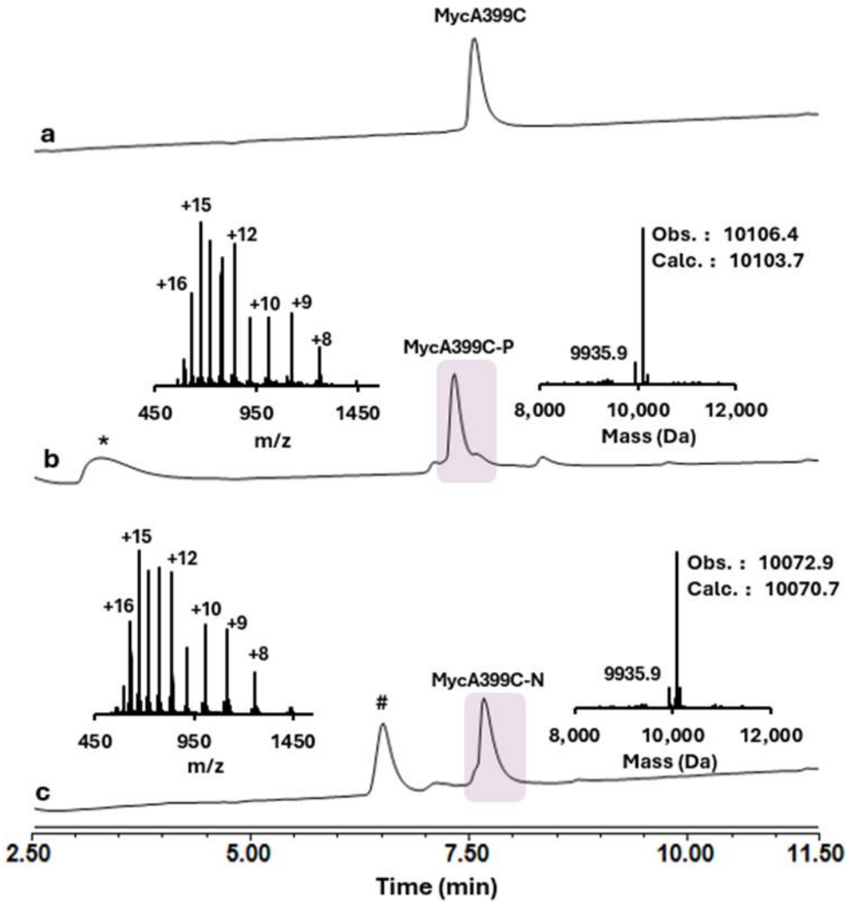

Figure S11. LC-MS analysis of the phosphorylation and nitration reactions of MycA399C. LC chromatogram of the UV absorbance at 214 nm, mass-to-charge (m/z) spectrum and deconvoluted mass spectrum. LC-MS analysis was carried out as described in section 1.3. (a) **MycA399C** at t = 0. (b) crude phosphorylation reaction of **MycA399C** with **Pd-2** at t = 10 min. (c) crude nitration reaction of **MycA399C** via **Pd-1** at t = 10 min. * Pd-2 decomposition by-product. # Pd-1 decomposition by-product.

8. Isolation of modified MaxA61C analogs and stability analysis

8.1 Isolation of phosphorylated MaxA61C

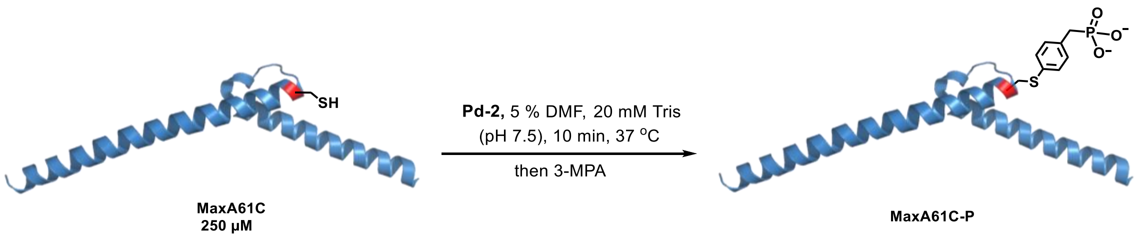

To a 15 mL falcon tube was added **MaxA61C** (6.0 mg, 2.37 mL, 262.7 μM, 1.0 equiv.) in 20 mM Tris buffer (pH 7.5) solution and preheated at 37 °C for 1 min. **Pd-2** (3.8 mg, 124.5 μL, 31 mM, 6.2 equiv.) as a solution in DMF (v = 5%) was added in one portion. The final reaction concentrations of the major reaction components were as follows: **MaxA61C** (250 μM); **Pd-2** (1.55 mM). The reaction mixture was vigorously stirred for a few seconds and followed by a 10 min incubation at 37 °C. Next, the falcon tube was opened, and the reaction was treated with 3-MPA (10 equiv. compared to **MaxA61C**), and then was kept at room temperature for 10 min. 10 μL of the reaction mixture was injected to LCMS and analyzed as described in section 1.3. The reaction mixture was purified by RP-HPLC using Method C (see section 1.5) affording the desired phosphorylated protein **MaxA61C**-P 3.9 mg as white powder (65%).

^{31}P NMR (162 MHz, D_2O) δ 21.6 (s).

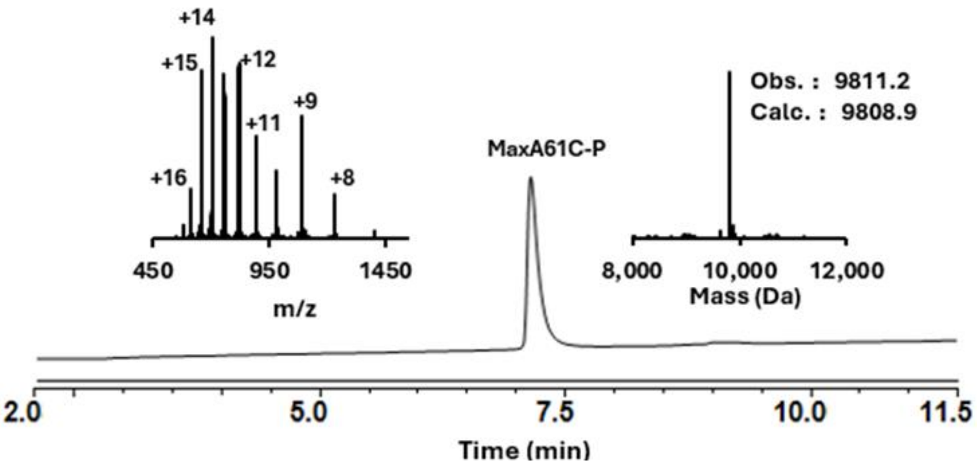

Figure S12. LC-MS analysis of isolated MaxA61C-P. LC chromatogram of the UV absorbance at 214 nm, mass-to-charge (m/z) spectrum and deconvoluted mass spectrum. LC-MS analysis was carried out as described in section 1.3.

8.2 Isolation of nitrated MaxA61C

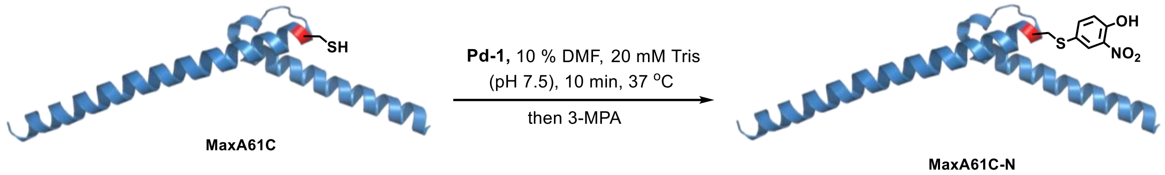

To a 15 mL falcon tube was added **MaxA61C** (2.5 mg, 3.6 mL, 69.9 µM, 1.0 equiv.) in 20 mM Tris buffer (pH 7.5) buffer and preheated at 37 °C for 1 min. **Pd-1** (3.0 mg, 400 µL, 9.5 mM, 15 equiv.) as a solution in DMF (v = 10%) was added in one portion. The final reaction concentrations of the major reaction components were as follows: **MaxA61C** (62.5 µM); **Pd-1** (937.5 µM). The reaction mixture was vigorously stirred for a few seconds and followed by a 10 min incubation at 37 °C. Next, the falcon tube was opened, and the reaction was treated with 3-MPA (20 equiv. compared to **MaxA61C**) and was kept at room temperature for 10 min. 10 µL of the reaction mixture was injected into LCMS and analyzed as described in section 1.3. The reaction mixture was purified by RP-HPLC by using the Method C (see section 1.5) affording the desired **MaxA61C-N** 1.6 mg as white powder (64%).

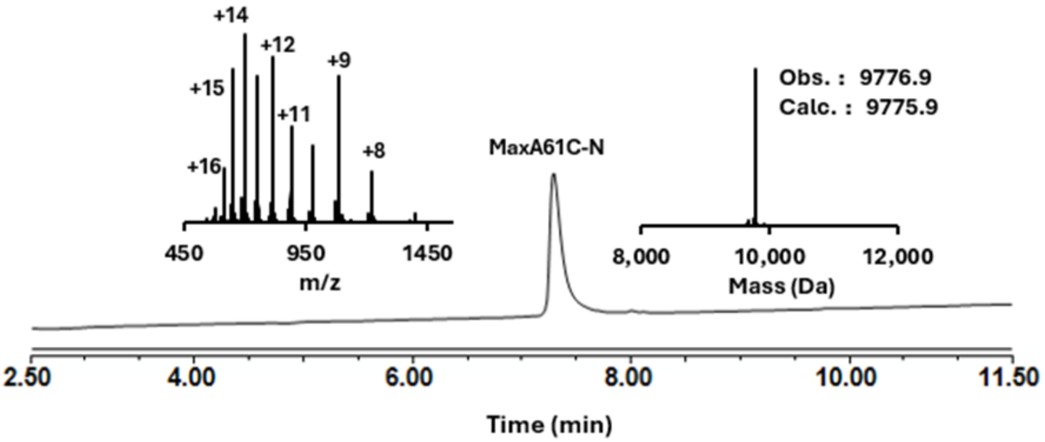

Figure S13. LC-MS analysis of isolated MaxA61C-N. LC chromatogram of the UV absorbance at 214 nm, mass-to-charge (m/z) spectrum and deconvoluted mass spectrum. LC-MS analysis was carried out as described in section 1.3.

8.3 Stability of isolated MaxA61C-P

Isolated **MaxA61C-P** was dissolved in PBS buffer (x1) (255 µL, C = 200 µM, pH 7.0) and the solution was analyzed by LC-MS as described in section 1.3. The solution was incubated at 37 °C and was monitored by LC-MS using the same method after 1 day, 3 days, and 5 days. The results are depicted in Figure S14 left column.

8.4 Stability of isolated MaxA61C-N

Isolated **MaxA61C-N** was dissolved in PBS buffer (x1) (255 µL, C = 200 µM, pH 7.0) and the solution was analyzed by LC-MS as described in section 1.3. The solution was incubated at 37 °C and was monitored by LC-MS using same method after 1day, 3 days, and 5 days. The results are depicted in Figure S14 right column.

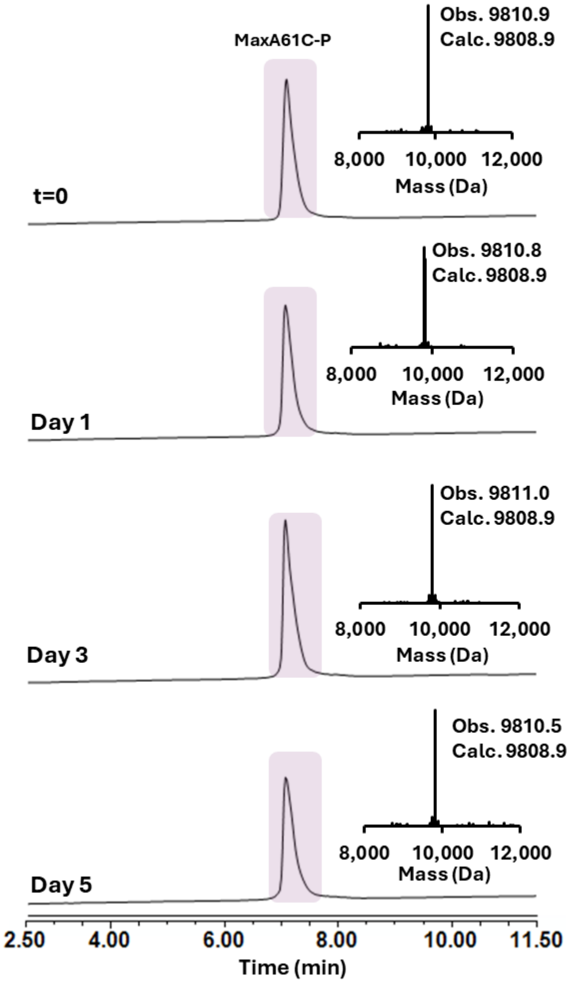

Figure S14. LC-MS analysis of the stability of isolated MaxA61C-P and isolated MaxA61C-N. LC chromatogram of the UV absorbance at 214 nm, mass-to-charge (m/z) spectrum, and deconvoluted mass spectrum. LC-MS analysis was carried out as described in section 1.3. (a) left column: stability of **MaxA61C-P**. (b) right column: stability of **MaxA61C-N**.

9. Site-specific α-Syn phosphorylation and nitration

9.1 Late-stage α-Syn phosphorylation

9.1.1 α-SynY39C phosphorylation with Pd-2

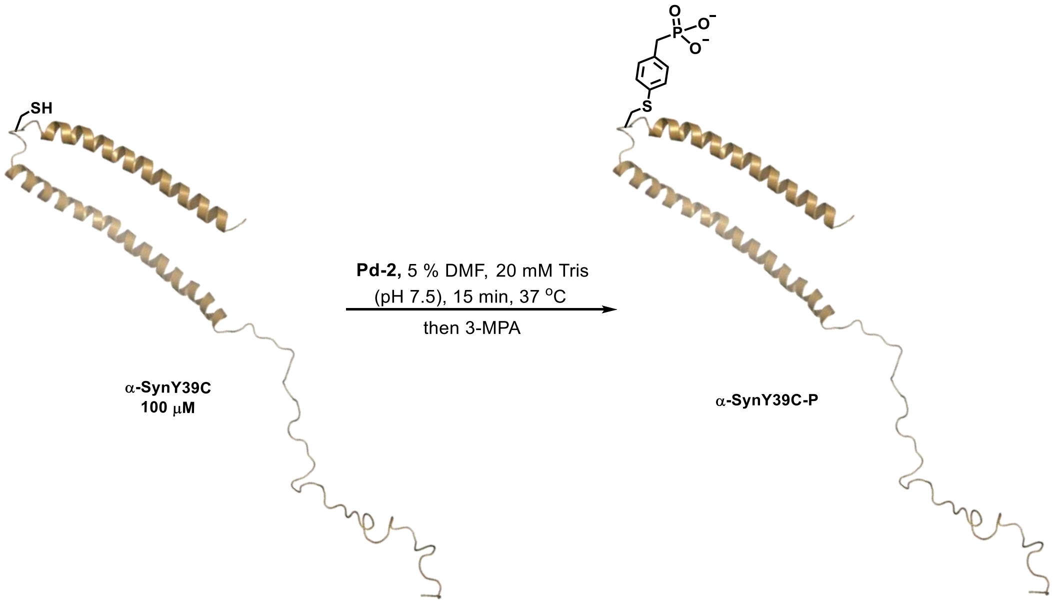

To a 15 mL falcon tube was added **α-SynY39C** (4.5 mg, 2.97 mL, 105.3 µM, 1.0 equiv.) in 20 mM Tris buffer (pH 7.5), and the solution was preheated at 37 °C for 1 min. **Pd-2** (1.9 mg, 156.2 µL, 12.2 mM, 6.1 equiv.) as a solution in DMF (v = 5%) was added in one portion. The final reaction concentrations of the major reaction components were as follows: **α-SynY39C** (100 µM); **Pd-2** (610 µM). The reaction mixture was vigorously stirred for a few seconds and then incubated at 37 °C for 15 min. Then, 3-MPA (0.4 µL, 15 equiv. compared with **α-SynY39C)** was added and the reaction mixture was kept at 37 °C for 15 min to quench the reaction. The reaction mixture was purified by RP-HPLC using method A (see section 1.5) affording the desired **α-SynY39C-P** product 3.3 mg (73%) as a white powder.

^{31}P NMR (162 MHz, D_2O) δ 21.4 (s).

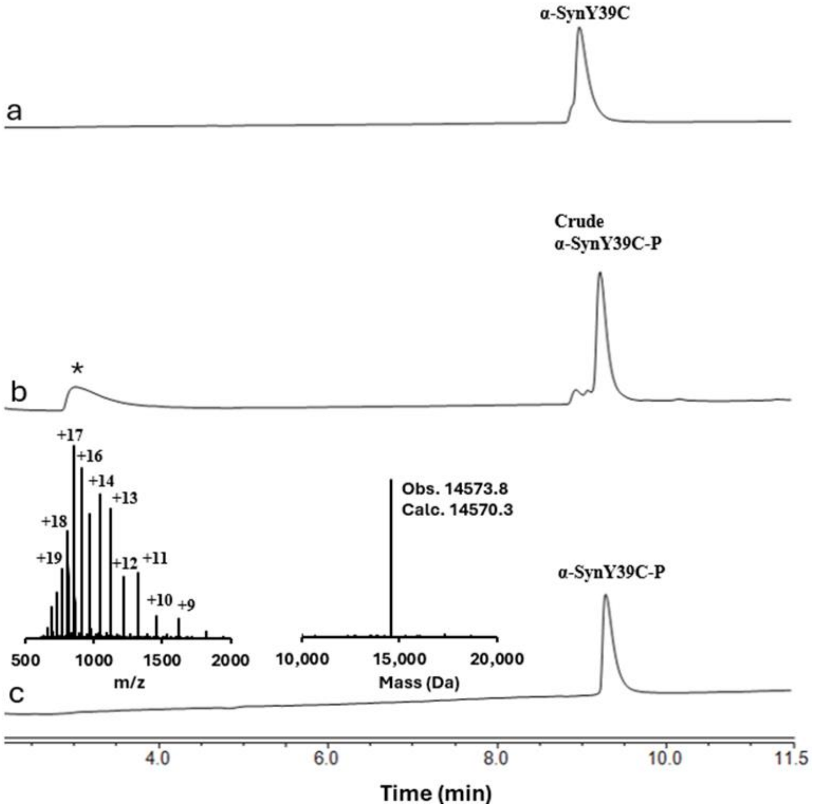

Figure S15. LC-MS analysis of the phosphorylation reaction and isolation of modified α-SynY39C. LC chromatogram of the UV absorbance at 214 nm, mass-to-charge (m/z) spectrum, and deconvoluted mass spectrum. LC-MS analysis was carried out as described in section 1.3. (a) **α-SynY39C** at t = 0. (b) crude phosphorylation reaction of **α-SynY39C** with **Pd-2** at t = 15 min. (c) isolated **α-SynY39C-P**. * decomposed **Pd-2** by-product.

9.1.2 α-SynY125C phosphorylation with Pd-2

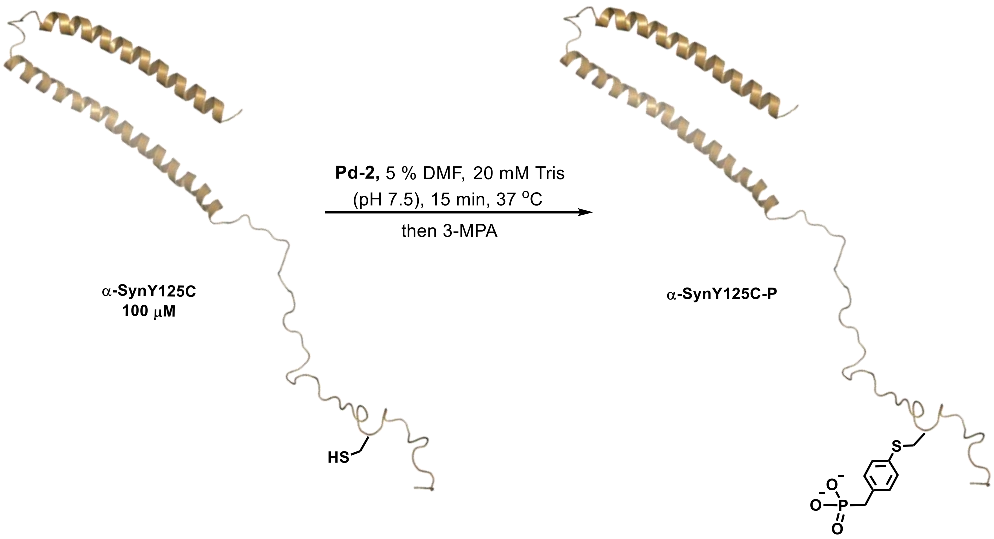

To a 15 mL falcon tube was added **α-SynY125C** (4.2 mg, 2.84 mL, 103 µM, 1.0 equiv.) in 20 mM Tris buffer (pH 7.5), and the solution was preheated at 37 °C for 1 min. Then **Pd-2** (1.8 mg, 146 µL, 12.4 mM, 6.2 equiv.) as a solution in DMF (v = 5%) was added in one portion. The final reaction concentrations of the major reaction components were as follows: **α-SynY125C** (100 µM); **Pd-2** (620 µM). The reaction mixture was vigorously stirred and followed by an incubation at 37 °C for 15 min. Then, 3-MPA (0.4 µL, 15 equiv. compared with **α-SynY125C)** was added and the reaction mixture was kept at 37 °C for 15 min to quench the reaction. The reaction mixture was purified by RP-HPLC using method A (as described in section 1.5) affording the desired product **α-SynY125C-P** 3.5 mg as a white powder (83%).

^{31}P NMR (162 MHz, D_2O) δ 21.9 (s).

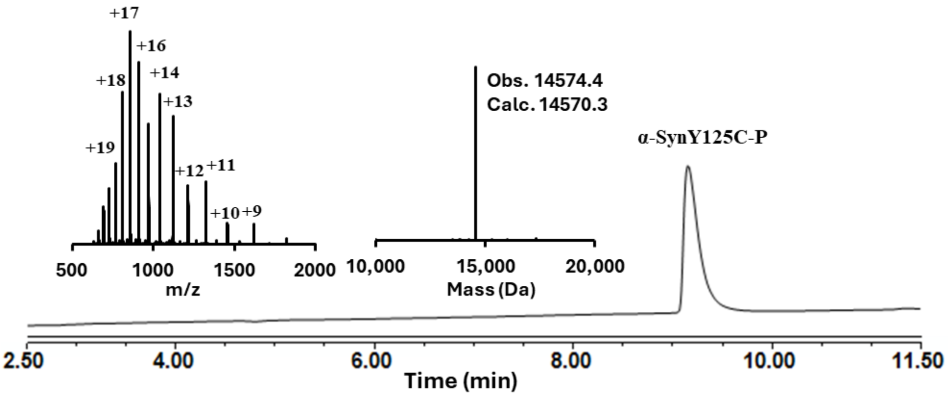

Figure S16. LC-MS analysis of isolated α-SynY125C-P. LC chromatogram of the UV absorbance at 214 nm, mass-to-charge (m/z) spectrum, and deconvoluted mass spectrum. LC-MS analysis was carried out as described in section 1.3.

9.1.3 α-SynY133C phosphorylation with Pd-2

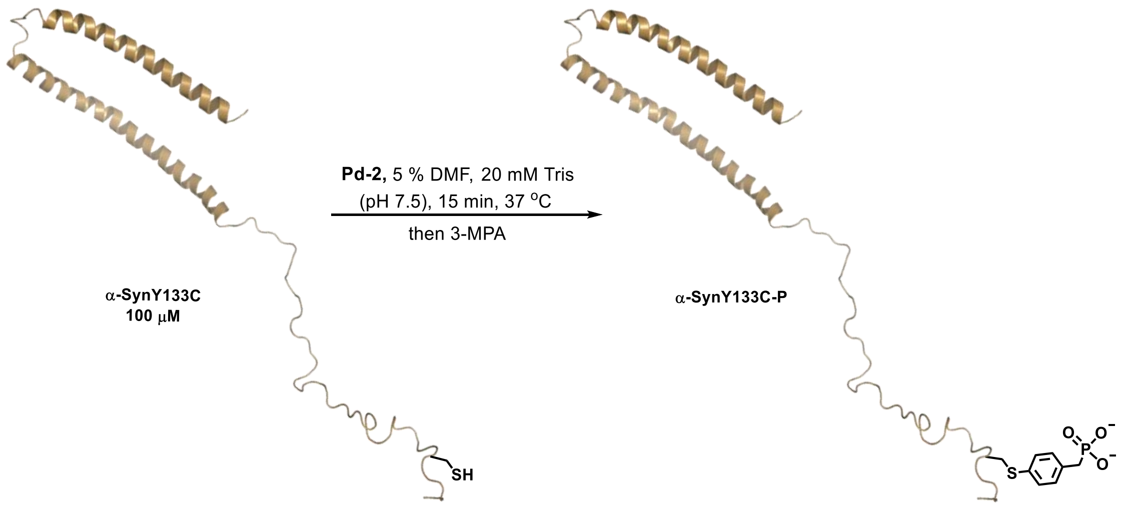

To a 15 mL falcon tube was added **α-SynY133C** (4.3 mg, 2.84 mL, 105.1 µM, 1.0 equiv.) in 20 mM Tris buffer (pH 7.5), and the solution was preheated at 37 °C for 1 min. Then **Pd-2** (1.9 mg, 146 µL, 13.0 mM, 6.4 equiv.) as a solution in DMF (v = 5%) was added in one portion. The final reaction concentrations of the major reaction components were as follows: **α-SynY133C** (100 µM); **Pd-2** (640 µM). The reaction mixture was vigorously stirred for a few seconds and then kept at 37 °C for 15 min. Then, 3-MPA (0.4 µL, 15 equiv. compared with **α-SynY133C)** was added and the reaction mixture was kept at 37 °C for 15 min to quench the reaction. The reaction mixture was purified by RP-HPLC using method A (as described in section 1.5) affording the desired product **α-SynY133C-P** 3.4 mg (79%) as a white powder.

^{31}P NMR (162 MHz, D_2O) δ 20.4 (s).

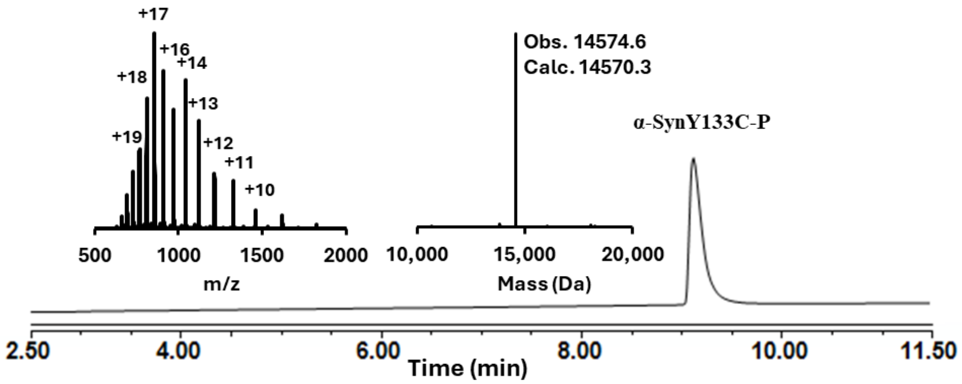

Figure S17. LC-MS analysis of isolated α-SynY133C-P. LC chromatogram of the UV absorbance at 214 nm and mass-to-charge (m/z) spectrum, and deconvoluted mass spectrum. LC-MS analysis was carried out as described in section 1.3.

9.1.4 α-SynY136C phosphorylation with Pd-2

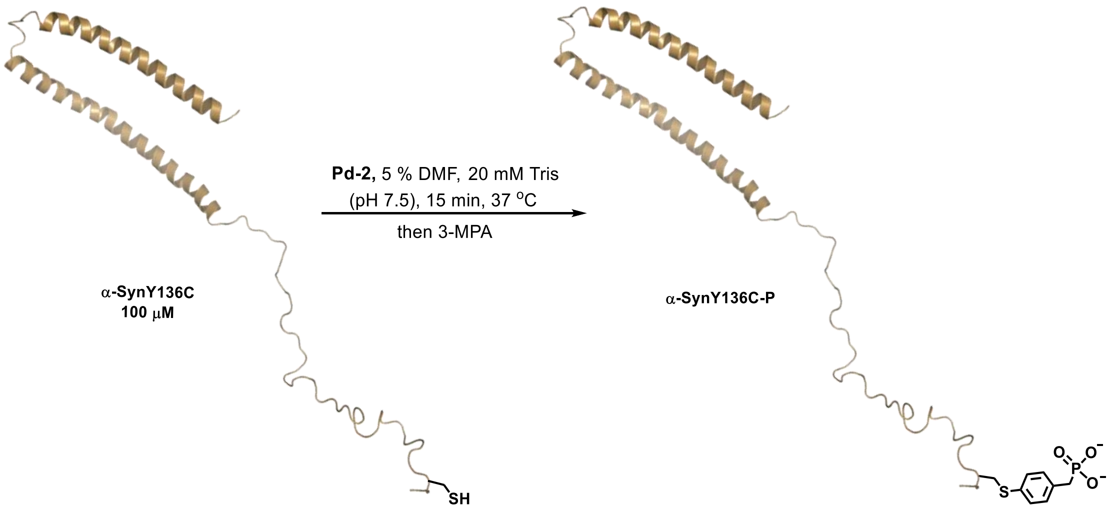

To a 15 mL falcon tube was added **α-SynY136C** (4.3 mg, 2.84 mL, 105.1 μM, 1.0 equiv.) in 20 mM Tris buffer (pH 7.5), and the solution was preheated at 37 °C for 1 min. Then **Pd-2** (1.9 mg, 146 μL, 13.0 mM, 6.5 equiv.) as a solution in DMF (v = 5 %) was added in one portion. The reaction mixture was vigorously stirred for a few seconds and then kept at 37 °C for 20 min. The final reaction concentrations of the major reaction components were as follows: **α-SynY136C** (100 μM); **Pd-2** (650 μM). Then, 3-MPA (0.4 μL, 15 equiv. compared with **α-SynY136C**) was added and the reaction mixture was kept at 37 °C for 20 min to quench the reaction. The reaction mixture was purified by RP-HPLC using method A (as described in section 1.5) affording the desired product **α-SynY136C-P** 3.4 mg (77%) as a white powder.

^{31}P NMR (162 MHz, D_2O) δ 21.9 (s).

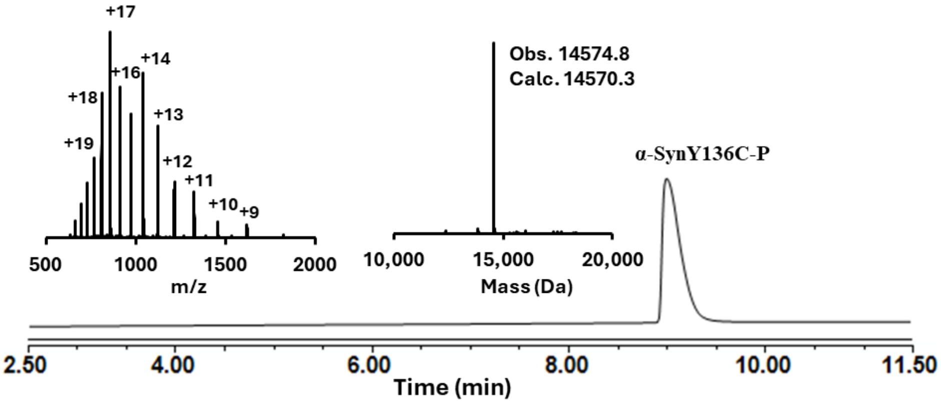

Figure S18. LC-MS analysis of isolated α-SynY136C-P. LC chromatogram of the UV absorbance at 214 nm and mass-to-charge (m/z) spectrum and deconvoluted mass spectrum. LC-MS analysis was carried out as described in section 1.3.

9.2 Late-stage α-Syn nitration

9.2.1 α-SynY39C nitration with Pd-1

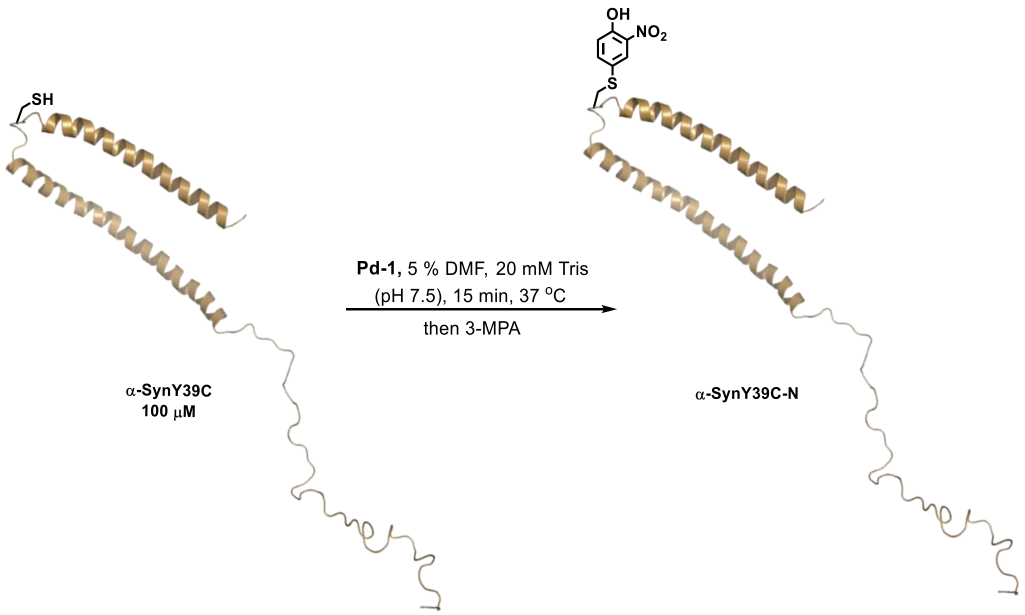

To a 15 mL falcon tube was added **α-SynY39C** (4.0 mg, 2.64 mL, 105.2 μM, 1.0 equiv.) in 20 mM Tris buffer (pH 7.5), and the solution was preheated at 37 °C for 1 min. Then **Pd-1** (2.2 mg, 139 μL, 20.0 mM, 10.0 equiv.) as a solution in DMF (v = 5 %) was added in one portion. The final reaction concentrations of the major reaction components were as follows: **α-SynY39C** (100 μM); **Pd-1** (1 mM). The reaction mixture was vigorously stirred for a few seconds and followed by an incubation at 37 °C for 15 min. After that, 3-MPA (0.5 μL, 20 equiv. compared with **α-SynY39C**) was added and the reaction mixture was kept at 37 °C for 15 min to quench the reaction. The reaction mixture was purified by RP-HPLC using method C (as described in section 1.5) affording the desired product **α-SynY39C-N** 1.7 mg (43%) as a white powder.

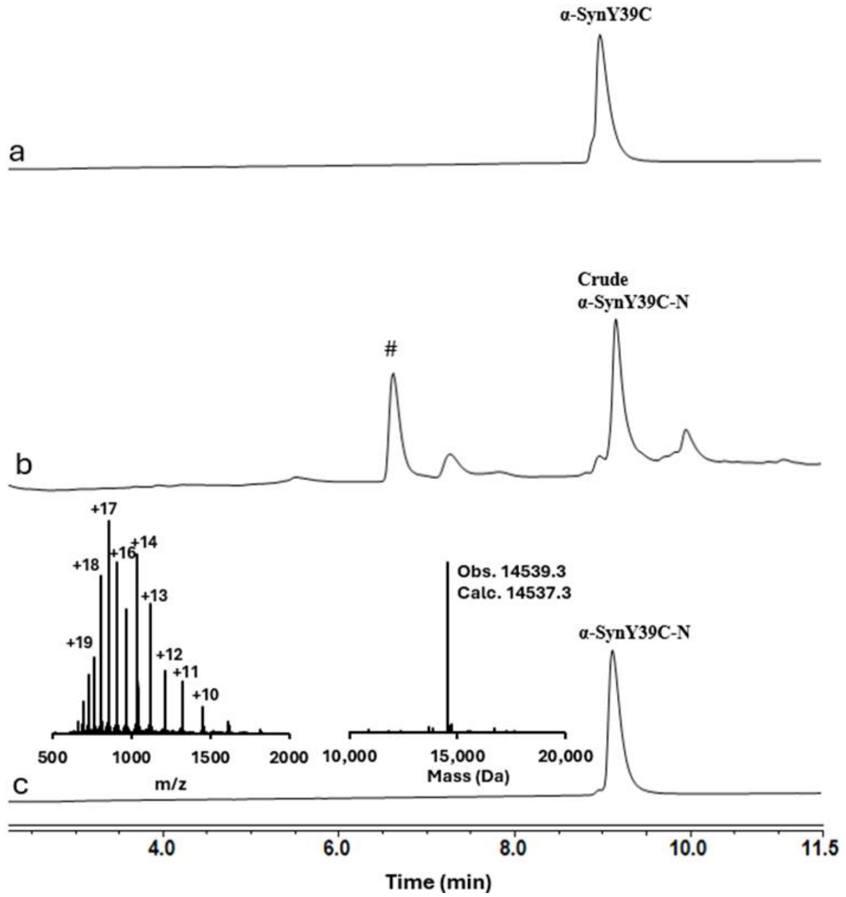

Figure S19. LC-MS analysis of the nitration reaction and isolation of modified α-SynY39C. LC chromatogram of the UV absorbance at 214 nm, mass-to-charge (m/z) spectrum, and deconvoluted mass spectrum. LC-MS analysis was carried out as described in section 1.3). (a) **α-SynY39C** at t = 0. (b) crude nitration of **α-SynY39C** with **Pd-1** at t = 15 min. (c) isolated **α-SynY39C-N.** **#** decomposed **Pd-1** by-product.

9.2.2 α-SynY125C nitration with Pd-1

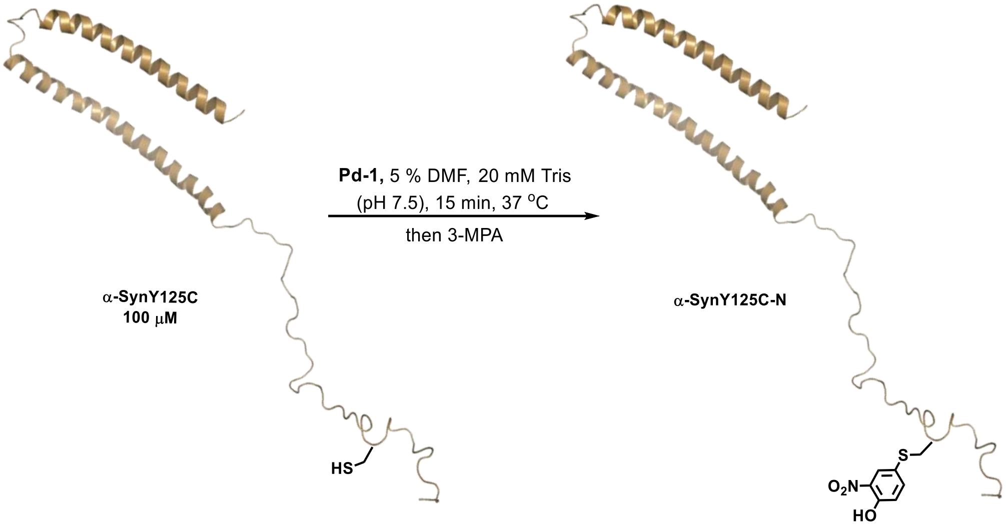

To a 15 mL falcon tube was added **α-SynY125C** (4.0 mg, 2.64 mL, 105.2 µM, 1.0 equiv.) in 20 mM Tris (pH 7.5), and the solution was preheated at 37 °C for 1 min. Then **Pd-1** (2.2 mg, 139 µL, 20.0 mM, 10.0 equiv.) as a solution in DMF (v = 5 %) was added in one portion. The reaction mixture was vigorously stirred for a few seconds and then kept at 37 °C for 15 min. The final reaction concentrations of the major reaction components were as follows: **α-SynY125C** (100 µM); **Pd-1** (1 mM). Then, 3-MPA (0.5 µL, 20 equiv. compared with **α-SynY125C**) was added and the reaction mixture was kept at 37 °C for 15 min to quench the reaction. The reaction mixture was purified by RP-HPLC using method C (as described in section 1.5) affording the desired product **α-SynY125C-N** 1.8 mg as a white powder (45%) as a white powder.

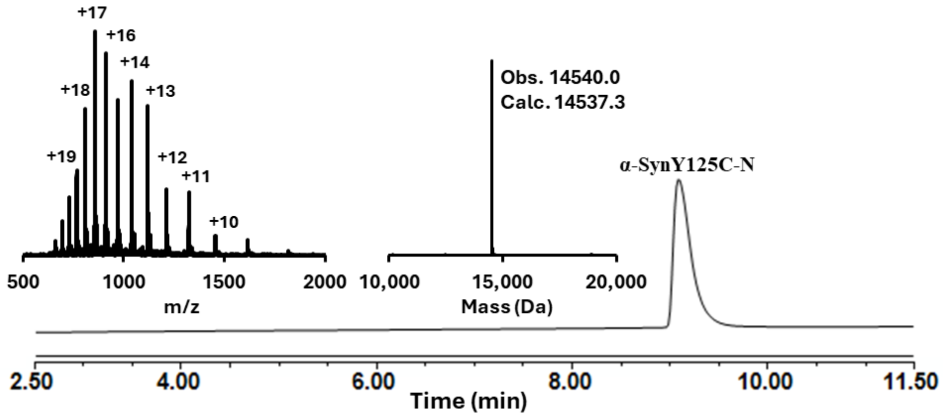

Figure S20. LC-MS analysis of isolated α-SynY125C-N. LC chromatogram of the UV absorbance at 214 nm, mass-to-charge (m/z) spectrum and deconvoluted mass spectrum. LC-MS analysis was carried out as described in section 1.3.

9.2.3 α-SynY133C nitration with Pd-1

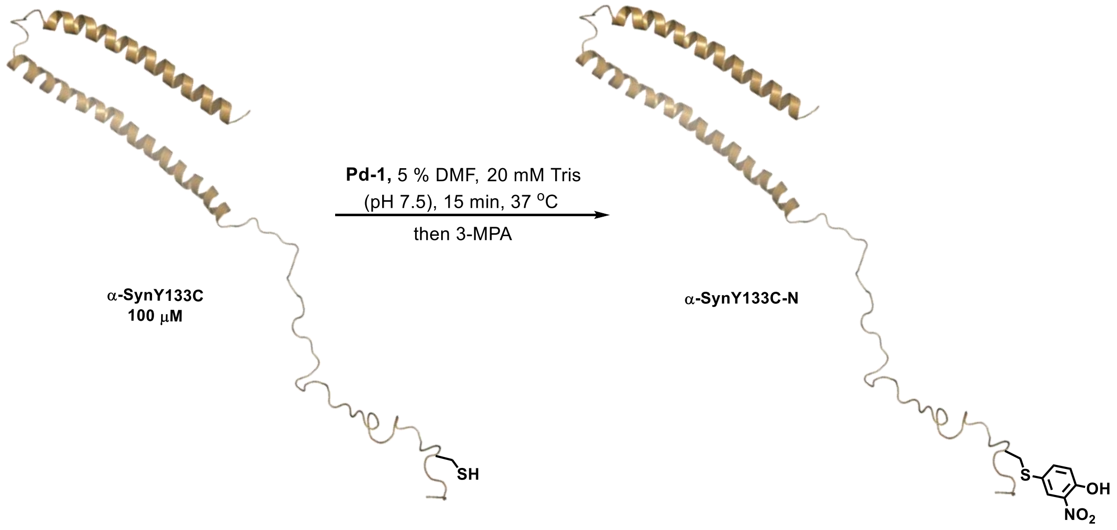

To a 15 mL falcon tube was added **α-SynY133C** (4.0 mg, 2.64 mL, 105.2 µM, 1.0 equiv.) in 20 mM Tris (pH 7.5), and the solution was preheated at 37 °C for 1 min. Then **Pd-1** (2.2 mg, 139 µL, 20.0 mM, 10.0 equiv.) as a solution in DMF (v = 5 %) was added in one portion. The reaction mixture was vigorously stirred for a few seconds and then incubated at 37 °C for 15 min. The final reaction concentrations of the major reaction components were as follows: **α-SynY133C** (100 µM); Pd-**1** (1 mM). Then, 3-MPA (0.5 µL, 20 equiv. compared with **α-SynY133C**) was added and the reaction mixture was kept at 37 °C for 15 min to quench the reaction. The reaction mixture was purified by RP-HPLC using method A (as described in section 1.5) affording the desired product **α-SynY133C-N** 2.2 mg. (55%) as a white powder.

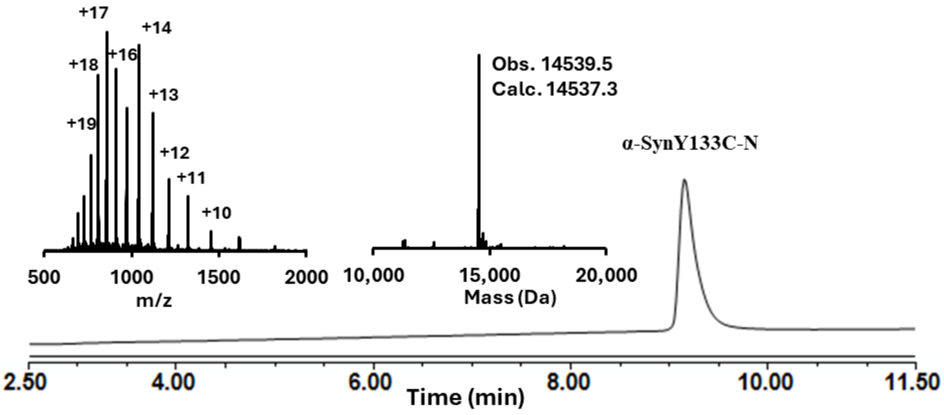

Figure S21. LC-MS analysis of isolated α-SynY133C-N. LC chromatogram of the UV absorbance at 214 nm, mass-to-charge (m/z) spectrum, and deconvoluted mass spectrum. LC-MS analysis was carried out as described in section 1.3.

9.2.4 α-SynY136C nitration with Pd-1

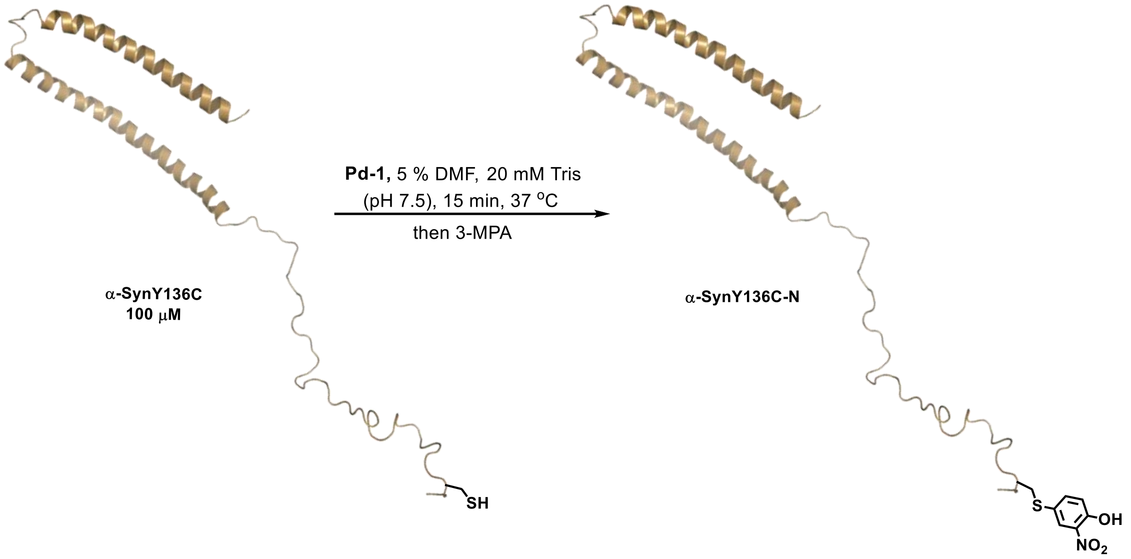

To a 15 mL falcon tube was added **α-SynY136C** (4.0 mg, 2.64 mL, 105.2 µM, 1.0 equiv.) in 20 mM Tris (pH 7.5), and the solution was preheated at 37 °C for 1 min. Then **Pd-1** (2.2 mg, 139 µL, 20.0 mM, 10.0 equiv.) as a solution in DMF (v = 5%) was added in one portion. The reaction mixture was vigorously stirred and then incubated at 37 °C for 15 min. The final reaction concentrations of the major reaction components were as follows: **α-SynY136C** (100 µM); **Pd-1** (1 mM). Then, 3-MPA (0.5 µL, 20 equiv. compared with **α-SynY136C**) was added and the reaction mixture was kept at 37 °C for 15 min to quench the reaction. The reaction mixture was purified by RP-HPLC using method C (as described in section 1.5) affording the desired product **α-SynY136C-N** 1.2 mg. (30%) as a white powder.

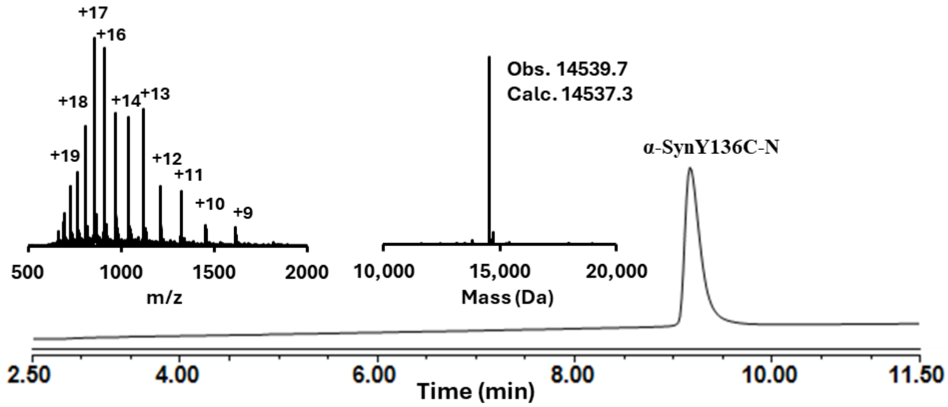

Figure S22. LC-MS analysis of isolated α-SynY136C-N. LC chromatogram of the UV absorbance at 214 nm, mass-to-charge (m/z) spectrum and deconvoluted mass spectrum. LC-MS analysis was carried out as described in section 1.3.

10. Preparation & characterization of recombinant monomeric α-Syn species

BL21 (DE3) cells transformed with the pT7-7 plasmid encoding the WT α-syn or Y39C or Y125C or Y133C or Y136C α-syn mutants were grown in LB medium at 37°C and induced with 1 mM 1-thio-β-d-galactopyranoside (AppliChem) at an O.D. between 0.4–0.6 and continued to grow for 4 h at 37°C. Following the overnight incubation at 18°C, induced bacterial cultures were pelleted and sonicated for the lysis of the cells. After centrifugation at 18,000 g for 20 min, the supernatant was boiled for 5 min and centrifuged again for 20 min. The supernatant was purified by anion exchange chromatography (HiPrep 16/10 Q FF, GE Healthcare Life Sciences), followed by reverse-phase HPLC (Jupiter 300 C4, 20 mm I.D. × 250 mm, 10 μm average bead diameter, Phenomenex) and lyophilized.

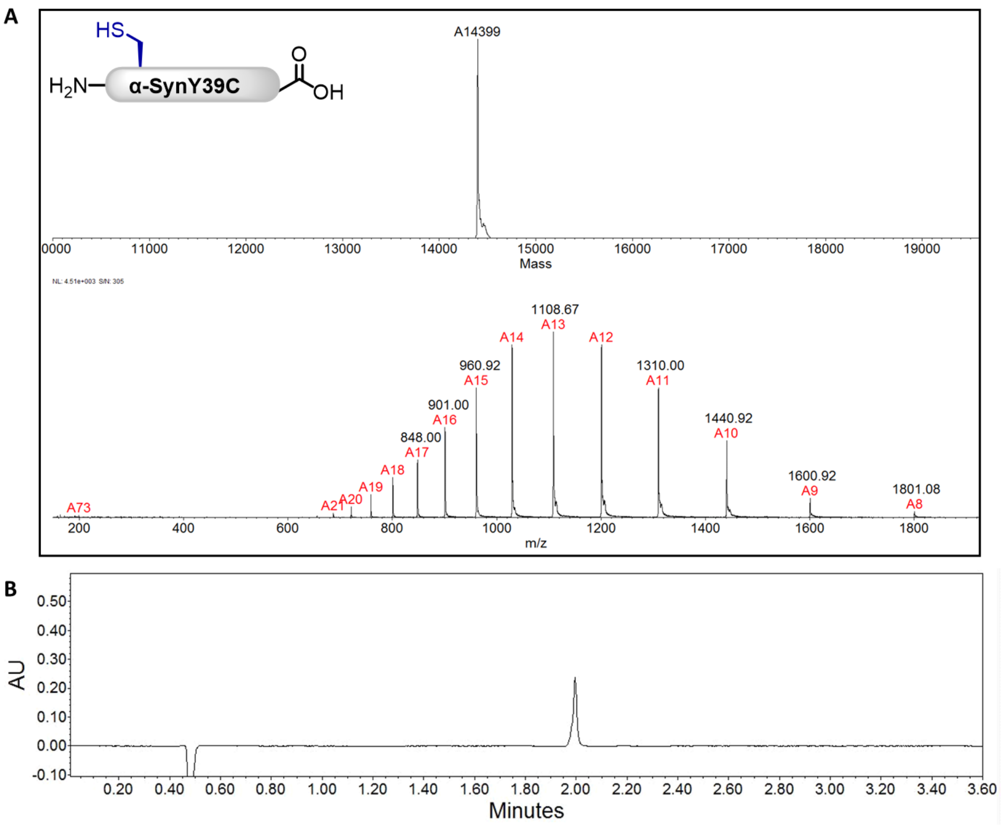

Figure S23. A. MS spectrum of purified **α-SynY39C**. Calculated mass = 14400 Da, observed mass = 14399 Da. B. Reversed-phase UHPLC.

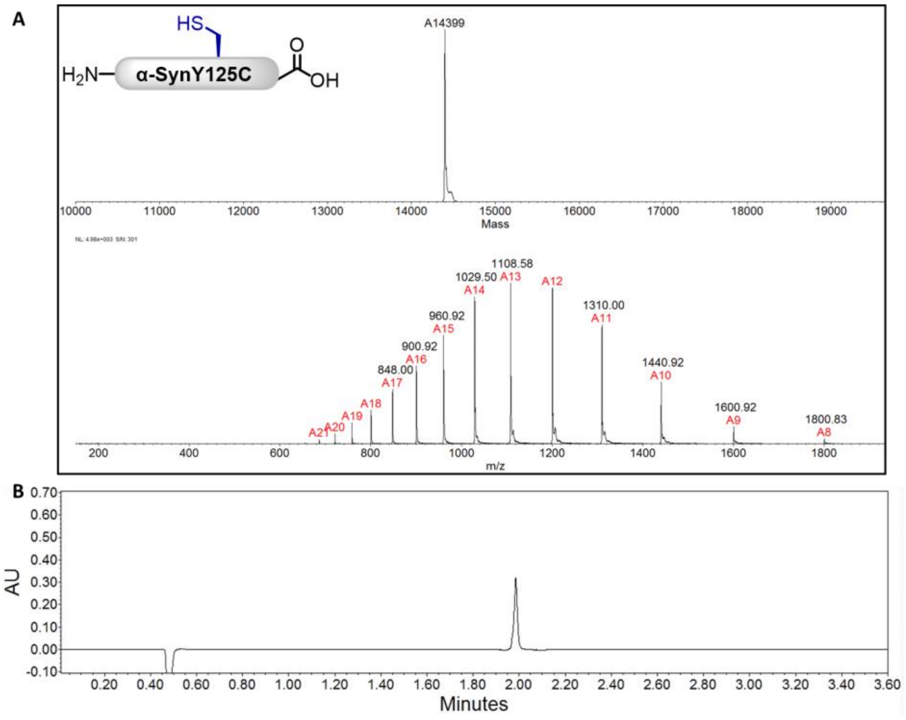

Figure S24. A: MS spectrum of purified **α-SynY125C**. Calculated mass = 14400 Da, observed mass = 14399 Da. B. Reversed-phase UHPLC.

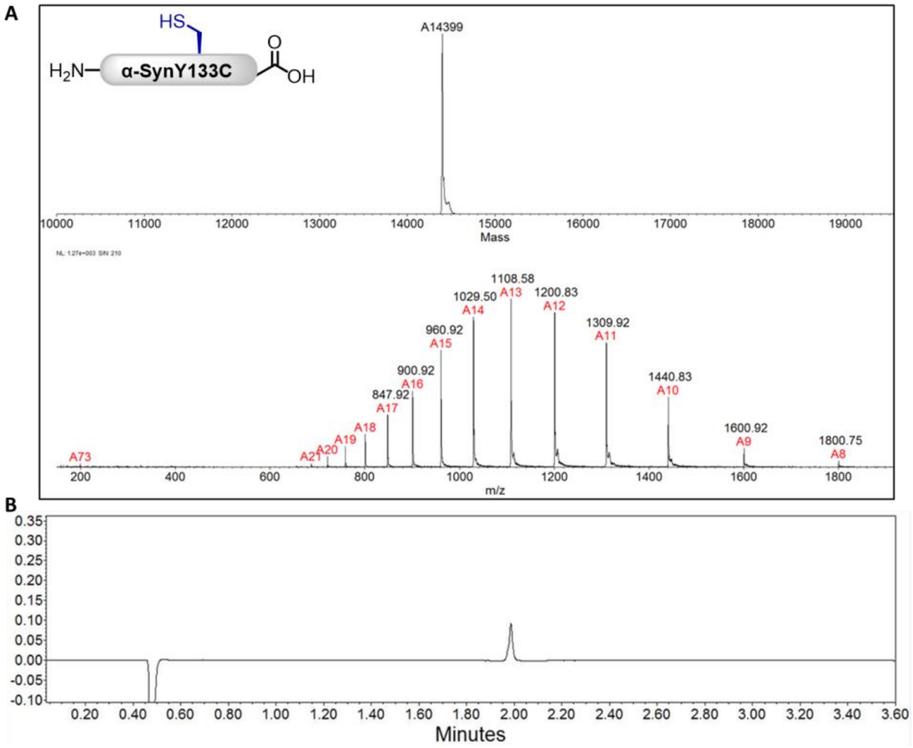

Figure S25. A. MS spectrum of purified **α-SynY133C**. Calculated mass = 14400 Da, observed mass = 14399 Da. B. Reversed-phase UHPLC.

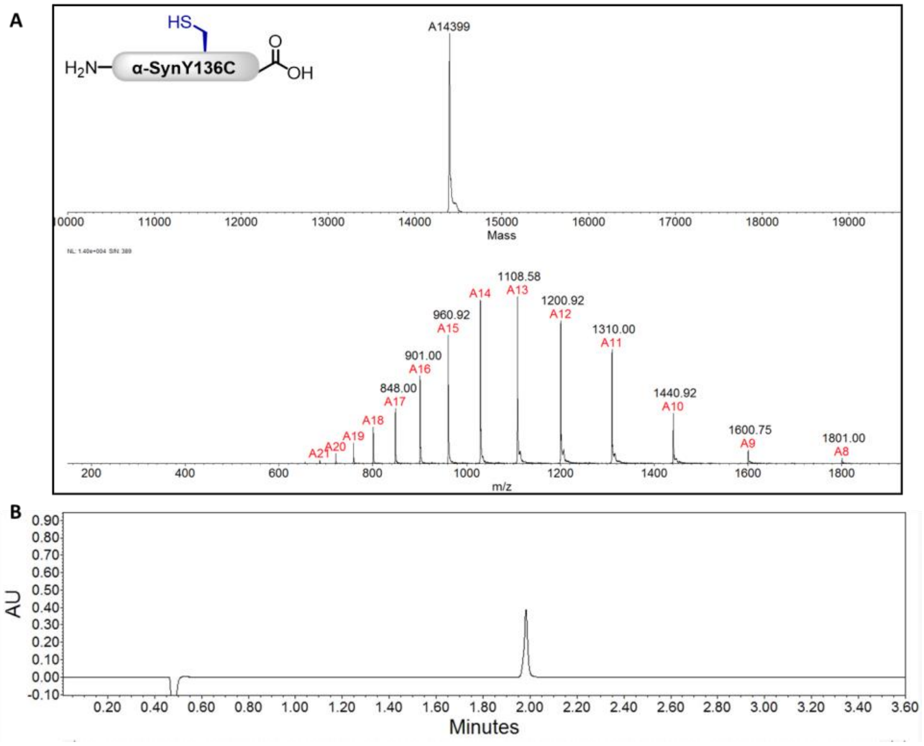

Figure S26. A. MS spectrum of purified **α-SynY136C**. Calculated mass = 14400 Da, observed mass = 14399 Da. B. Reversed-phase UHPLC.

11. SDS-PAGE analysis

Samples for SDS-PAGE were mixed with 2 × Laemmli buffer (4% SDS, 20% glycerol, 0.004% bromphenol blue, 0.125 M Tris-Cl, 10% 2-mercaptoethanol pH 6.8) and loaded onto 16% polyacrylamide tricine gels. The gel was run at 120 V for 2 hr in running buffer (25 mM Tris, 192 mM Glycine, 0.1% SDS, pH 8.3), followed by staining with a solution of 25% (v/v) isopropanol, 10% acetic acid (v/v) and 0.05% (w/v) Coomassie brilliant blue R (Applichem) or the commercial brilliant blue and destaining with boiling distilled water.

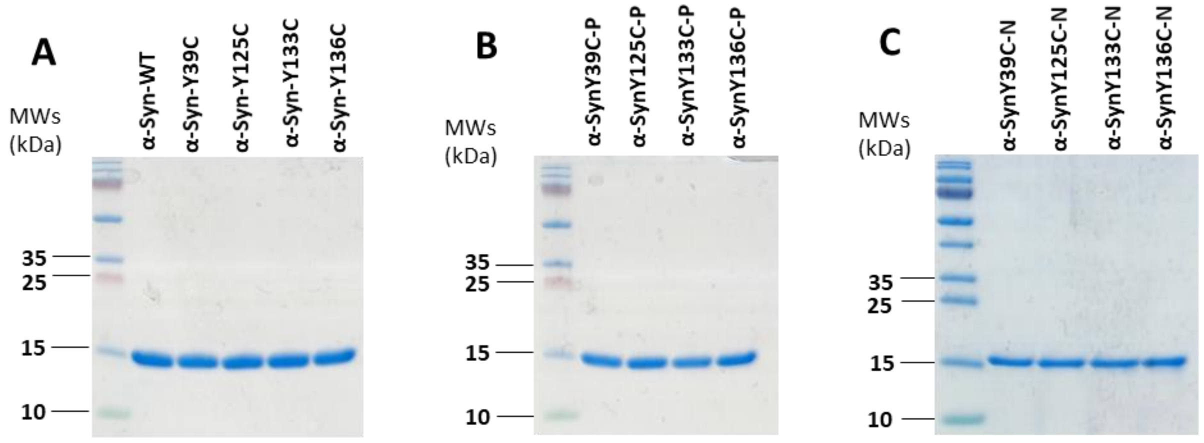

Figure S27. SDS-PAGE for A. **WT α-Syn**, **α-SynY39C, α-SynY125C, α-SynY133C, α-SynY136C** proteins; B**. α-SynY39C-P, α-SynY125C-P, α-SynY133C-P**, and **α-SynY136C-P**; C. **α-SynY39C-N, α-SynY125C-N, α-SynY133C-N**, and **α-SynY136C-N**.

12. Circular dichroism spectroscopy

Circular dichroism (CD) spectroscopy was performed at room temperature on a Jasco J-815 spectrometer. Protein samples (200 µL, 20 µM) in PBS buffer (pH 7.4) were placed in a 1 mm optical path quartz cuvette for analysis. The spectra were obtained by averaging four scans per sample in a fused quartz cell over the 280 to 195 nm region at a step of 1nm (scan rate=1 sec/step). The processed spectra were obtained by subtracting the baseline signal from the protein spectra without further smoothing. The raw data were converted to molar ellipticity per residue.

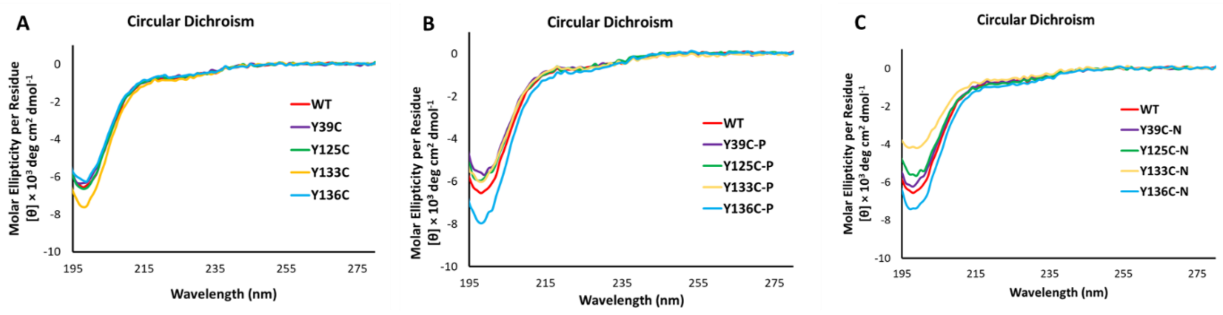

Figure S28. CD spectra of A. expressed WT and mutants; B. phosphorylated proteins and C. nitrated proteins.

13. Transmission electron microscopy (TEM)

Before the application of the sample, Formvar and carbon-coated 200 mesh-containing copper EM grids (Electron Microscopy Sciences) were glow-discharged for 30 s at 20 mA using a PELCOeasiGlow™ Glow Discharge Cleaning System (TED PELLA, Inc). Subsequently, 5 µL of the sample was placed onto the EM grid for a minute. Then, the sample was carefully blotted using filter paper and air-dried for 30 s. Then, the grids were washed three times with ultrapure water and stained with 1% (w/v) uranyl formate solution. Grids were examined using a Bio Talos electron microscope. The microscope was equipped with a LaB6 gun operated at an acceleration voltage of 80 kV, and images were captured using a 4K × 4K charge-coupled device camera (FEI Eagle). The length (ln) or diameter (d) of respective α-syn oligomeric fractions was measured using ImageJ software (NIH).

14. ThT Aggregation Kinetics Assay

Lyophilized WT, Cys mutants, phosphorylated, and nitrated proteins were dissolved to about 30 µM in PBS buffer (pH 7.4). Protein solutions were then filtered through 100 kDa MWCO Microcon filters to remove any insoluble material or oligomers at the beginning of the experiment. Protein concentrations were precisely measured by UV spectroscopy (using extinction coefficients of ε280nm = 5960 M-1cm-1 for wild-type α-Syn and ε280nm = 4470 M-1cm-1 for mutant and modified α-Syn). Proteins were then diluted to a final concentration of 15 or 20 µM (final volume: 400 µL). A fresh ThT solution (0.01 mM ThT solution in 0.5M glycine, pH 8.5) was added to each (a total of 13) protein samples, and 100 µL solution was added to a clear bottom 96 well plate (Corning). The plate was then sealed with a clear film. Aggregations were conducted in the FLUstar Omega microplate reader (BMG Labtech Germany) at an excitation wavelength of 450 nm and an emission wavelength of 485 nm. The reading was taken from time 0 to every 8 min for the next 3 days with ideal shaking at 37°C. Three independent experiments were performed in triplicates for each protein.

ThT Aggregation kinetics of Cys-mutants and TEM characterization of the aggregates

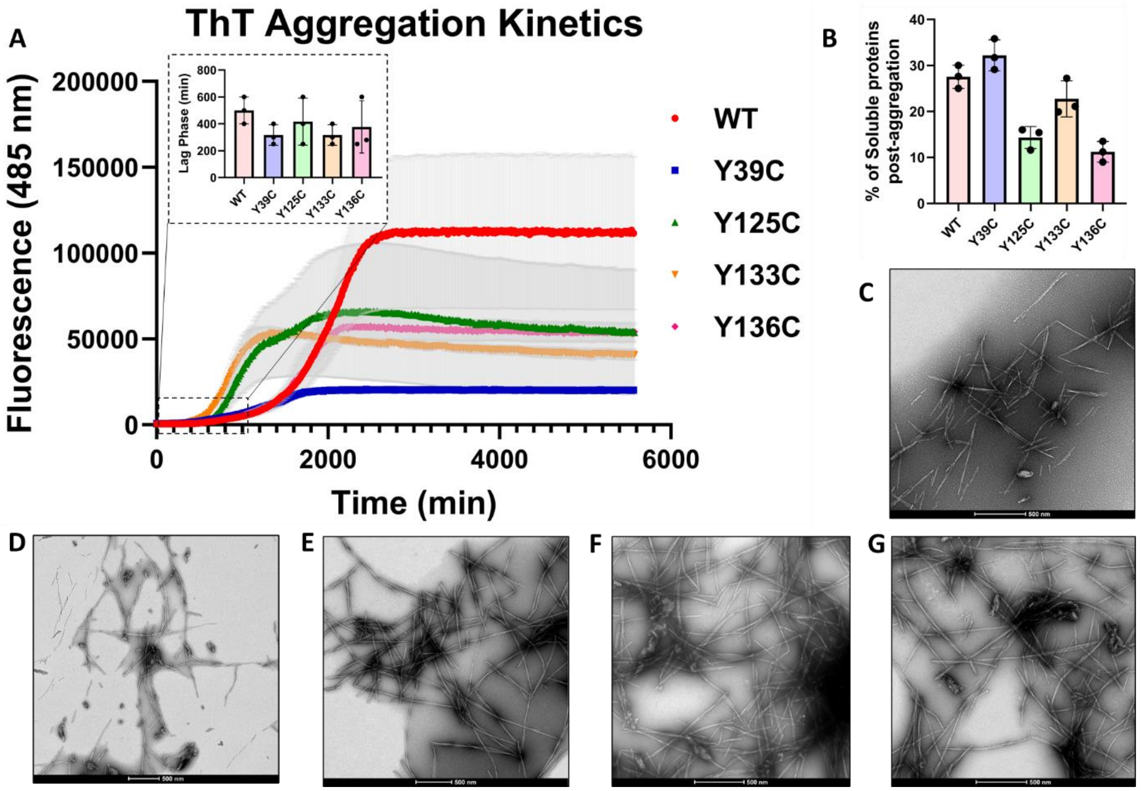

Figure S29. A. Aggregation kinetics of recombinant **WT α-Syn, α-SynY39C, α-SynY125C, α-SynY133C** and **α-SynY136C,** based on thioflavin T fluorescence (ThT ±SD; n=3). The lag phase has been shown inside the box; B. Corresponding quantification of remaining soluble protein ± SEM of recombinant **WT α-Syn, α-SynY39C, α-SynY125C, α-SynY133C** and **α-SynY136C** (n=3); Electron micrographs of recombinant C. **WT α-Syn**; D**. α-Syn Y39C**; E. **α-SynY125C**; F. **α-SynY133C**; and G. **α-SynY136C**. The scale bars are 500 nm.

Comparison of aggregation kinetics via Thioflavin T assay among α-Syn-Y39/125/133/136C vs. α-Syn-Y39/125/133/136C-P vs. α-Syn-Y39/125/133/136C-N analogues

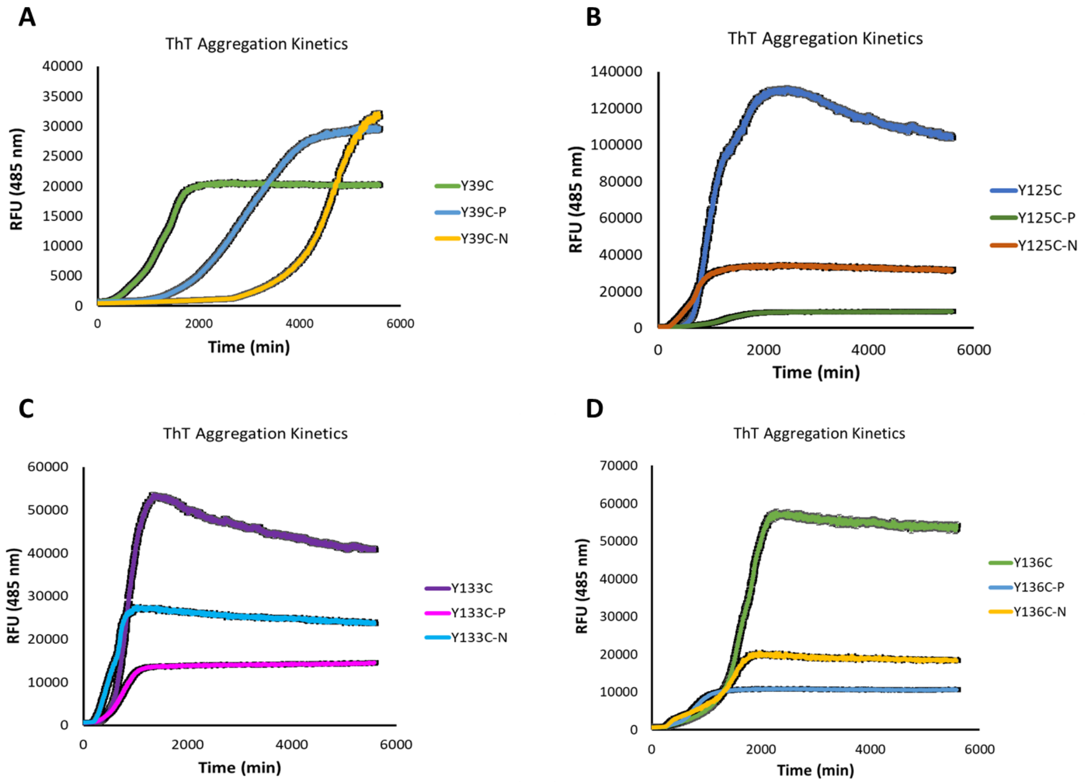

Figure S30. ThT aggregation kinetics comparison among α-Syn analogues, A. Y39C, Y39C-P & Y39C-N analogues; B. Y125C, Y125C-P & Y125C-N analogues; (c) Y133C, Y133C-P & Y133C-N analogues; (d) Y136C, Y136C-P & Y136C-N analogues.

15. Sedimentation assay

Our well-established centrifugation-based assay can separate sedimentable fibrils from the monomers and oligomers. First, 10 µL of the sample after aggregation was taken out and diluted with 2X LB. This is represented as "total". After, a total sample volume of 50 µL was subjected to ultracentrifugation at 100,000 g for 30 min at 4°C. Following centrifugation, careful pipetting of the supernatant from the pellet is carried out to isolate the soluble species, which represents the monomers & oligomers (supernatant). The isolated pellet was resuspended in 50 µL PBS and represented as Fibrils (pellet). These three fractions from all 13 protein samples were run in hand-made 16% tricine gels on 120V for 2.5h. After the separation, the gels were incubated with instant blue Coomassie solution at 37 degrees overnight on a shaker and destained with distilled water washes (X3) at 37 degrees for 1h to visualize the distribution.

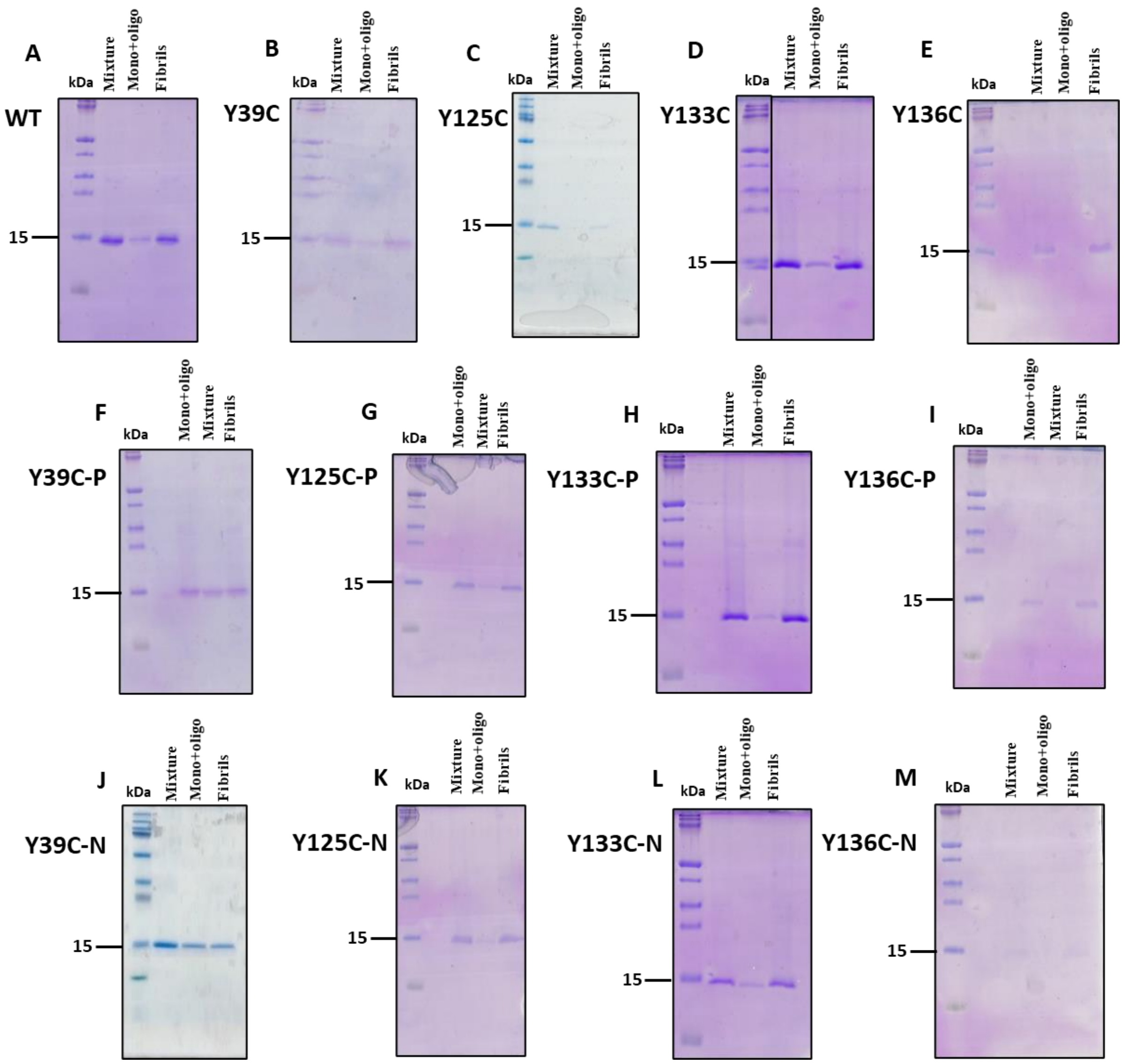

Figure S31. The gels are quantitative representations of the soluble protein forms collected from the supernatant (monomers and fibrils) from the fibrils in pellet. The combination of both supernatant and pellet gives the total mixture. (A-E) WT, Y39C, Y125C, Y133C & Y136C α-Syn respectively. (F-I) Y39C-P, Y125C-P, Y133C-P, & Y136C-P α-Syn respectively. (J-M) Y39C-N, Y125C-N, Y133C-N, & Y136C-N α-Syn respectively.

Zoomed TEM images of phosphorylated α-Syn

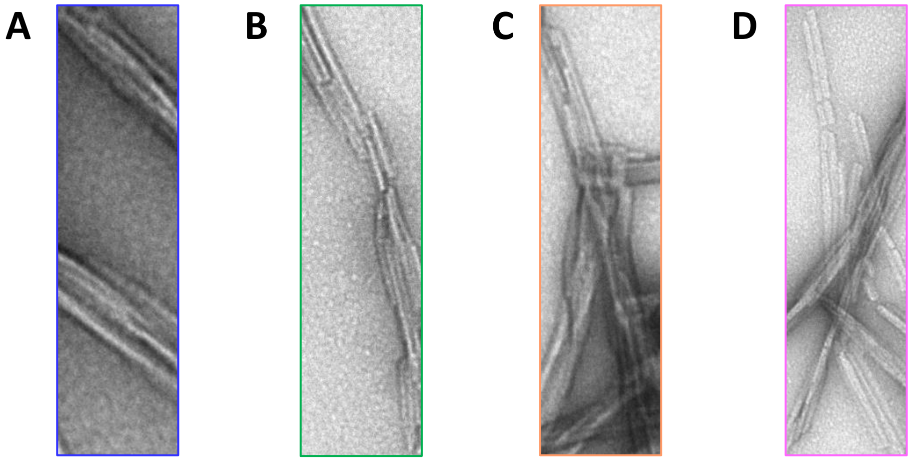

Figure S32. TEM images of A. **α-SynY39C-P**; B. **α-SynY125C-P**; C. **α-SynY133C-P** and D. **α-SynY136C-P**.

Comparison of fibril width of the phosphorylated analogs with WT α-Syn.

The width (nm) of the fibrils was calculated on FiJi software.

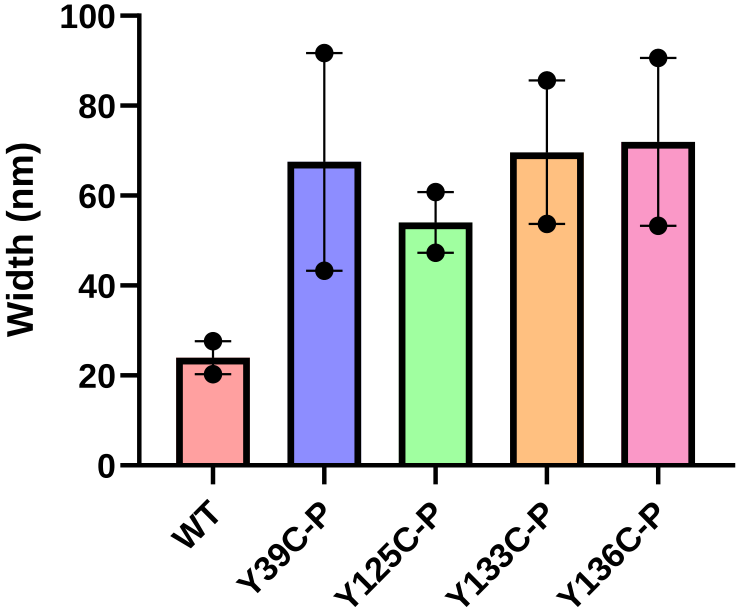

Figure S33. The diameter of the stacked fibrils from WT α-Syn, α-SynY39C-P, α-SynY125C-P, α-SynY133C-P, α-SynY136C-P analogs. Error bars are standard errors of the mean.

Primary Antibody	**Name**	**Species**	**Epitope**	**Manufacturer (Cat No.)**	**Dilution**
	Anti-α-Syn (SYN-1) Code : B59	Mouse Monoclonal	91-99	BD Transduction (BD610787)	1:1000
	Anti-nitrated α-Syn Code: B199	Mouse Monoclonal	Nitrated aSyn	Virginia Lee's group	1:1000
	Anti-nitrated α/β-Syn Code : B200	Mouse Monoclonal	nY125, nY136	Merck – Millipore (36-011)	1:1000
	Anti- α-Syn Code : B202	Mouse Monoclonal	nY125, nY133	Virginia Lee's group	1:1000
	Anti- α-Syn nY39 Code : B74	Rabbit Polyclonal	nY39	Eurogentec (LASH-EGT nY39 EC14003)	1:1000
	Anti α-Syn pY39 Code : B65	Rabbit Polyclonal	pY39	Eurogentec (LASH-EGT pY39 CH10039)	1:1000
	Anti α-Syn pY125 Code : B69	Rabbit Polyclonal	pY125	Eurogentec (LASH-EGT pY125 EC10003)	1:1000
	Anti α-Syn pY133 Code : B291	Rabbit Polyclonal	pY133	Abcam (ab194910)	1:1000
	Anti α-Syn pY136 Code : B292	Rabbit Polyclonal	134-138; pY136	Abcam (ab131491)	1:1000
Secondary Antibody	IRDye® 800CW Goat anti-Rabbit IgG (H + L)	-	-	LiCor (926-32210)	1:10000
	IRDye® 680RD Goat anti-Mouse IgG (H + L)	-	-	LiCor (926-68070)	1:10000

Table S1: Primary and secondary antibodies used in this study

Dot-Blot analysis. Nitrocellulose membranes (Amersham, 10,600,001) were spotted with 5 µL of 20 µM protein samples of α-syn species. The membranes were blocked for 1 h with Odyssey blocking buffer (LiCoR, 927-40000), and then incubated overnight with different primary antibodies (Table S1) diluted in PBS at 1:1000 dilution. The membranes were washed three times, with 0.1% PBS-Tween (3 × 10 minutes) and incubated with IR dye conjugated secondary antibodies (1:10000) (Table S1) for 1 h at RT. Thereafter, the membranes were washed three times, with 0.1% PBS-Tween (3 × 10 minutes). The visualization was performed by fluorescence using Odyssey CLx from LiCor.

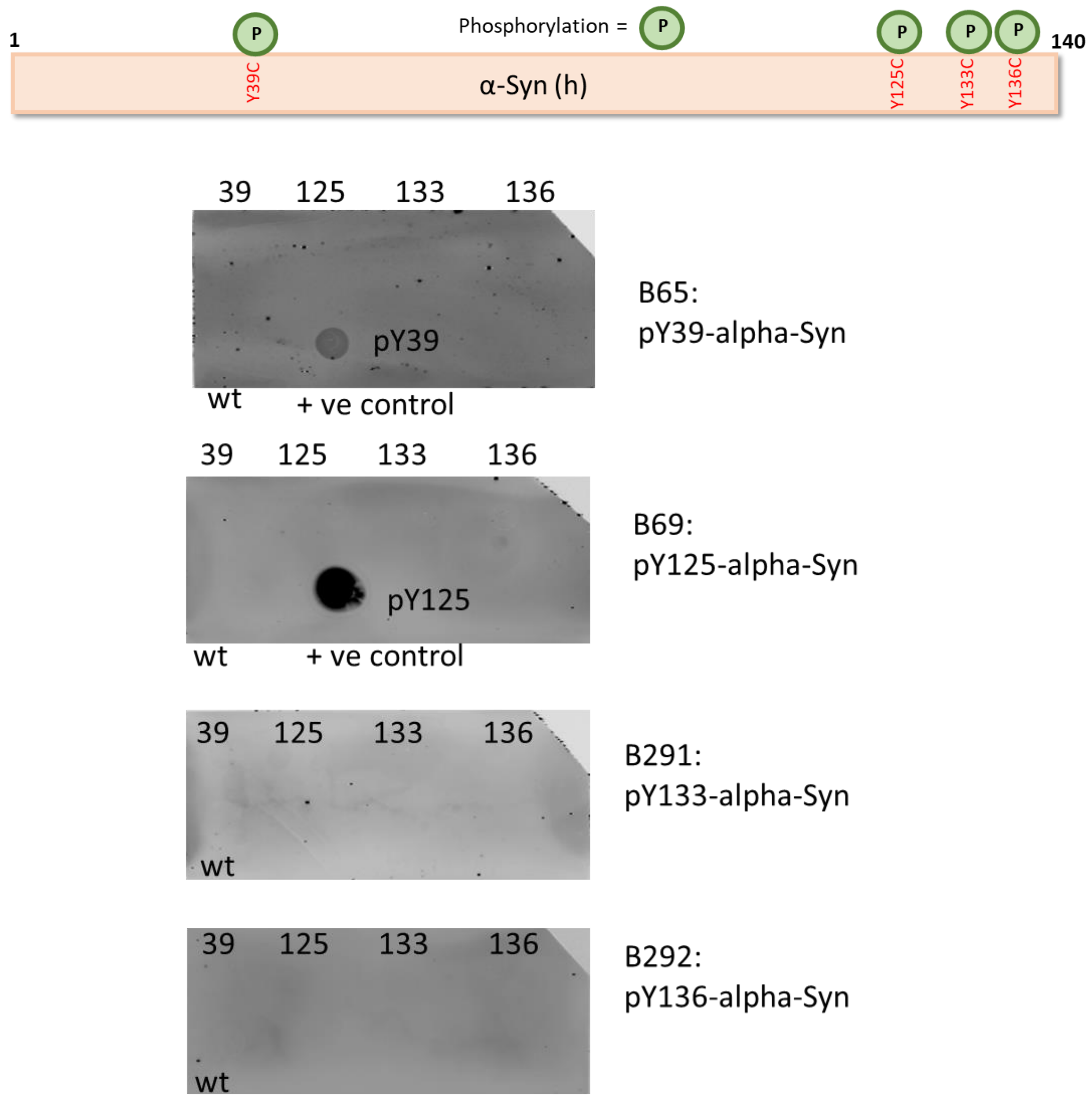

Figure S34. Dot-Blot of phosphorylated analogs (α-SynY39C-P, α-SynY125C-P, α-SynY133C-P, and α-SynY136C-P).

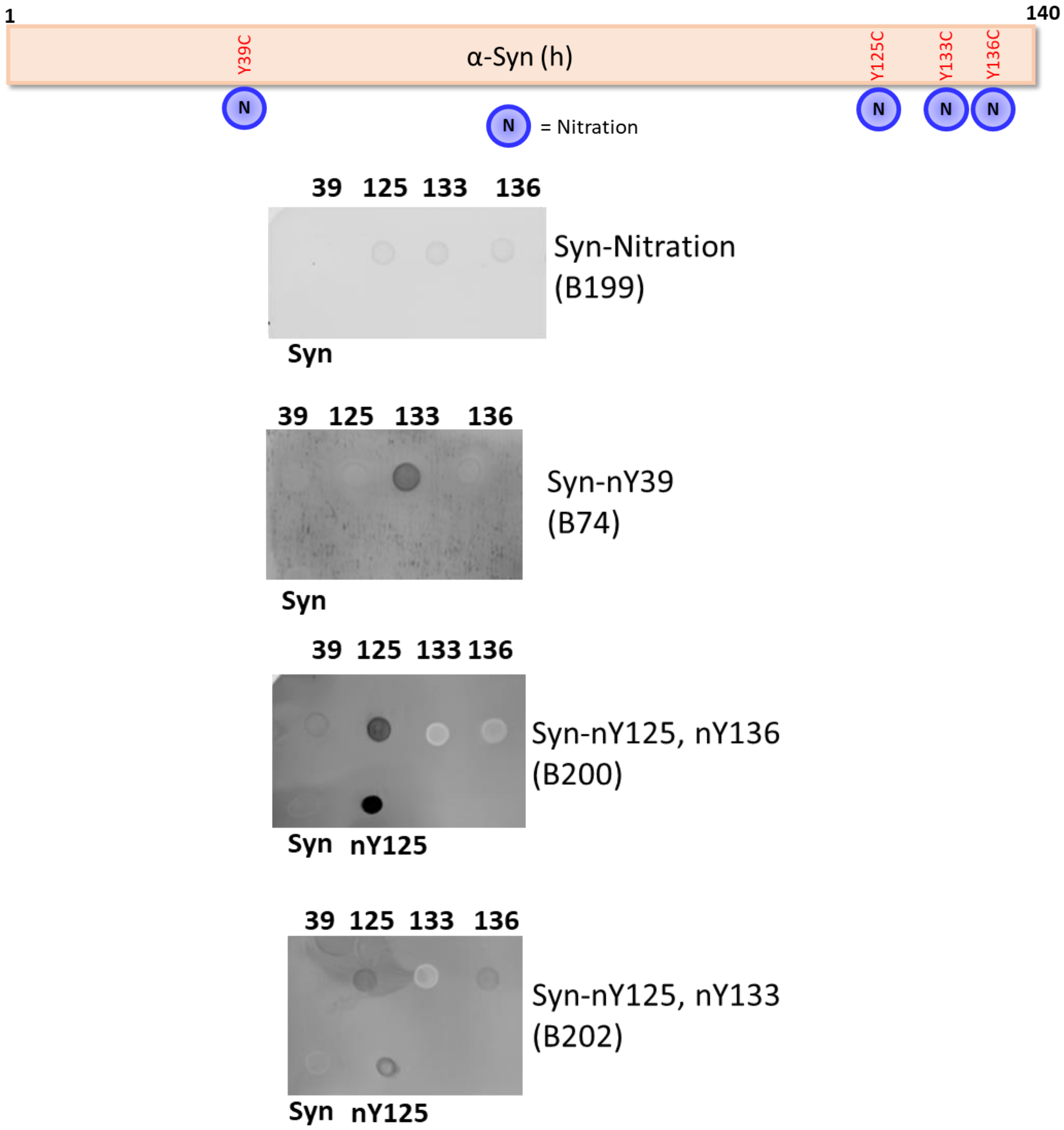

Figure S35. Dot-Blot of the nitrated analogs (α-SynY39C-N, α-SynY125C-N, α-SynY133C-N, and α-SynY136C-N).

16. NMR spectra of Pd-1 and Pd-2

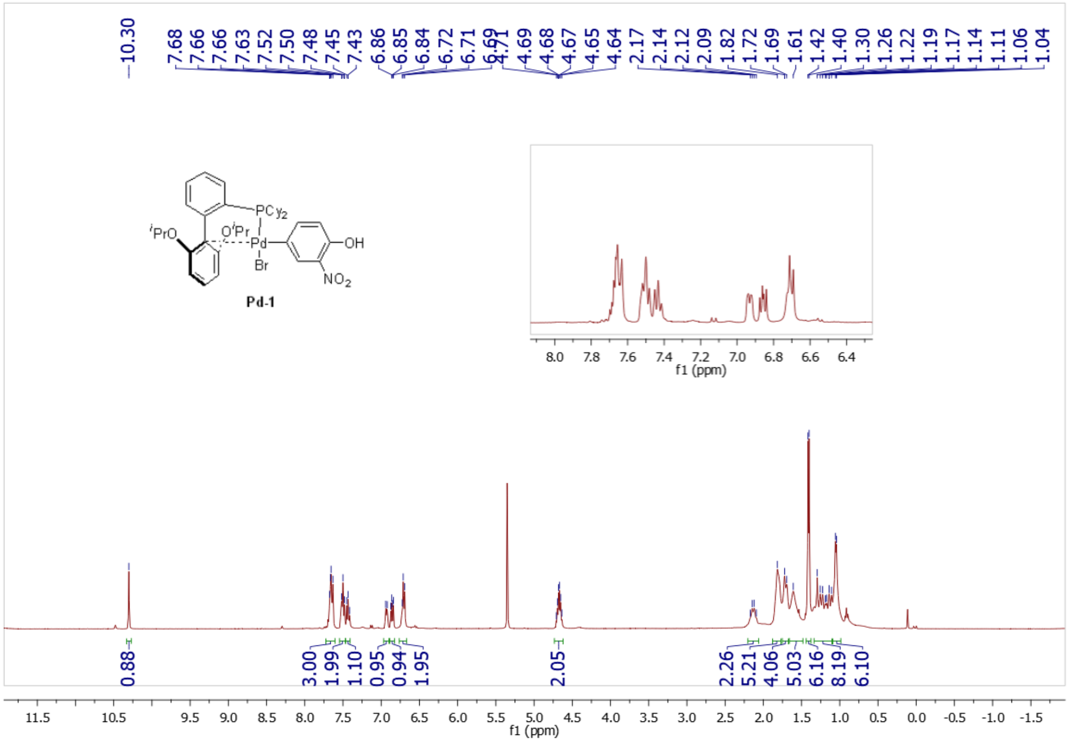

Figure S36. ^{1}H NMR spectrum of **Pd-1** in CD_2Cl_2.

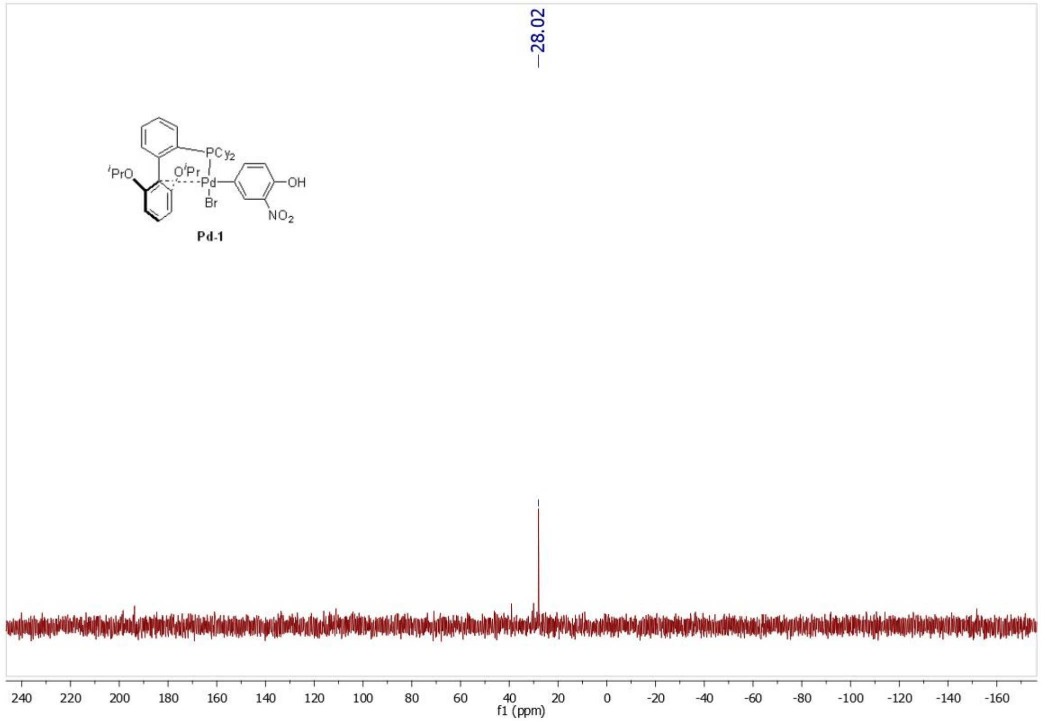

Figure S37. ^{31}P NMR spectrum of **Pd-1** in C_6D_6.

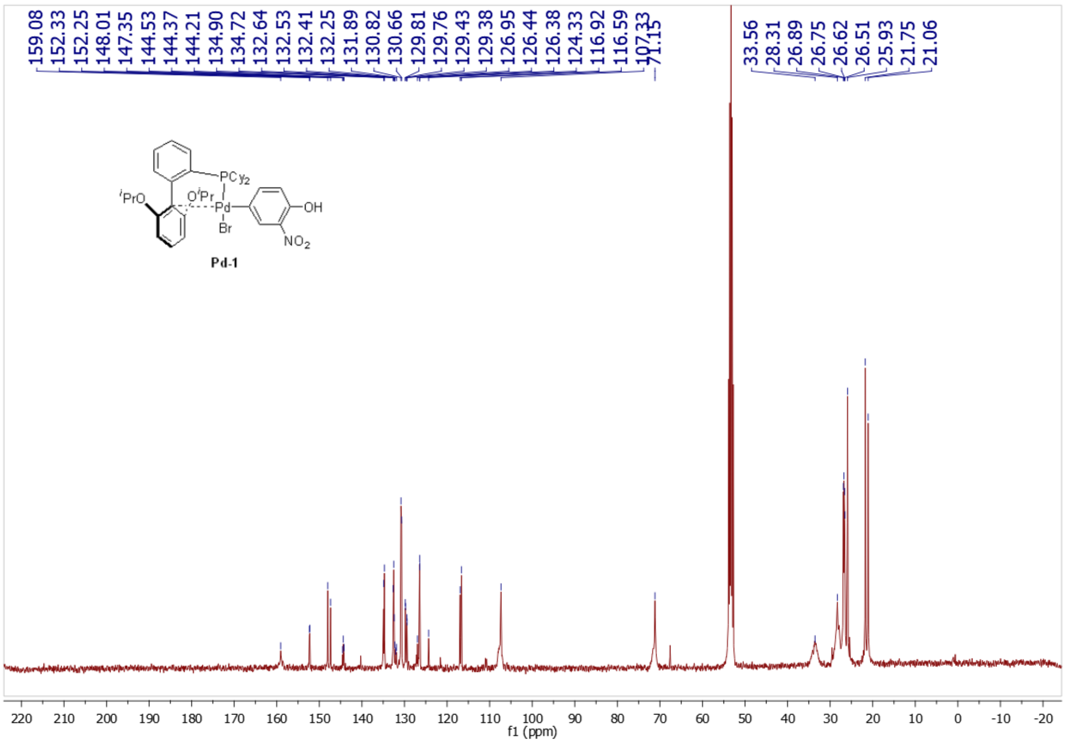

Figure S38. ^{13}C NMR spectrum of **Pd-1** in CD_2Cl_2.

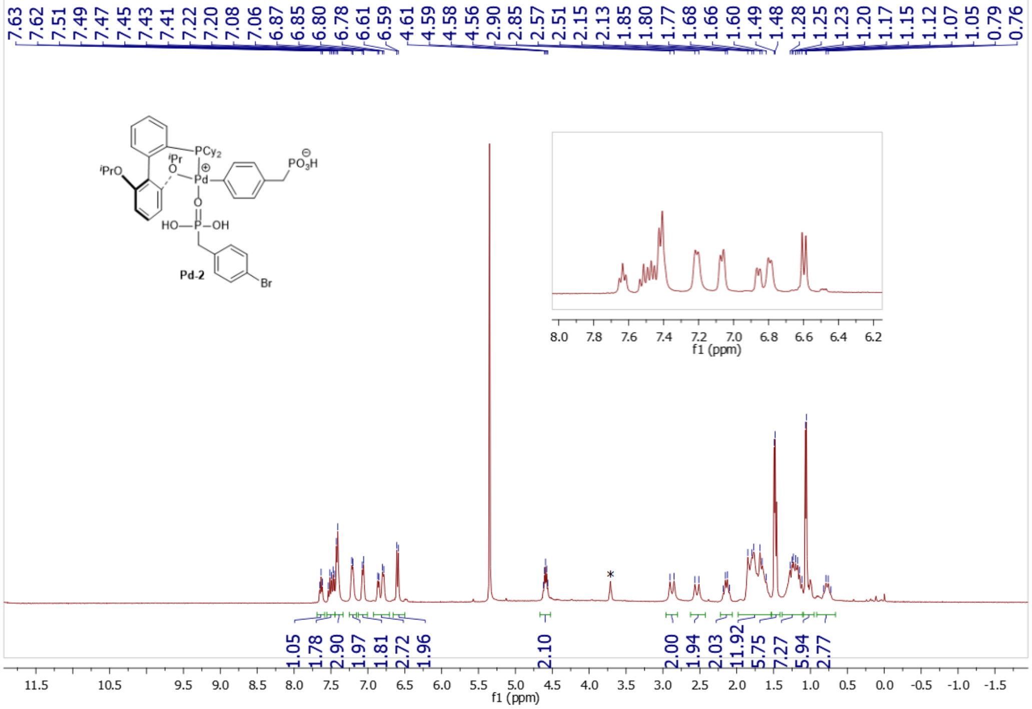

Figure S39. ^{1}H NMR spectrum of **Pd-2** in CD_2Cl_2. *THF

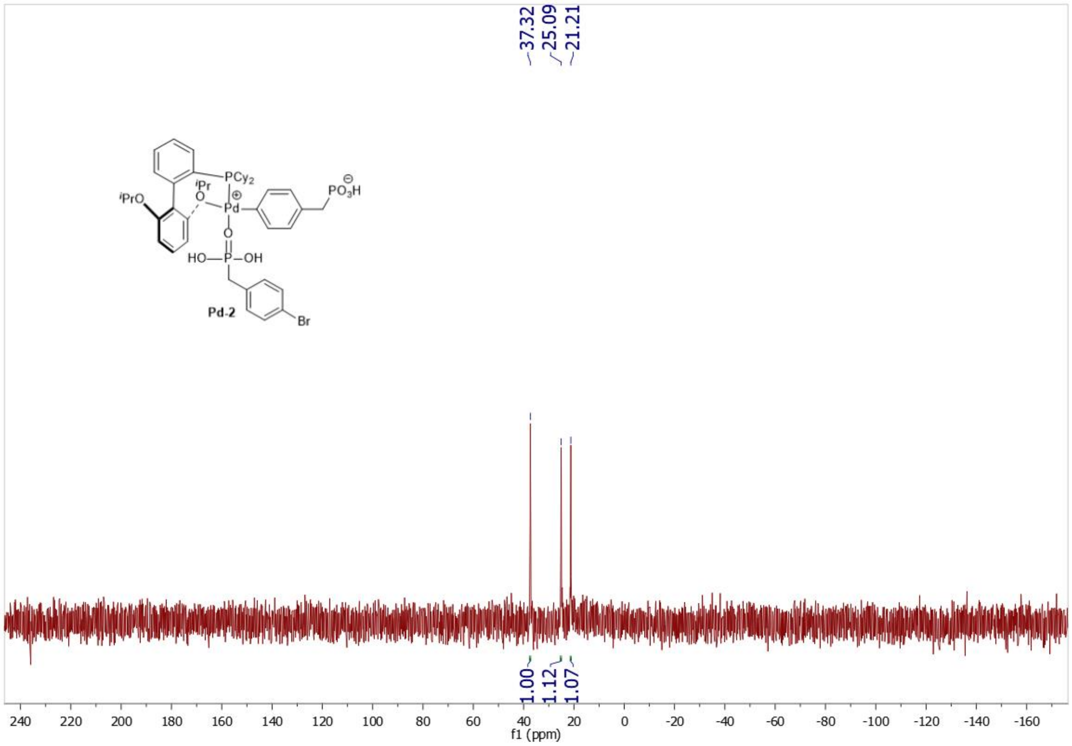

Figure S40. ^{31}P NMR spectrum of **Pd-2** in CD_2Cl_2.

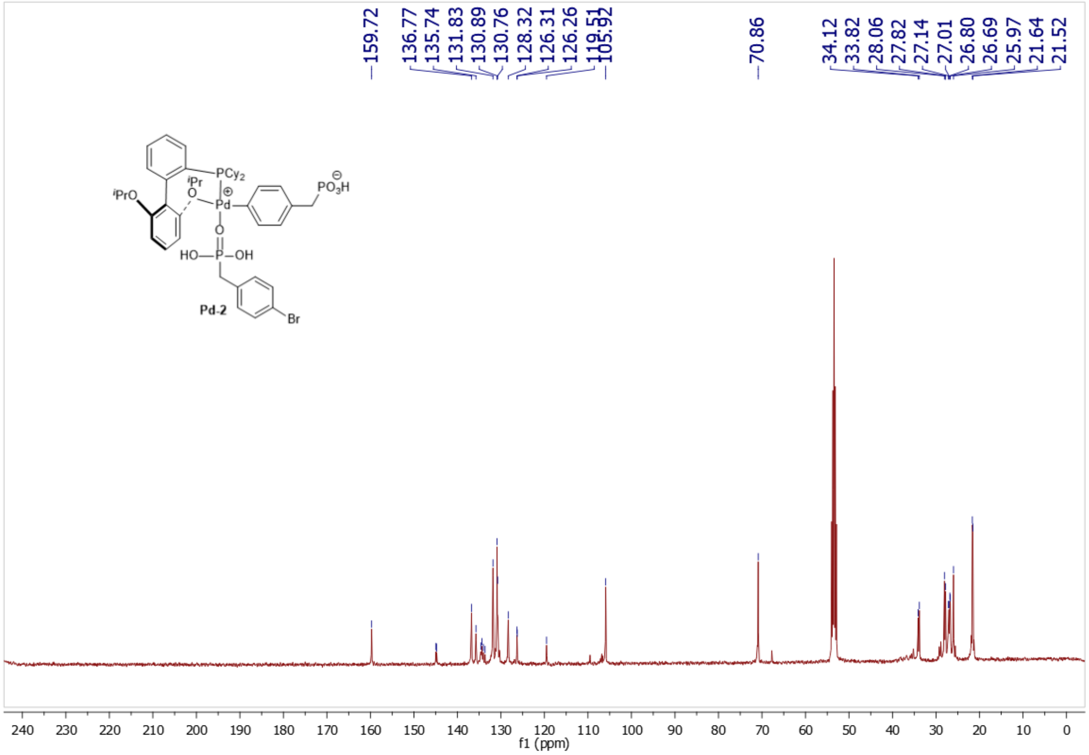

Figure S41. ^{13}C NMR spectrum of **Pd-2** in CD_2Cl_2.

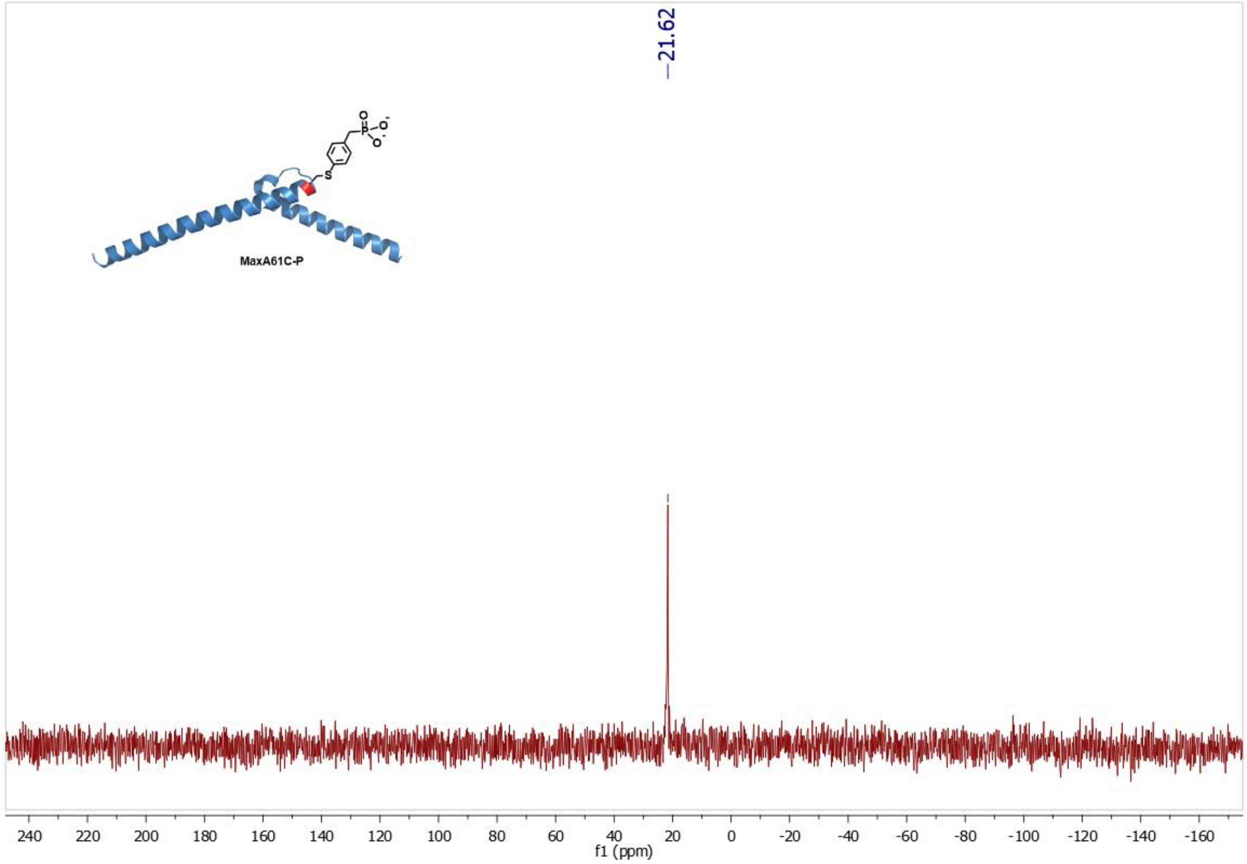

Figure S42. ^{31}P NMR spectrum of **MaxA61C-P** in D_2O.

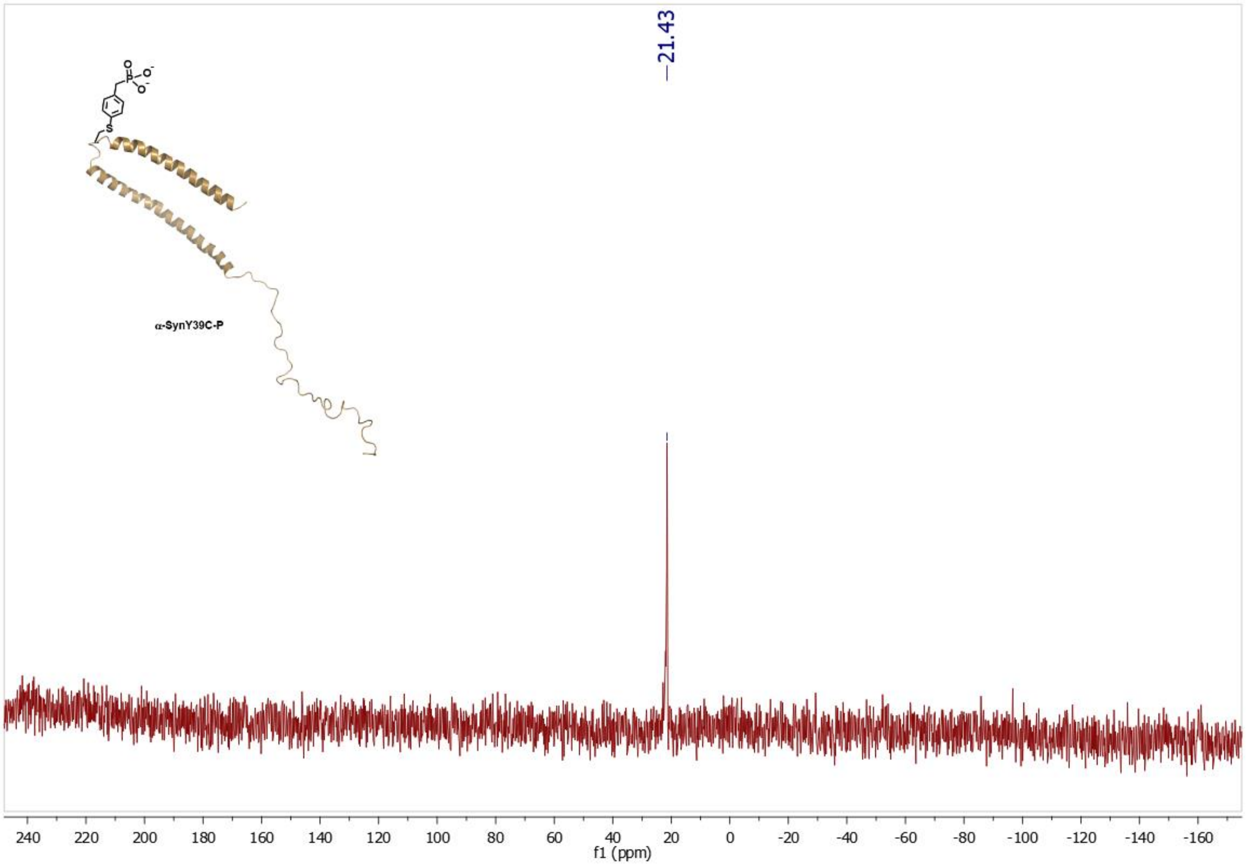

Figure S43. ^{31}P NMR spectrum of **α-SynY39C-P** in D_2O.

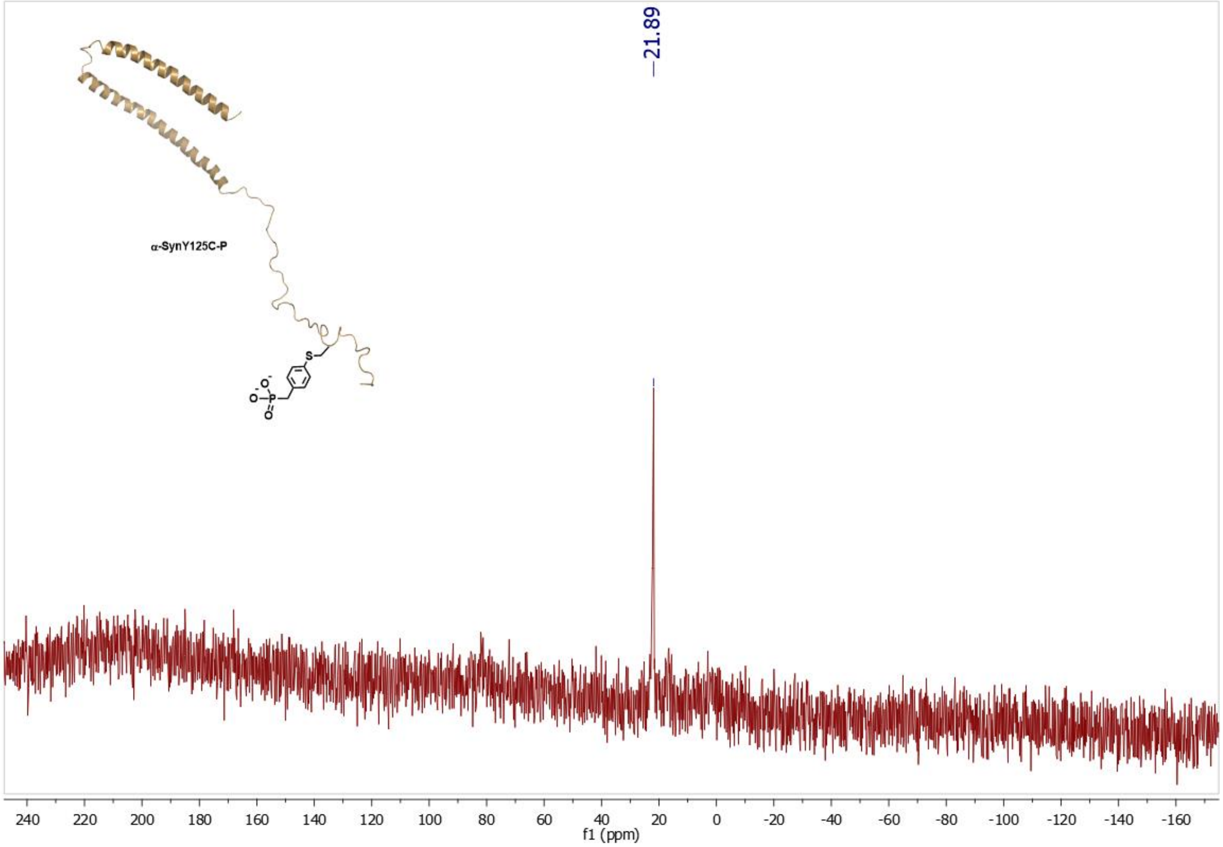

Figure S44. ^{31}P NMR spectrum of **α-SynY125C-P** in D_2O.

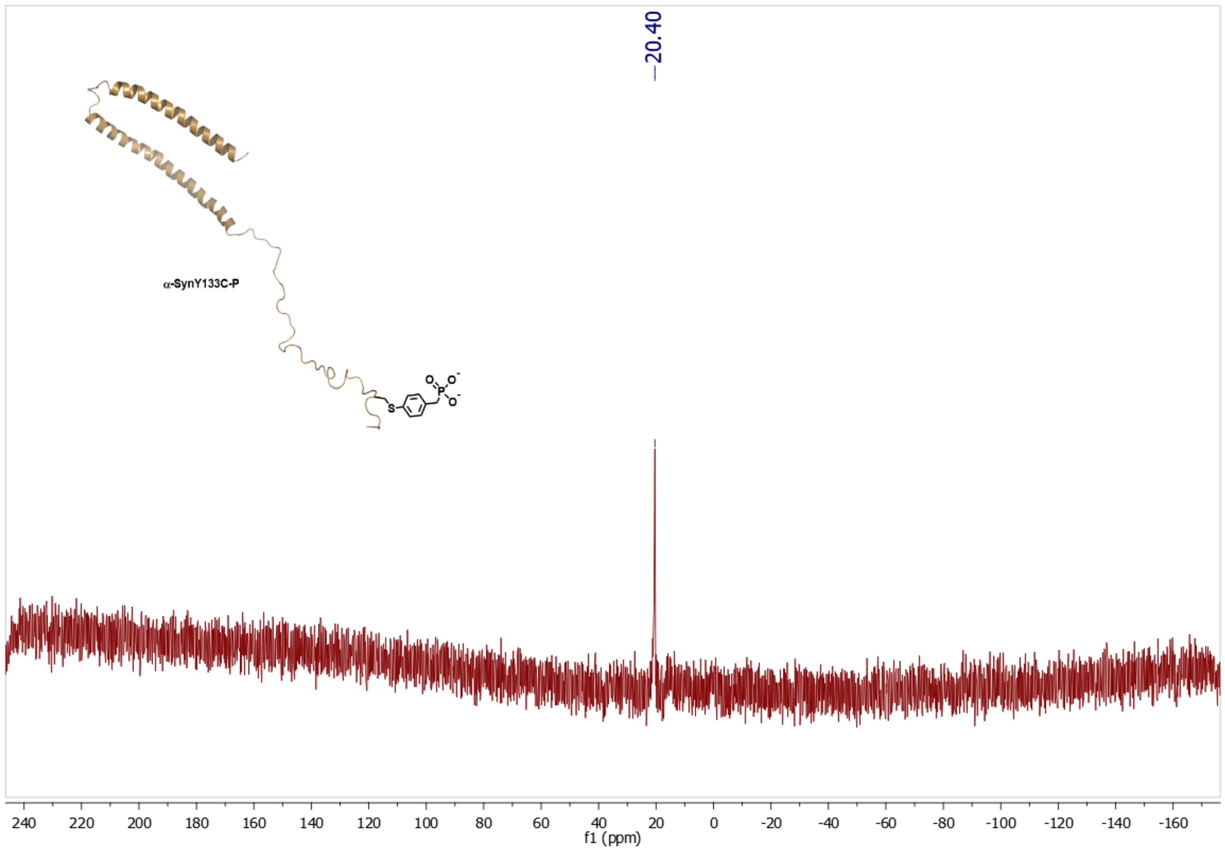

Figure S45. ^{31}P NMR spectrum of **α-SynY133C-P** in D_2O.

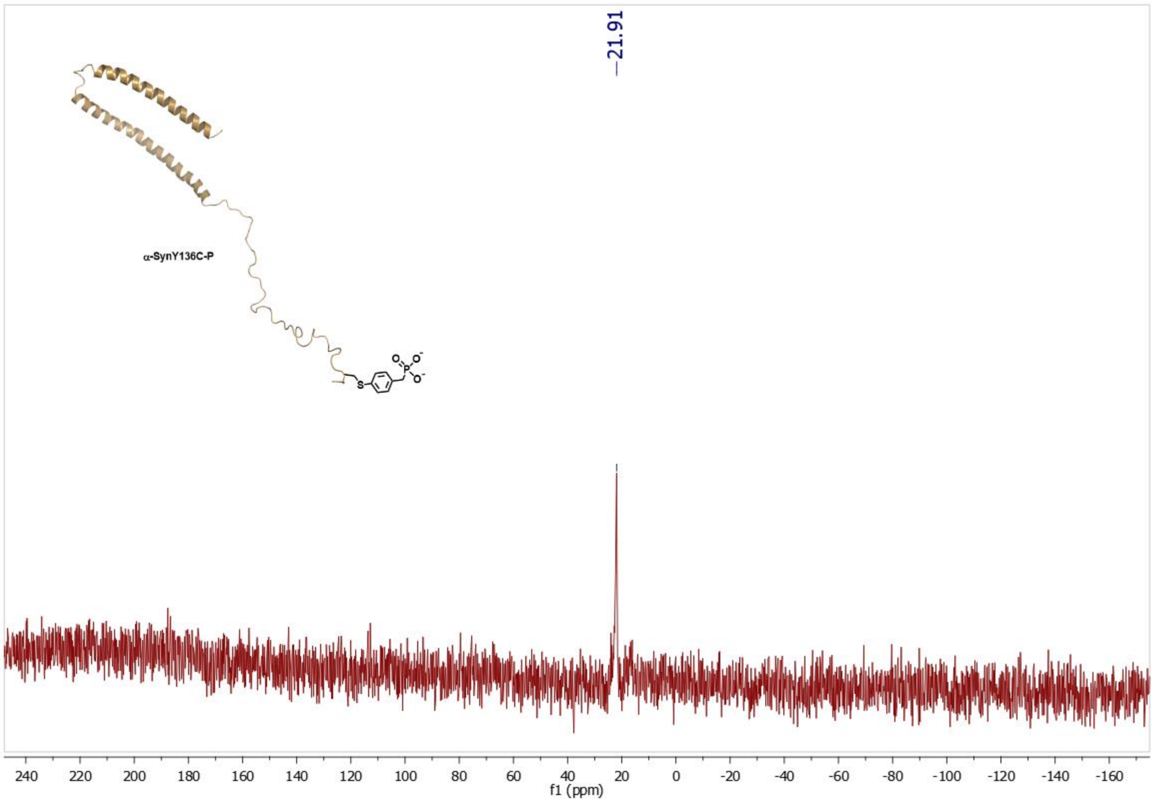

Figure S46. ^{31}P NMR spectrum of **α-SynY136C-P** in D_2O.

17. TEM micrographs

Expanded TEM micrographs

WT-α-Syn

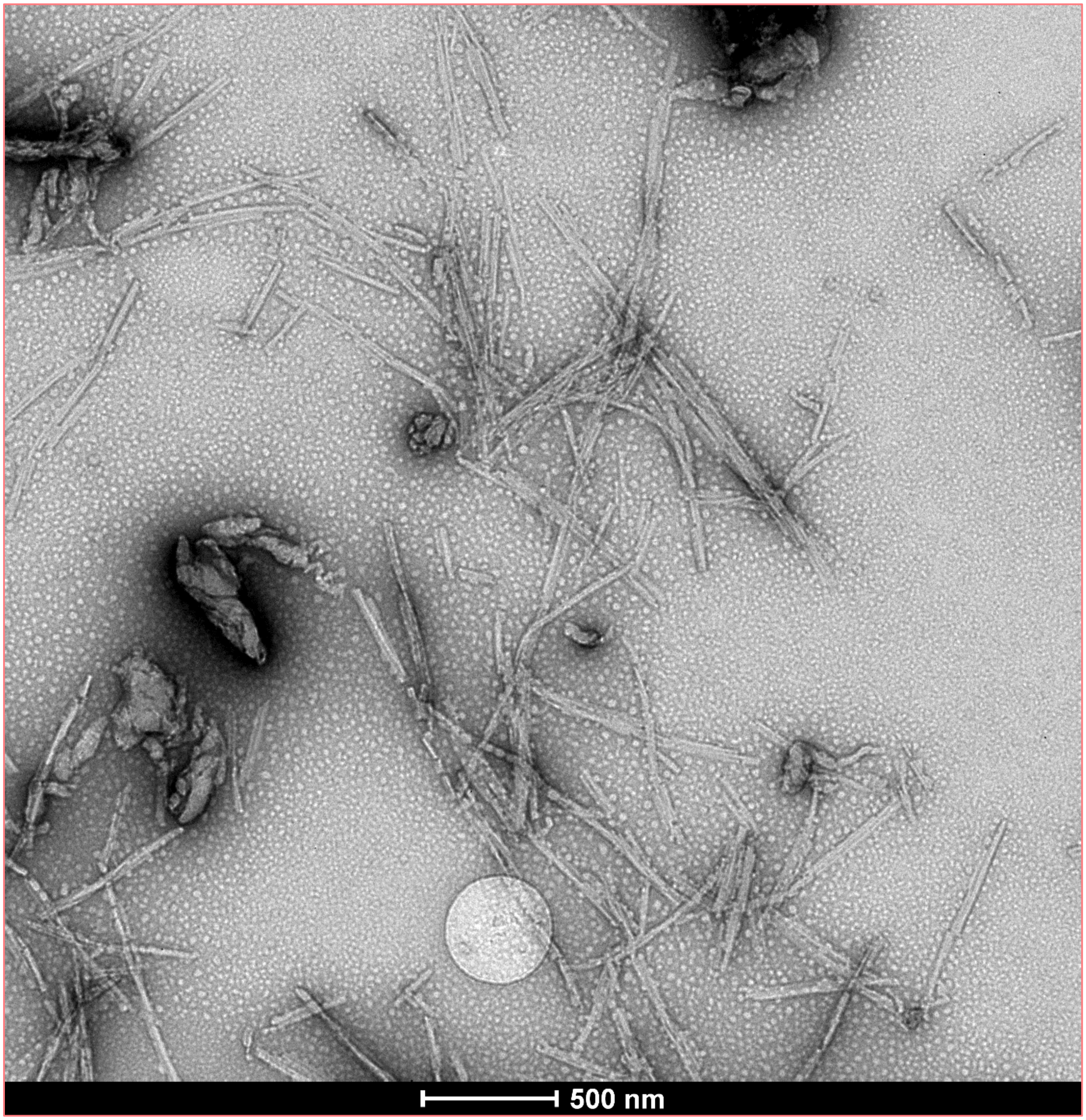

Y39C-α-Syn

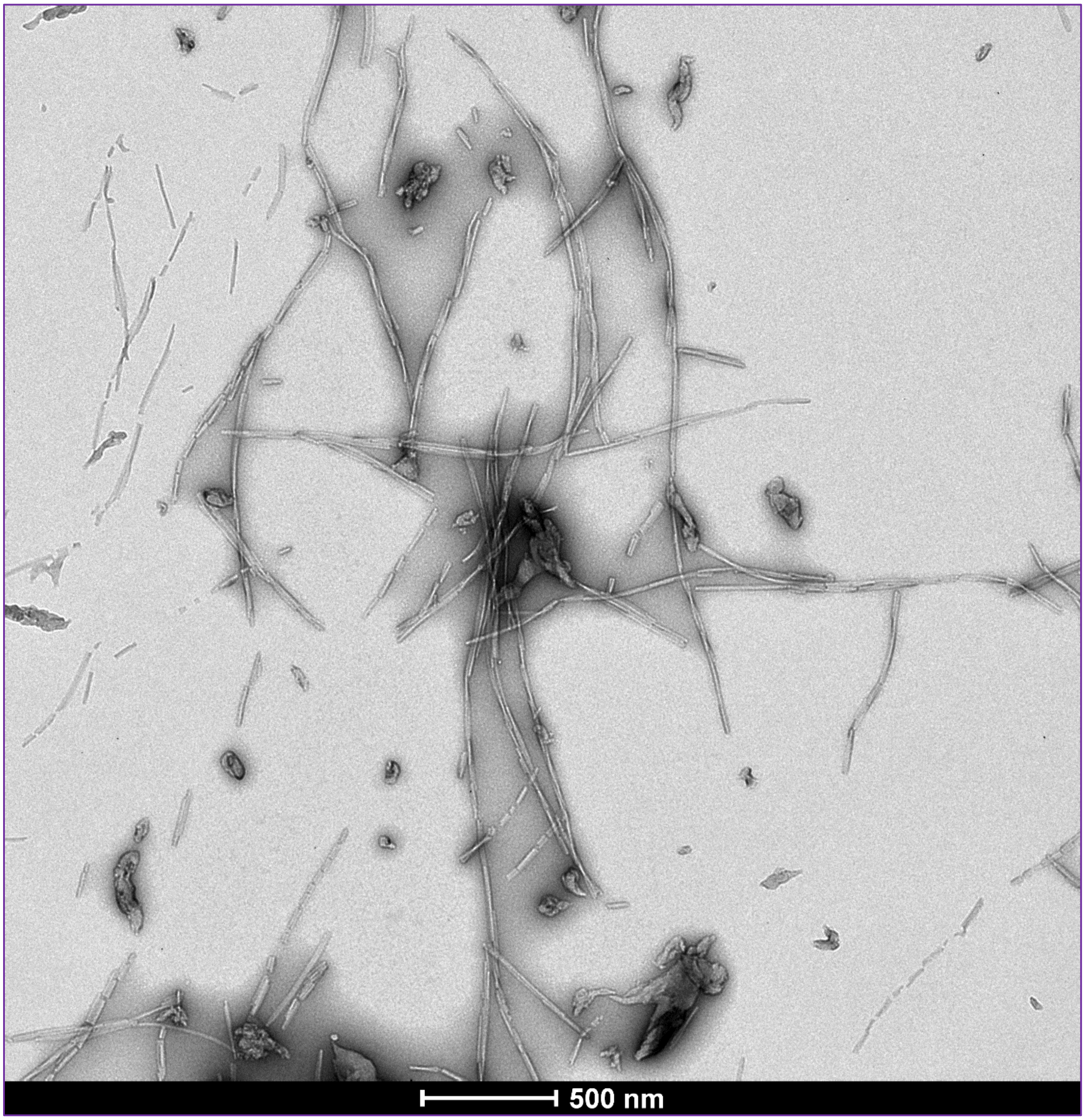

Y125C-α-Syn

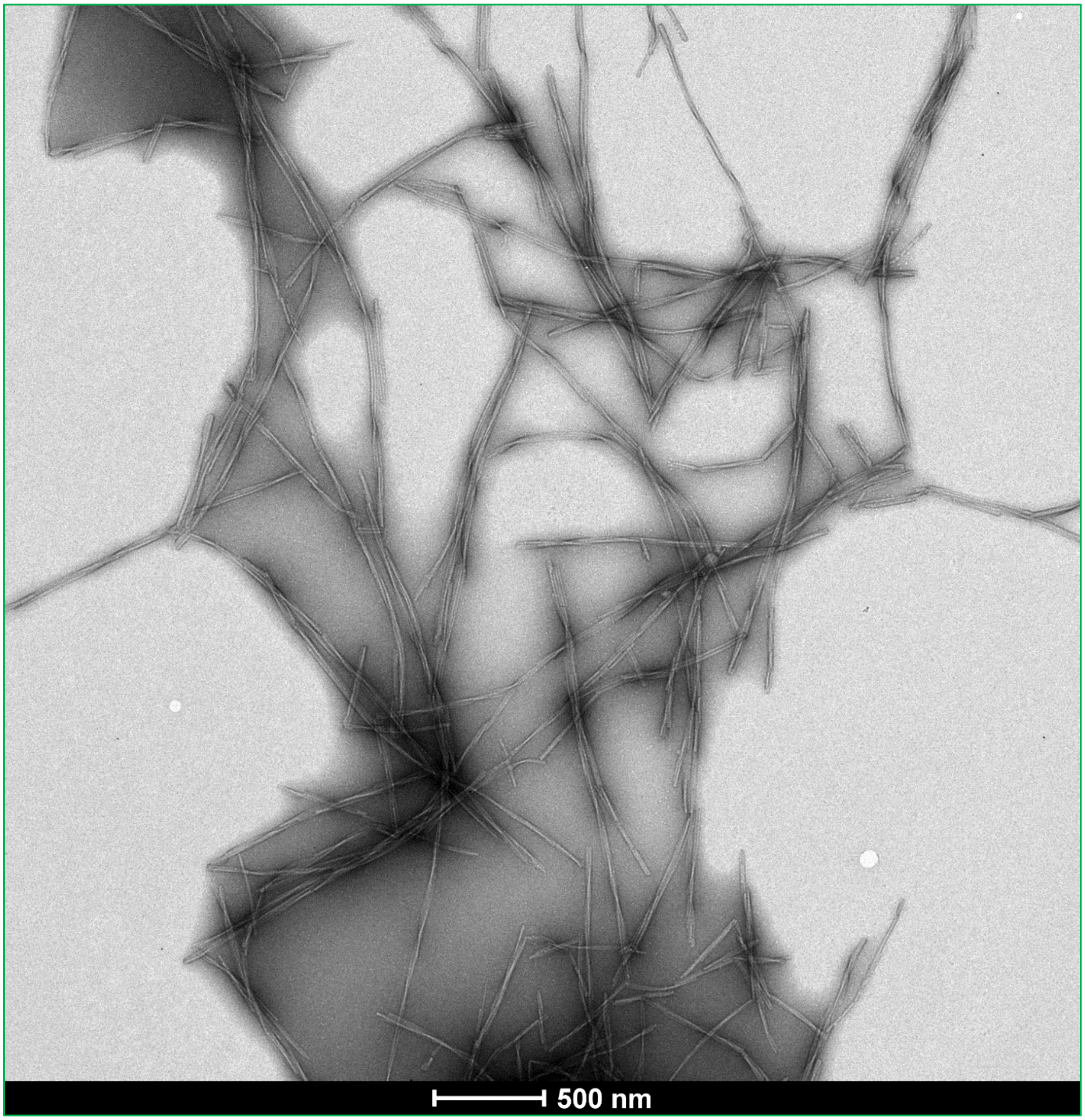

Y133C-α-Syn

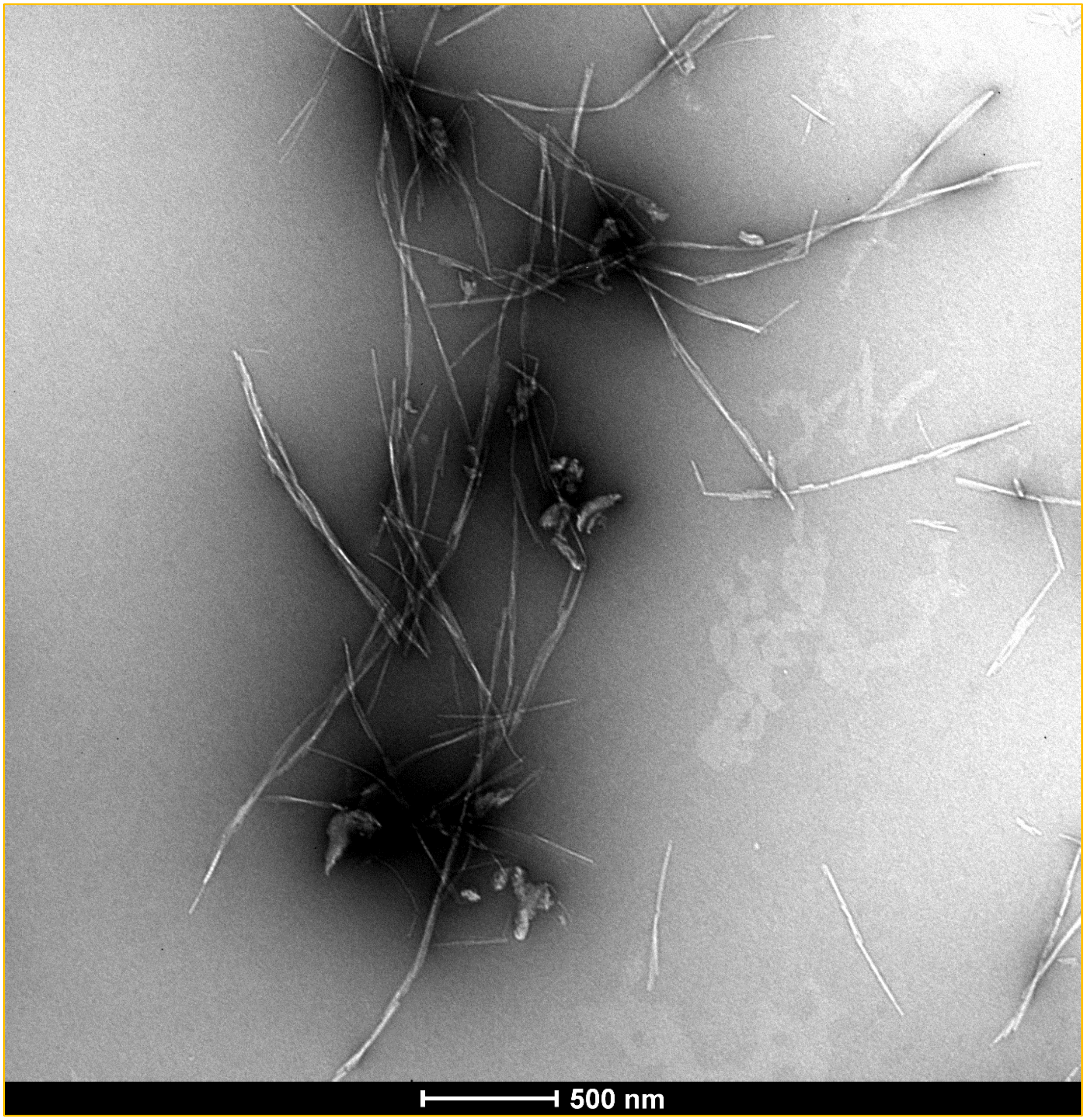

Y136C-α-Syn

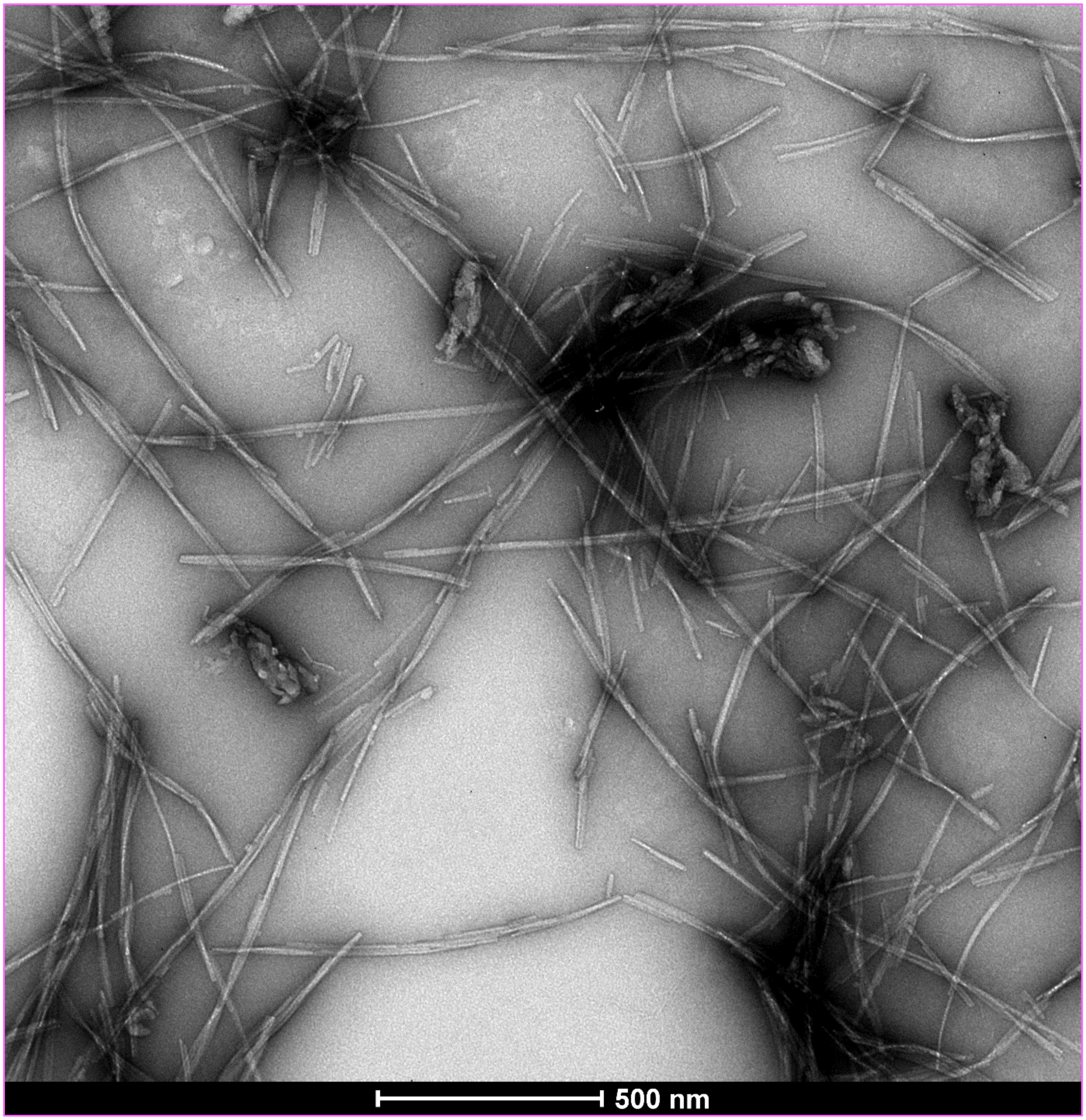

α-SynY39C-P

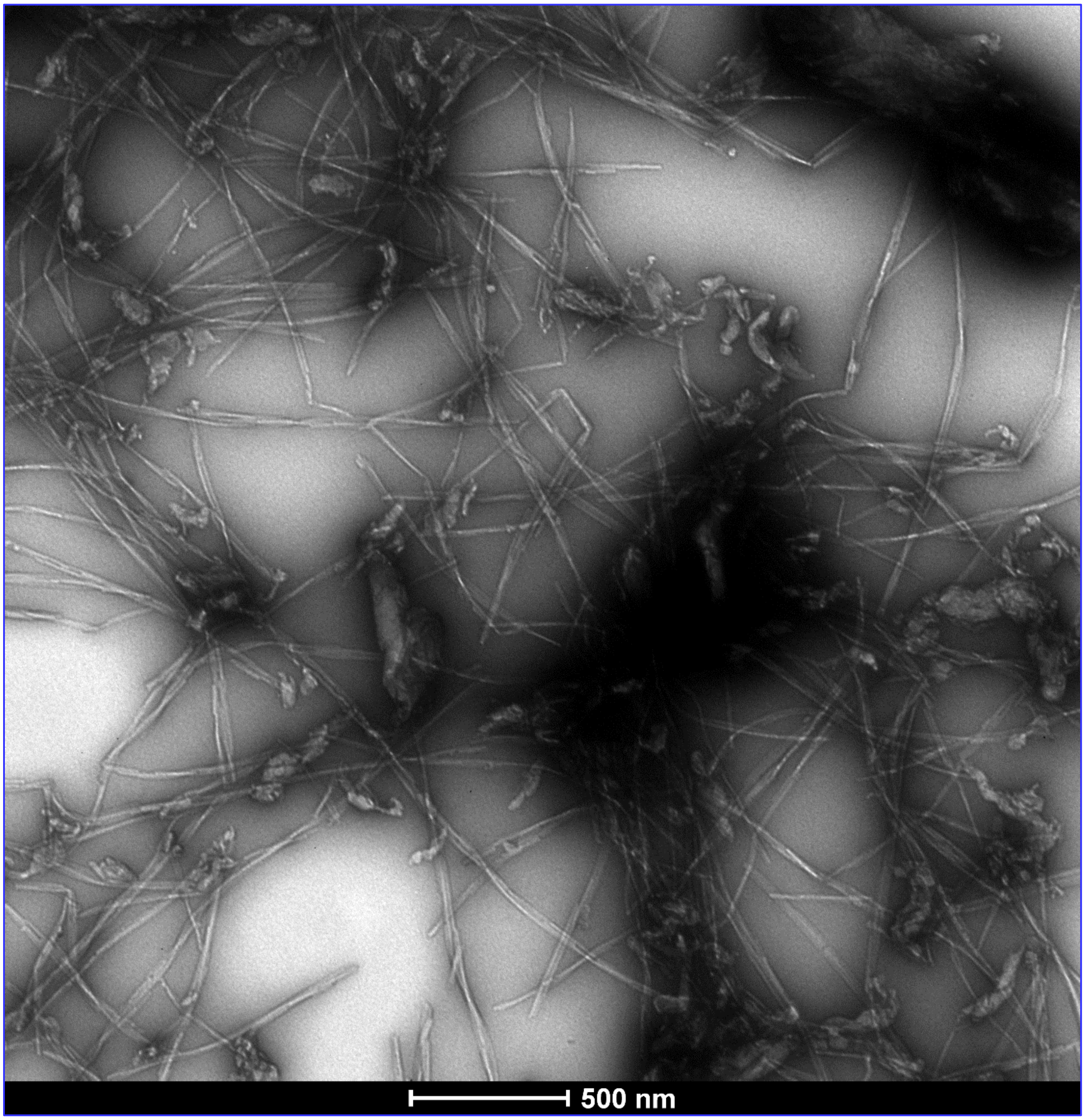

α-SynY125C-P

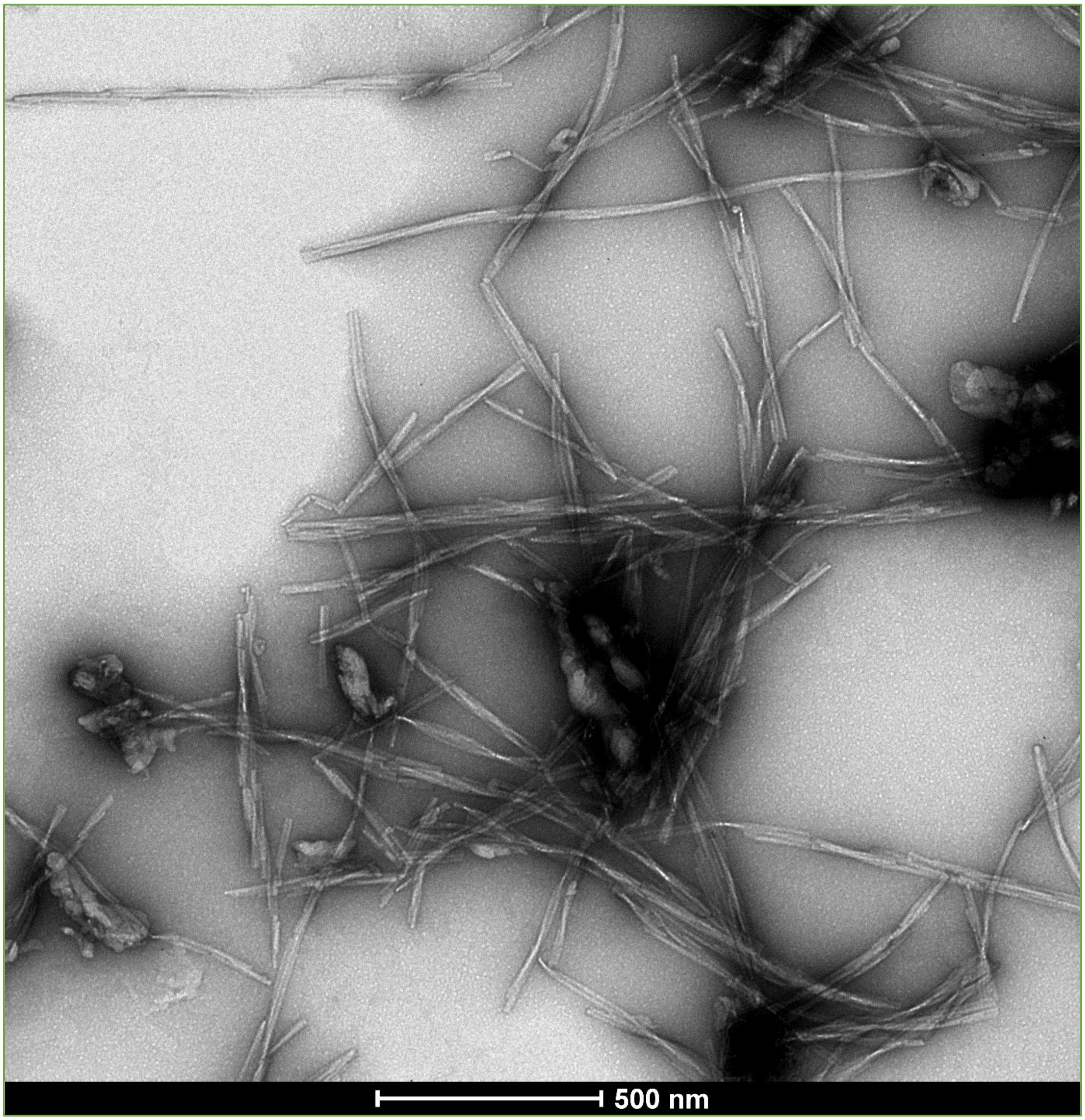

α-SynY133C-P

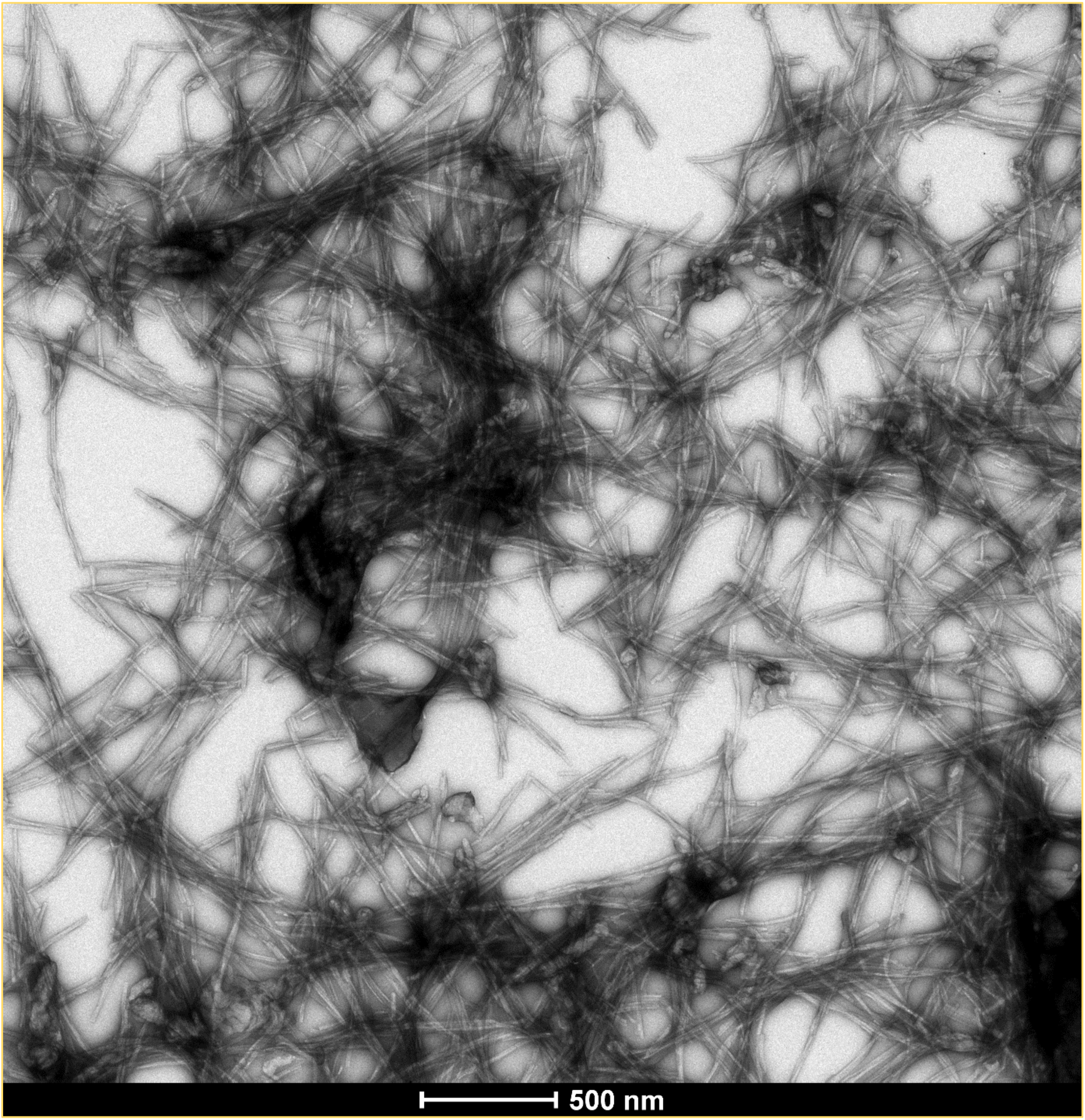

α-SynY136C-P

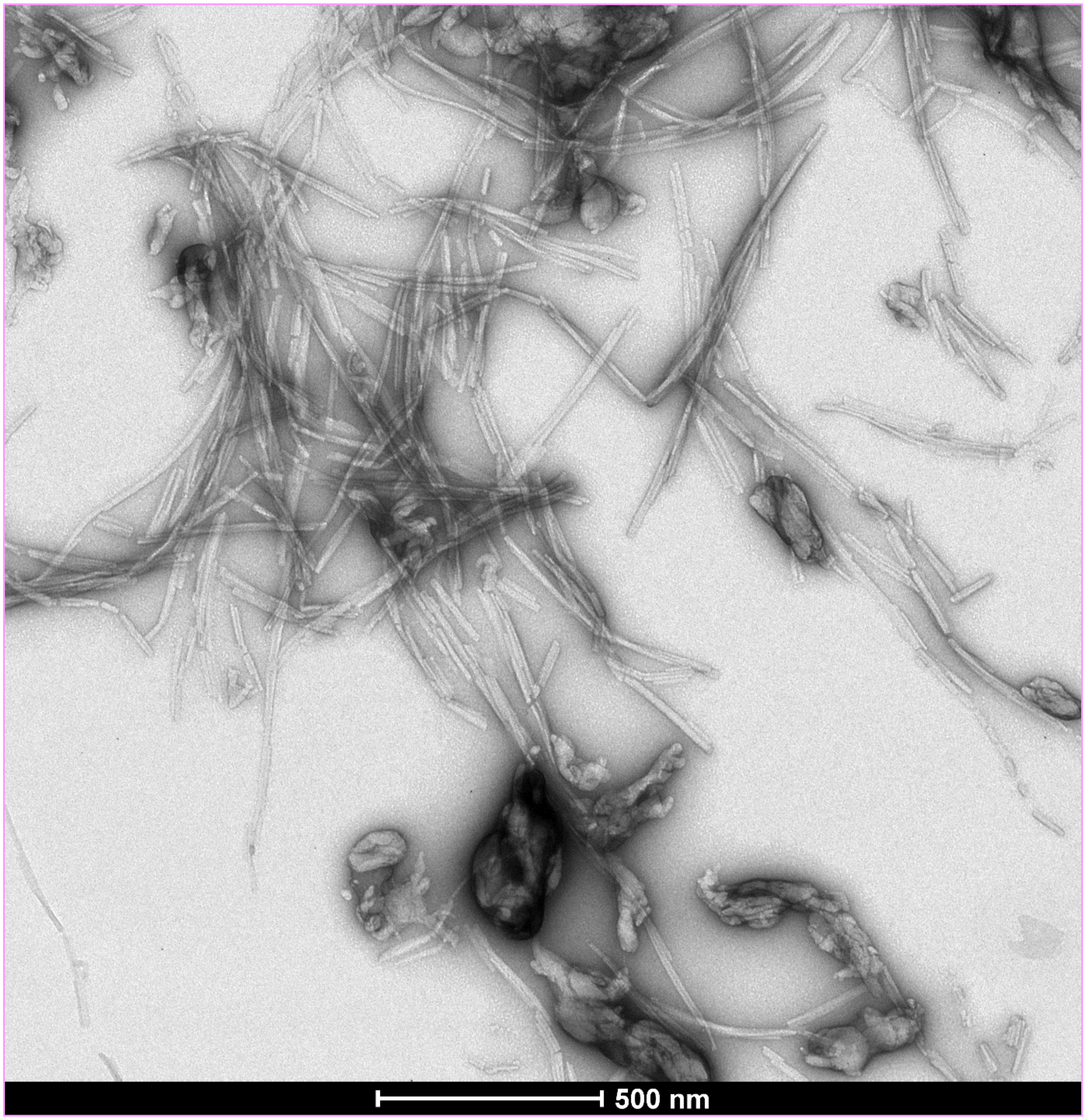

α-SynY39C-N

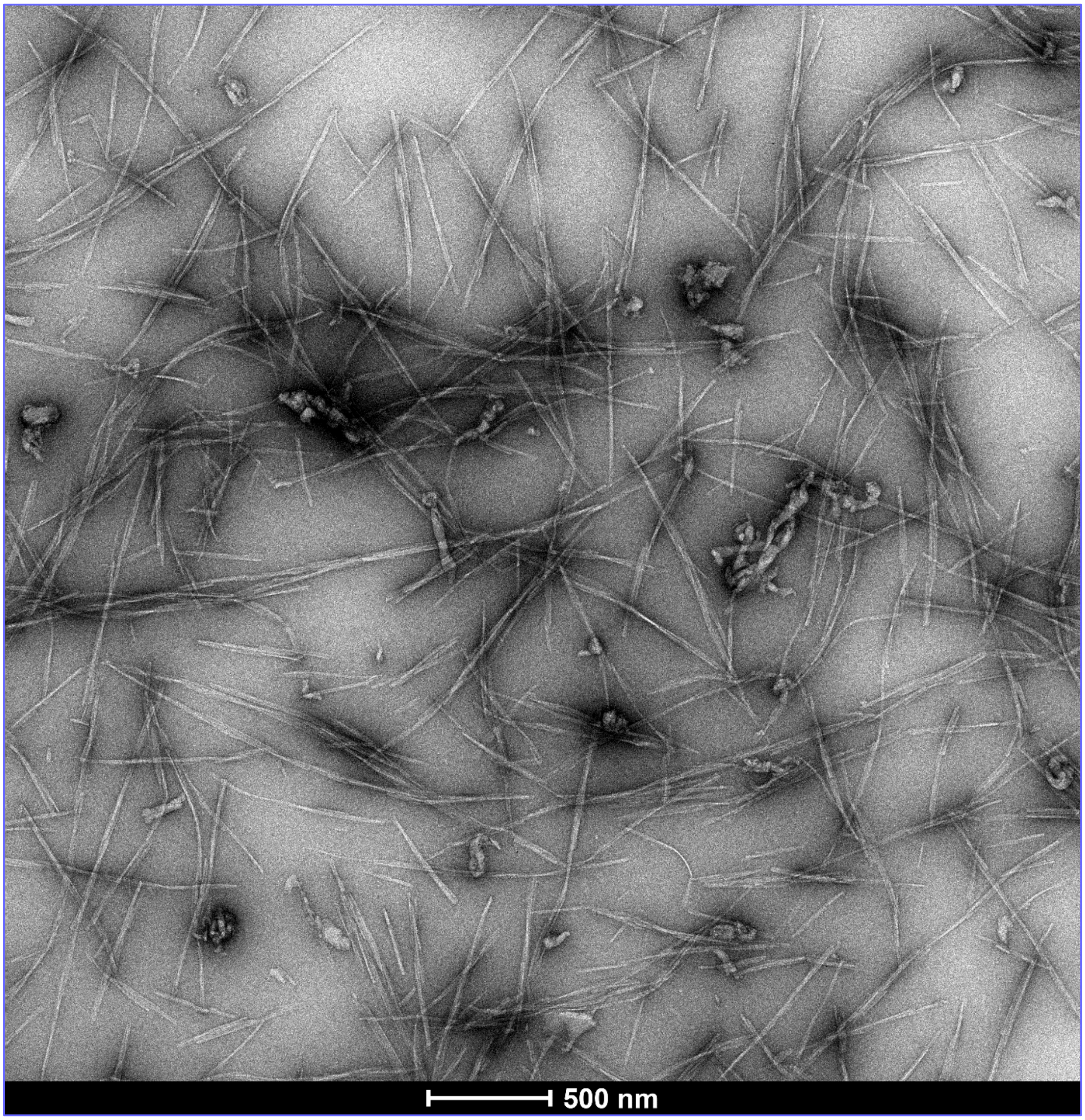

α-SynY125C-N

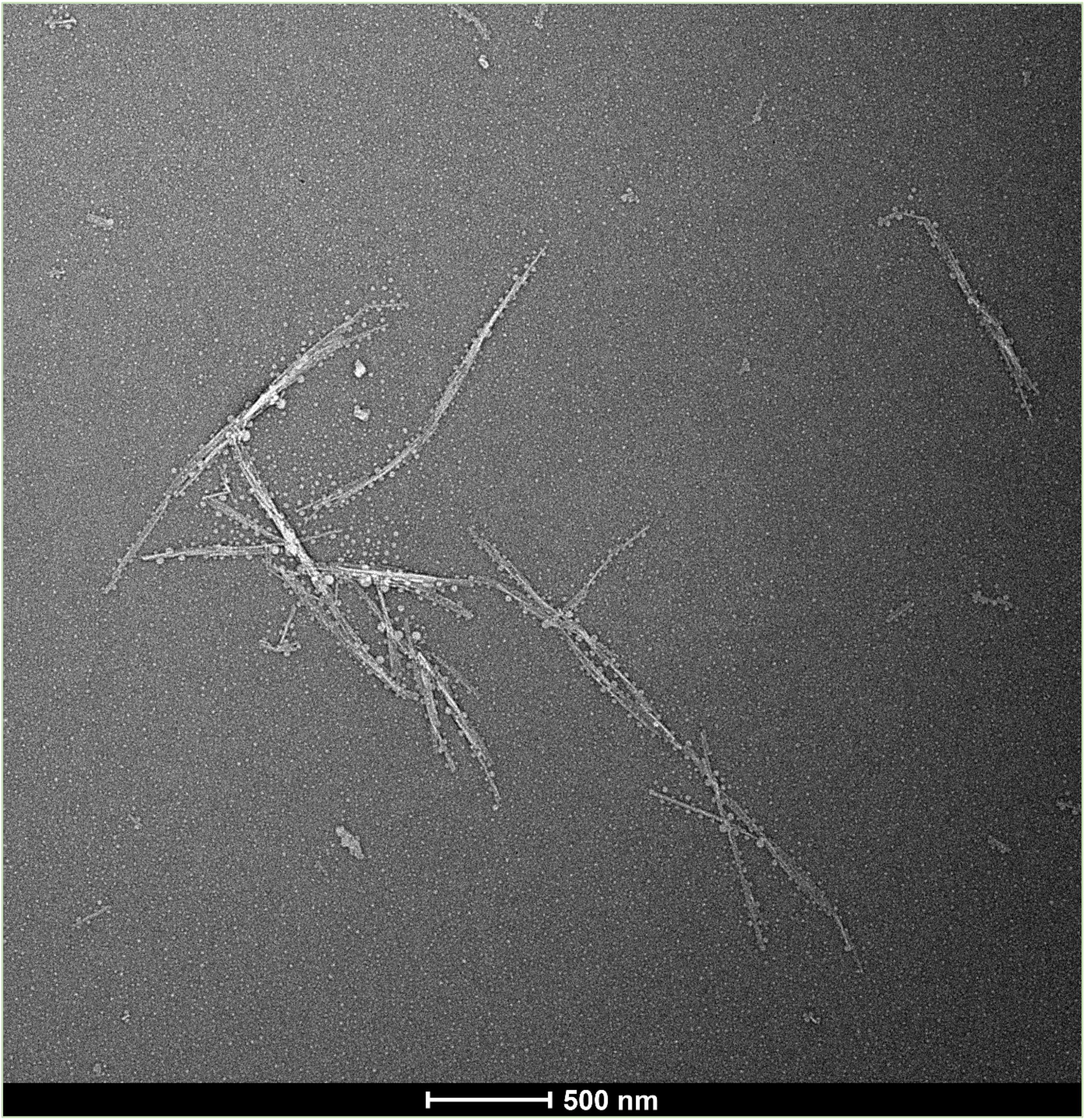

α-SynY133C-N

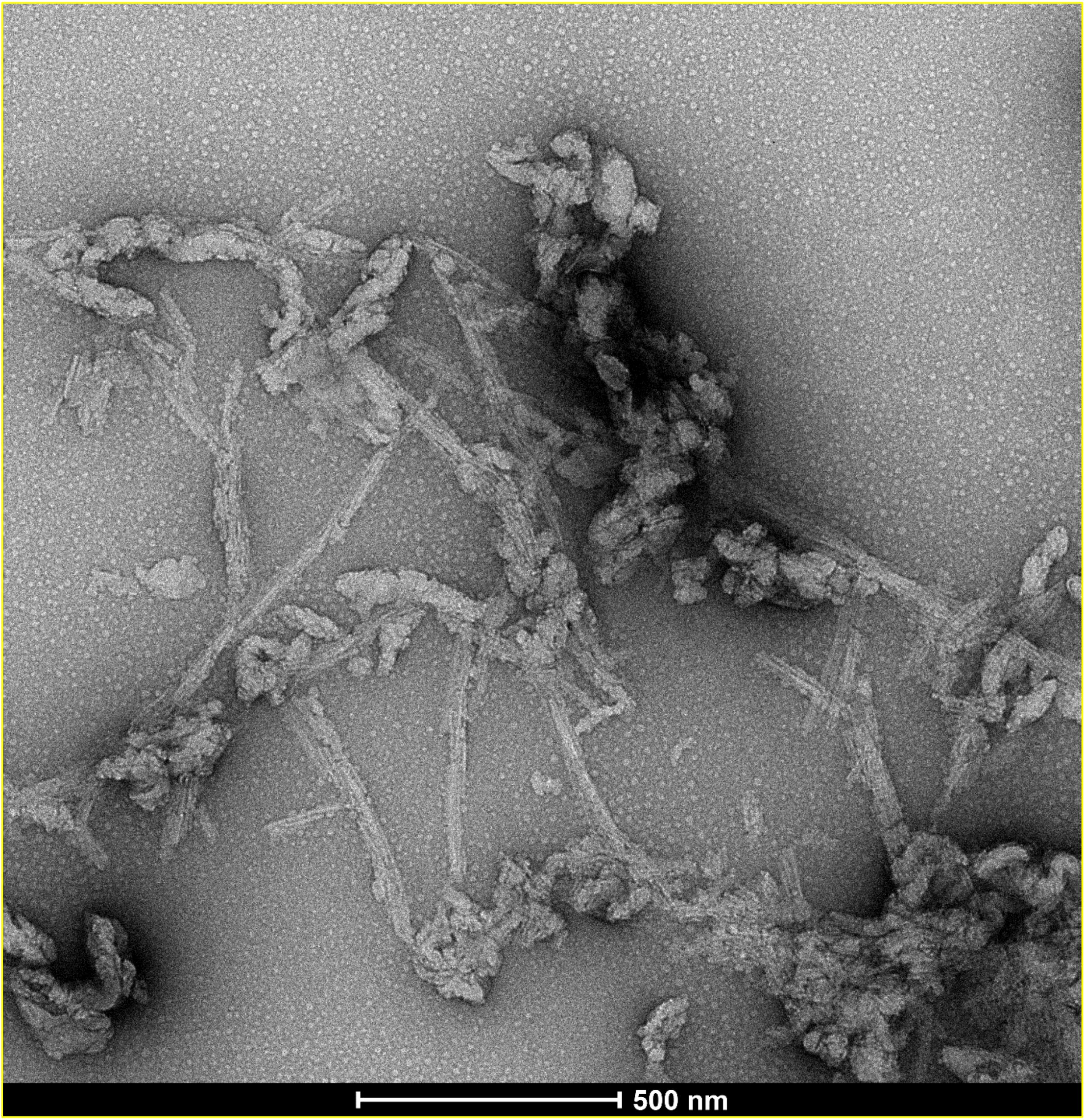

α-SynY136C-N

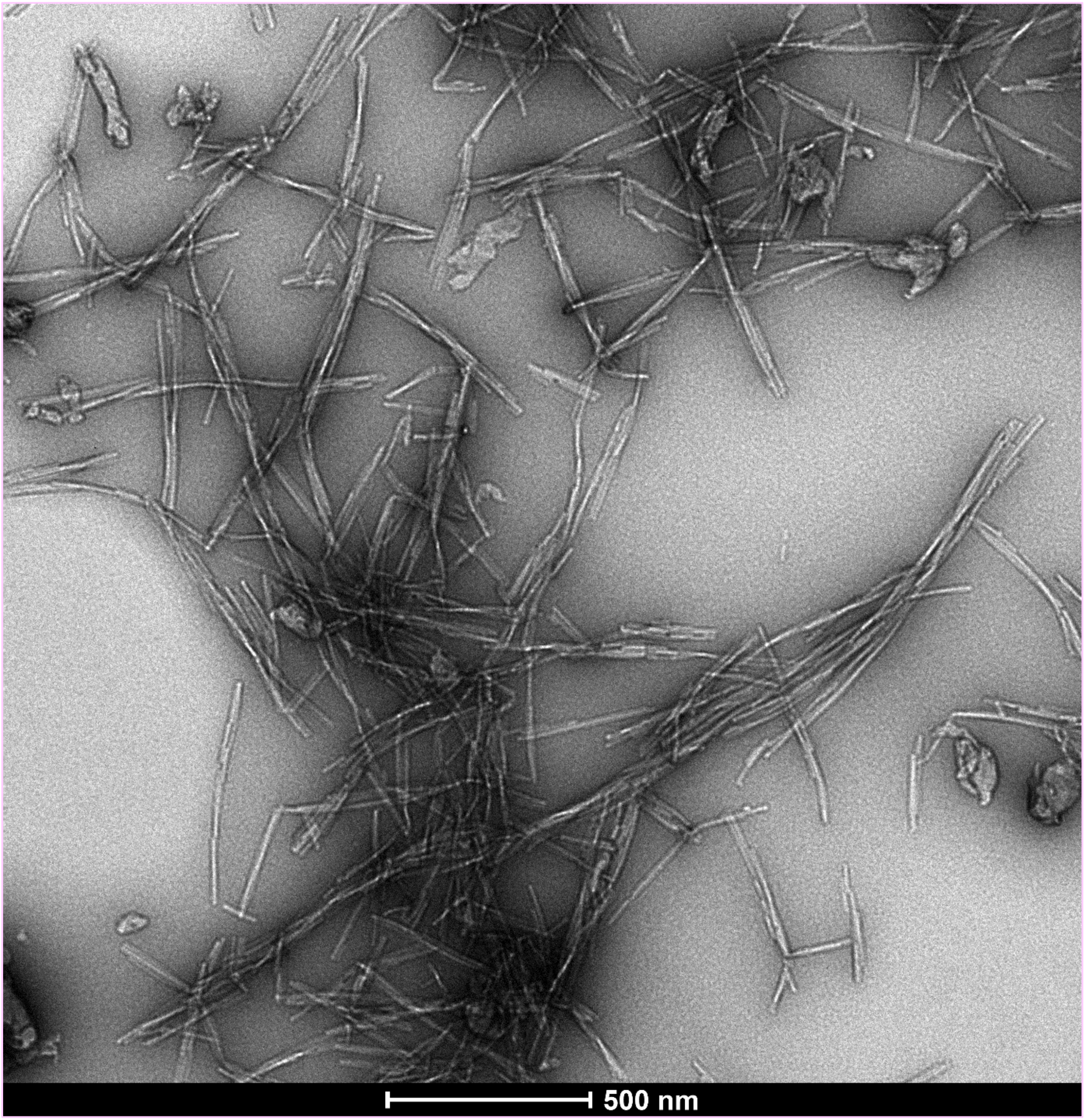

18. Crystal data and structure refinement for Pd-2

The crystals of **Pd-2** were obtained by vapor diffusion of a DCM solution of **Pd-2** with pentane at room temperature. The molecular structure of **Pd-2** in the solid state was unequivocally determined by a single-crystal X-ray diffraction analysis.[4,5]

[CCDC 2362585)]

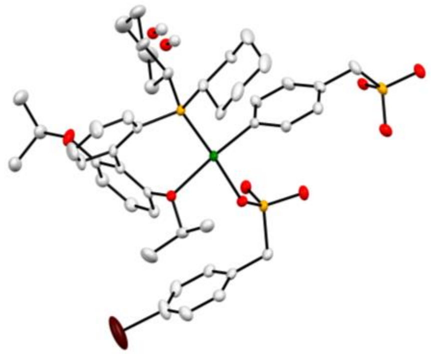

Bond precision: C-C = 0.0056 A Wavelength=0.71073

Cell:	a=11.4638(4)	b=13.1386(5)	c=16.9277(6)
	alpha=91.338(2)	beta=104.504(2)	gamma=91.979(2)
Temperature:	110 K		

	Calculated	Reported
Volume	2465.54(16)	2465.54(16)
Space group	P -1	P -1
Hall group	-P 1	-P 1
Moiety formula	2(C44 H58 Br O8 P3 Pd), C H4 O [+ solvent]	2(C44 H58 Br O8 P3 Pd), C H4 O
Sum formula	C89 H120 Br2 O17 P6 Pd2 [+ solvent]	C89 H120 Br2 O17 P6 Pd2
Mr	2020.27	2020.28
Dx,g cm-3	1.361	1.361
Z	1	1
Mu (mm-1)	1.332	1.326
F000	1042.0	1042.0
F000'	1040.44	
h,k,lmax	16,19,24	16,19,24
Nref	16576	16495
Tmin,Tmax	0.845,0.879	0.630,0.746
Tmin'	0.609	

Correction method= # Reported T Limits: Tmin=0.630 Tmax=0.746 AbsCorr = MULTI-SCAN

Data completeness= 0.995 Theta(max)= 31.617

R(reflections)= 0.0569(11661) wR2(reflections)= 0.1544(16495)

S = 1.033 Npar= 531

19. References

(1) Vinogradova, E. V.; Zhang, C.; Spokoyny, A. M.; Pentelute, B. L.; Buchwald, S. L. Organometallic Palladium Reagents for Cysteine Bioconjugation. *Nature* **2015**, *526*, 687−691.

(2) Lin, X.; Nithun, R. V.; Samanta, R.; Harel, O.; Jbara, M. Enabling Peptide Ligation at Aromatic Junction Mimics via Native Chemical Ligation and Palladium-Mediated S-Arylation. *Org. Lett.* **2023**, *25*, 4715-4719.

(3) Dawson, P. E.; Muir, T. W.; Clark-Lewis, I.; Kent, S. B. H. Synthesis of Proteins by Native Chemical Ligation. *Science* **1994**, *266*, 776−779.

(4) Kubota, K.; Takahashi, R.; Ito, H. Mechanochemistry Allows Carrying out Sensitive Organometallic Reactions in Air: Glove-box-and-schlenk-line-free Synthesis of Oxidative Addition Complexes from Aryl Halides and Palladium (0). *Chem. Sci*. **2019**, 10, 5837-5842.

(5) Pan, F.; Boursalian, G. B.; Ritter, T. Palladium-catalyzed Decarbonylative Difluoromethylation of Acid Chlorides at Room Temperature. *Angew. Chem., Int. Ed.* **2018**, *57*, 16871−16876.
